# Dipeptides Containing Pyrene and Modified Photochemically Reactive Tyrosine: Noncovalent and Covalent Binding to Polynucleotides

**DOI:** 10.3390/molecules28227533

**Published:** 2023-11-10

**Authors:** Igor Sviben, Mladena Glavaš, Antonija Erben, Thomas Bachelart, Dijana Pavlović Saftić, Ivo Piantanida, Nikola Basarić

**Affiliations:** Department of Organic Chemistry and Biochemistry, Ruđer Bošković Institute, Bijenička cesta 54, 10000 Zagreb, Croatia; igor.sviben@irb.hr (I.S.); mladena.glavas@irb.hr (M.G.); antonija.erben@irb.hr (A.E.); thomas.bachelart7@gmail.com (T.B.); dijana.pavlovic.saftic@irb.hr (D.P.S.)

**Keywords:** dipeptides, noncovalent binding to DNA/RNA, photochemistry, quinone methides, photochemical DNA/RNA alkylation

## Abstract

Dipeptides **1** and **2** were synthesized from unnatural amino acids containing pyrene as a fluorescent label and polynucleotide binding unit, and modified tyrosine as a photochemically reactive unit. Photophysical properties of the peptides were investigated by steady-state and time-resolved fluorescence. Both peptides are fluorescent (*Φ*_f_ = 0.3–0.4) and do not show a tendency to form pyrene excimers in the concentration range < 10^−5^ M, which is important for their application in the fluorescent labeling of polynucleotides. Furthermore, both peptides are photochemically reactive and undergo deamination delivering quinone methides (QMs) (*Φ*_R_ = 0.01–0.02), as indicated from the preparative photomethanolysis study of the corresponding *N*-Boc protected derivatives **7** and **8**. Both peptides form stable complexes with polynucleotides (log *K*_a_ > 6) by noncovalent interactions and similar affinities, binding to minor grooves, preferably to the AT reach regions. Peptide **2** with a longer spacer between the fluorophore and the photo-activable unit undergoes a more efficient deamination reaction, based on the comparison with the *N*-Boc protected derivatives. Upon light excitation of the complex **2**·oligoAT_10_, the photo-generation of QM initiates the alkylation, which results in the fluorescent labeling of the oligonucleotide. This study demonstrated, as a proof of principle, that small molecules can combine dual forms of fluorescent labeling of polynucleotides, whereby initial addition of the dye rapidly forms a reversible high-affinity noncovalent complex with ds-DNA/RNA, which can be, upon irradiation by light, converted to the irreversible (covalent) form. Such a dual labeling ability of a dye could have many applications in biomedicinal sciences.

## 1. Introduction

Oligopeptides have emerged as potent pharmaceuticals in the last few decades [1,2,3,4]. Their widespread use in the past was limited due to their sensitivity to proteolytic enzymes. However, structural modifications including *N*-methylation [5], incorporation of noncanonical amino acids [6,7,8] or transformation to cyclic peptides [9,10,11], enable a 3D structure that can selectively interact with the target receptors, and increase hydrolytic stability allowing for pharmaceutical applications. Thus, peptide-based drug conjugates have recently been used for drug delivery owing to their specific targeting, cell penetration and self-assembled capacity [12,13]. Furthermore, the noncovalent binding of peptides and DNA is an important biological process, which enables transcription of DNA and cell replication [14,15]. Therefore, it is of utmost importance to gain knowledge in selective recognition of nucleobase sequences by oligopeptides leading to gene transcriptions [16,17], since affecting these events provides an immense scientific advantage, enabling numerous applications in biochemistry, biology and medicine. Therefore, the binding of small oligopeptides to polynucleotides has been intensively investigated [18], as well as DNA-binding oligopeptides which can be modulated by light [19].

Since canonic amino acids are involved in polynucleotide recognition, their incorporation in oligopeptides can be used in DNA sensing, as was demonstrated for tripeptides containing bis-tryptophan units [20]. Furthermore, peptide-binding units can easily be functionalized by fluorophores [21,22], providing tools for DNA fluorescent labeling and visualization [23]. Thus, Schmuck et al. used oligopeptides containing lysines for binding to polynucleotides, whereas incorporation of tryptophan and pyrene [24] was responsible for the fluorescence response, or fluorescence resonance energy transfer (FRET) between the naphthalene and the dansyl units [25]. The same group also developed a series of modified peptides with incorporated aminonaphthalimide as a fluorophore [26]. Furthermore, the binding of a series of fluorophore-modified oligopeptides to polynucleotides has been investigated [27,28,29], where the binding units were phenantridines [27], pyrenes [28], naphthalenedimide [30], guanidinocarbonylpyrroles [27] or coumarins [31]. 

We have recently developed a series of oligopeptides that bind noncovalently to polynucleotides and induce a fluorescence response owing to the phenantridine [31] or ditryptophan unit [32]. In addition, these oligopeptides contained a photochemically reactive modified tyrosine [33], which upon irradiation underwent deamination forming quinone methide (QM) intermediates, allowing for the covalent attachment of the fluorescence label to the polynucleotides. QMs are ubiquitous reactive intermediates in the chemistry and photochemistry of phenols [34] that have been intensively investigated owing to their applications in synthesis [35,36] and biological activity [37,38]. The biological activity of QMs was connected to their reactivity with proteins [39,40,41], nucleobases [42], DNA [43,44,45,46], which is reversible [47,48,49], and G-quadruplexes [50,51,52,53]. Moreover, QMs can be generated in photochemical reactions under mild conditions [54,55], which enables the spatial and temporal control of their generation and further applications in photo-pharmacology [56].

**Figure 1:**
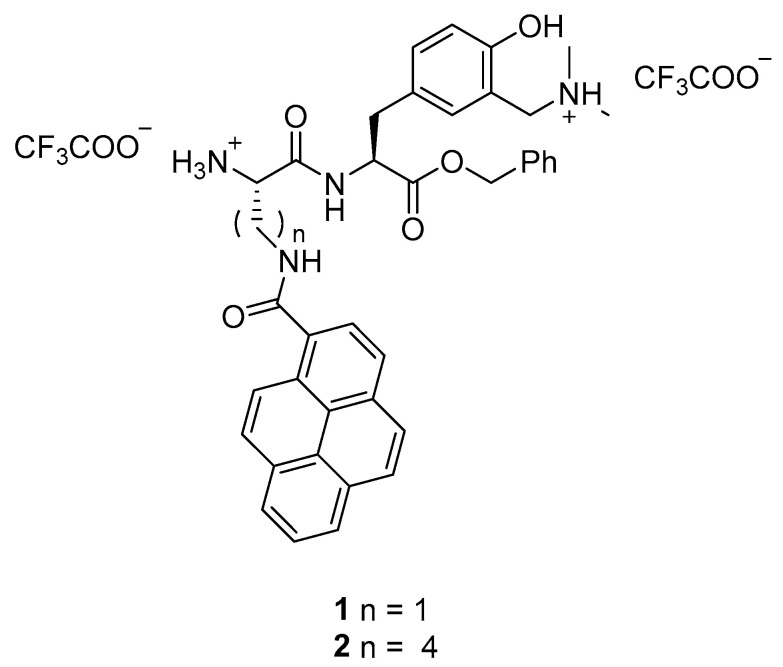
Structure of the investigated dipeptides **1** and **2**.

Herein we present the synthesis of two oligopeptides **1** and **2** (Figure 1) containing pyrene as a polynucleotide recognition unit and a fluorophore, and modified tyrosine which should enable photo-activation and covalent attachment to polynucleotides. We investigated the photophysical properties of **1** and **2** and their photochemical reactivity. The noncovalent binding to polynucleotides was assayed by model double-stranded (ds-) DNA and ds-RNA polynucleotides, differing in base pair composition, and consequently, in secondary structure and binding site properties (Table S6 in the Supplementary Materials).

The photo-attachment to polynucleotides upon light irradiation was demonstrated on model oligonucleotides by HPLC analysis of the irradiated mixtures. The molecules were strategically designed to probe for the effect of the different length of an alkyl linker between the polynucleotide binding unit (pyrene) and the photo-attachment unit (modified tyrosine) on the photochemical reactivity and binding ability to polynucleotides. Namely, depending on the linker length, two chromophores may interact (in the ground or in the excited state), leading to different photo-reactivity. The effect of the aromatic moiety in the phenylalanine and tyrosine in the NH-*π* interaction with the peptide back-bone has been investigated [57]. Furthermore, the formation of exciplexes between the pyrene and amines has also been well documented [58]. Thus, dipeptides containing modified tyrosine and pyrene could, in principle, be used as build-in motives with the desired conformation and/or photophysical properties. Moreover, the separation between two chromophores in the peptide may affect its binding aptitude to polynucleotides. For example, to achieve bis-intercalation, the binding chromophores have to be separated by a linker of a certain length to allow for the exclusion principle [59]. The results presented are important in the rational design of fluorescent labels that can covalently attach to polynucleotides upon photochemical activation.

## 2. Results and Discussion

### 2.1. Synthesis

**Scheme 1:**
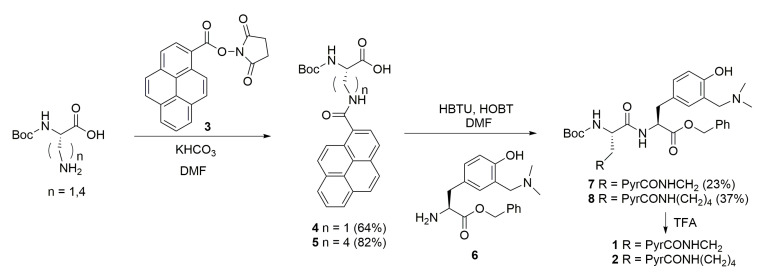
Synthesis of investigated dipeptides **1** and **2**.

The pyrene amino acids **4** and **5** were prepared in good to excellent yields by use of *N*-hydroxysuccinimide (NHS) and the 1-ethyl-3-(3-dimethylaminopropyl)carbodiimide (EDC) activation protocol [60]. In the first step, pyrene-1-carboxylic acid was activated by EDC and transformed to the succinimide ester **3**, which was used in the coupling with *N*-Boc protected amino acids (Scheme 1). The synthetic protocol for the preparation of dipeptides **1** and **2** was based on the standard Boc-chemistry in solution from pyrene amino acids **4** or **5** and Bn-protected modified tyrosine **6** by use of *N*,*N*,*N*′,*N*′-tetramethyl-*O*-(1H-benzotriazol-1-yl)uronium hexafluorophosphate (HBTU) and 1-hydroxybenzotriazole (HOBT) as coupling reagents [61]. The dipeptides **7** and **8** were obtained in modest to moderate yields, and they were transformed to the corresponding salts **1** and **2** by a treatment with TFA.

We attempted also to prepare a pyrene alanine derivative with a shorter linker between the pyrene and the modified tyrosine (structure F in Scheme S2 in the Supplementary Materials). However, an attempt to couple Boc-L-alanine with the modified tyrosine **6** by use of different coupling reagents failed in our case (Scheme S1 and Table S1 in the Supplementary Materials). Similarly, synthetic protocols where the carboxylic acid in pyrene alanine D was activated and attempted to couple with Tyr or modified Tyr afforded dipeptide in an unsatisfactory yield and in the form of two diastereomers (Scheme S2 in the Supplementary Materials).

### 2.2. Photophysical Properties

**Figure 2:**
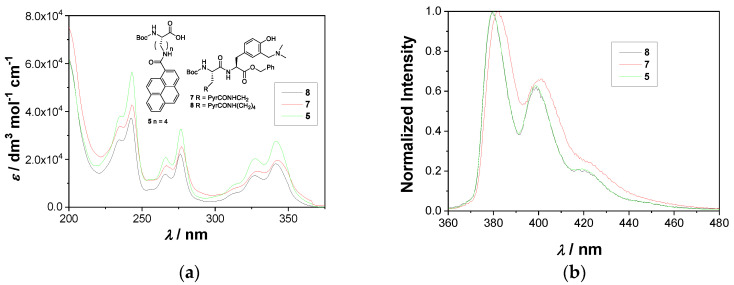
(**a**) Absorption spectra of compounds **5**, **7** and **8** in CH_3_CN; (**b**) normalized emission spectra (*λ*_ex_ = 310 nm) of **5**, **7** and **8** in CH_3_CN.

For the application of fluorescent dyes in theragnostics it is important to investigate their photophysical properties. Since it was anticipated that **1** and **2** may form aggregates in the aqueous solution (vide infra) due to the inherent property of the pyrenes to stack by *π*,*π* interactions and form excimers [62], we also investigated the model compounds, dipeptides **7** and **8**, in nonaqueous CH_3_CN solution. Both compounds are composed of two chromophores/fluorophores (pyrene and modified tyrosine) so for the comparison we also investigated compound **5** which contains the pyrene chromophore only. The absorption and fluorescence spectra measured in CH_3_CN are shown in Figure 2 (for all photophysical data in all solvents see Figures S1–S13 in the Supplementary Materials).

The absorption spectra of all three compounds show maxima at ≈240, 270 and 335 nm, typical for pyrene chromophore, whereas the modified amino acid, Tyr[CH_2_N(CH_3_)_2_], has only a minor influence on the absorption spectra. In addition, none of the compounds showed a tendency to aggregate in the CH_3_CN solution in the investigated concentration range (up to 1.5 × 10^−5^ M) based on the linear dependence of the absorbance on the concentration.

The fluorescence spectra in CH_3_CN are characterized by two bands with maxima at ≈380 and 400 nm, typical for pyrene. The position of the maxima and the shape of the spectra did not depend on the excitation wavelength (Figures S1–S3 in the Supplementary Materials), indicating that the emission from modified tyrosine was not detected by the steady-state fluorescence. However, the fluorescence spectra of **7** in CH_3_CN exhibit bathochromic shifts of 5 nm compared to **8** and **5**, which suggests that in the dipeptide with a shorter linker some intramolecular interaction between pyrene and tyrosine chromophores in the excited states takes place. (Figure 1). The quantum yields of fluorescence (*Φ*_f_) were measured by use of quinine sulfate in H_2_O/H_2_SO_4_ as a reference (*Φ*_f_ = 0.546) [63], and the values are similar 0.3–0.5 (Table 1). Note that the reported values contain a large error, which is due to the fact that we used the comparative method for determination of *Φ*_f_ [64]. Moreover, molecules **7** and **8** are bi-chromophoric; thus, at wavelengths where more pyrene is excited, the *Φ*_f_ appears to have a larger value.

The decay of fluorescence in CH_3_CN solutions was measured by time-correlated single-photon counting (TC-SPC). The decays of fluorescence for **7** and **8** were measured at 400 nm, where both tyrosine and pyrene emit, but the excitation was at 280 nm where both chromophores absorb light, and at 340 nm where only pyrene absorbs light (Table 1, for the decays see Figures S7–S9 in the Supplementary Materials). The decays for **7** and **8** were fit to a sum of three exponents with similar values of the decay times (the same within the experimental error), regardless of the excitation wavelength. Such a finding indicates that the pyrene is the emissive chromophore, as was also suggested by the steady-state spectra. Furthermore, similar values of decay times with their similar contributions, irrespective which chromophore was excited, suggest that the fluorescence resonance energy transfer (FRET) from the modified tyrosine to the pyrene either does not take place, or it is ultrafast and very efficient. Note that we have already designed bichromophoric molecules composed of naphthalimides and modified tyrosine, where the FRET was responsible for the photochemical stability of the modified tyrosine [65]. However, **7** and **8** react in the photomethanolysis (vide infra), suggesting that FRET is inefficient. Note that the emission spectrum of the modified tyrosine (300–350 nm) [36] overlaps with the lowest energy absorption band of the pyrene (Figure 2), and two chromophores are at a very short distance. Thus, the plausible reason for the inefficient FRET may be the unfavorable orientation of the transition dipole moments between the chromophores. Note also that multiexponential decay for **7**, **8** and **5** is probably due to the formation of aggregates, even though the absorption and the steady-state fluorescence spectra did not suggest aggregation.

For the application of **1** and **2**, their photophysical properties in aqueous solution are important. Thus, we measured their absorption and fluorescence spectra in aqueous cacodylate buffer (pH = 7.0, 50 mM), with 0.05–0.5% DMSO added to assure solubility (Figures S10–S13). The spectra were measured at different concentrations to investigate if aggregation takes place, and in which concentration range the fluorescence response depends linearly on concentration. The steady-state fluorescence spectra did not indicate the formation of aggregates and the linear fluorescence response was observed for **1** up to 1.5 × 10^−5^ M, and for **2** up to 3 × 10^−5^ M. Thus, based on similar spectral properties and no evidence of aggregation, one can assume that **1** and **2** in aqueous solution have similar steady-state fluorescence properties to **7** and **8** in CH_3_CN.

### 2.3. Photochemical Reactivity

**Scheme 2:**
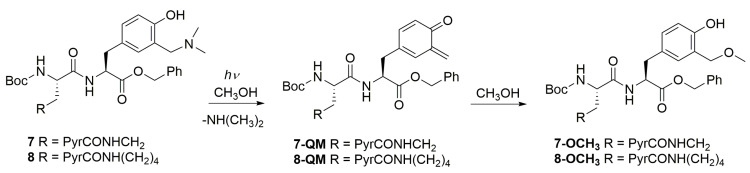
Photomethanolysis of model dipeptides **7** and **8** indicates formation of QM intermediates.

Photodeamination of aminomethylphenol groups in methanol leads to the photomethanolysis products via QM intermediates [35,36]. Therefore, we investigated the photochemical reactivity of dipeptides **7** and **8**, which were chosen to facilitate the isolation of the methanolysis photoproducts. The irradiations were conducted in CH_3_OH solution at 300 nm over 1 h, and the composition of the solutions was analyzed by HPLC. The conversion after 1 h irradiation was 70% and 89%, respectively, whereas the corresponding photoproducts **7-OCH_3_**, and **8-OCH_3_** (Scheme 2) were isolated by TLC and characterized by NMR. The detection of the methanolysis photoproducts is a strong indication that photodeamination takes place, delivering QMs. The efficiency of the photomethanolysis reaction (*Φ*_R_) was measured upon excitation at 254 nm by the use of KI/KIO_3_ actinometer (*Φ*_254_ = 0.74) [63,66] (Table 2). The photoreaction is not efficient, but the fact that it takes place indicates that the FRET from the modified tyrosine to the pyrene is not efficient. Furthermore, the photoreaction is about two times more efficient for derivative **8** with a longer spacer between chromophores.

### 2.4. Noncovalent Binding to Polynucleotides

Noncovalent binding of the investigated peptides to polynucleotides was investigated by thermal denaturation experiments, fluorescence titrations and CD spectroscopy. Noncovalent binding is important to ensure that the QM precursor is at proximity to the polynucleotide [47], since after QM generation its competing hydrolysis [67,68,69,70] destroys the active reagent and diminishes reactivity with the target.

#### 2.4.1. Thermal Denaturation Experiments

The thermal denaturation experiments provide information about the stabilization of double-stranded (ds-) polynucleotide helix thermal stability by small molecules [71]. The difference between the melting temperature of a free ds-polynucleotide (*T*_m_, the temperature at which 50% of the polynucleotide is in the form of a ds and single-stranded, ss) and its complex with a small molecule (Δ*T*_m_ value) characterizes the interaction, with Δ*T*_m_ > 5 °C generally supporting intercalative or minor groove binding interactions [72]. The experiments were conducted with *ct*-DNA (*calf thymus*-DNA), which is a good model containing both AT and GC regions, and with pApU, which is a model for RNA (see Figures S14 and S15 and Table S7 in the Supplementary Materials). However, compounds **1** and **2** showed very weak stabilization of *ct*-DNA and pApU, suggesting that they form non-specific interactions with the ds-helix. The irradiation of **1** or **2** in the presence of polynucleotides (300 nm, 8 lamps, 15 min) also did not significantly affect the *T*_m_ values.

#### 2.4.2. Fluorescence Titrations

**Figure 3:**
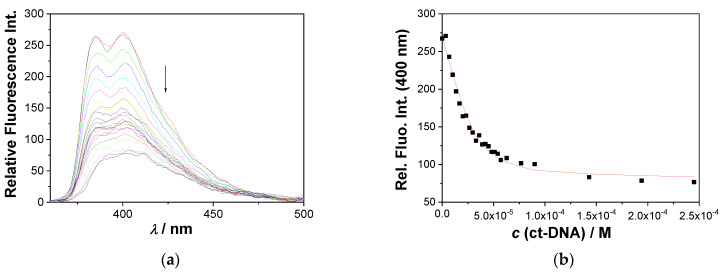
(**a**) Fluorescence titration for **2** (*c* = 1 × 10^–6^ M, *λ*_exc_ = 330 nm) with increasing *ct*-DNA concentration (*c* = 0 M, top black curve to *c* = 2.5 × 10^−4^ M, bottom dark grey curve) in an aqueous cacodylate buffer (pH 7.0, 50 mM at 25 °C); (**b**) fluorescence intensity at 400 nm from (**a**); ■ the experimental values, – calculated fit according to the Scatchard model (R^2^ = 0.97, fixed value *n* = 0.3).

The addition of any of the studied ds-DNA or ds-RNA resulted in the quenching of pyrene fluorescence in **1** and **2** (Figure 3 and Figures S16–S22 in the Supplementary Materials). The quenching of pyrene emission supports the interactions of the pyrene moiety with DNA or RNA, which facilitates the non-radiative decay of the excited pyrene, whereby loss of the well-defined emission bands and extensive broadening suggest electronic interaction of the pyrene with heteroaromatic nucleobases. However, the observed emission change does not allow for a distinction between the pyrene/nucleobase face-to-face interaction (intercalation into DNA/RNA) or edge-to-face interaction (DNA/RNA groove binding). The change in the fluorescence intensity allowed calculation of the binding constants by nonlinear regression analysis according to the Scatchard model (McGhee, von Hippel formalism) [73] (Table 3). The obtained results showed a high affinity and negligible selectivity of **1** and **2** toward any of ds-DNA/RNA.

Consequently, fluorescence titrations point out that studied dipeptides **1** or **2** rapidly and strongly noncovalently bind to any ds-DNA or ds-RNA sequence in the tested sample, signaling interaction by change in fluorescence. Such a fast response allows further localized irradiation of the sample, triggering photochemical reaction inside DNA or RNA.

#### 2.4.3. CD Titrations

To investigate the mode of peptides **1** and **2** binding to polynucleotides, circular dichroism (CD) spectroscopy was used. Namely, the binding of small molecules to chiral macromolecules such as DNA provides distinctive different spectral responses for intercalators and groove-binding derivatives [74,75,76]. The studied **1** and **2** are chiral but the chromophore (pyrene) is not attached close to the chiral center, thus CD spectra of the studied compounds within the 220–400 nm range are negligible under the used experimental conditions (Figure S23 in the Supplementary Materials).

**Figure 4:**
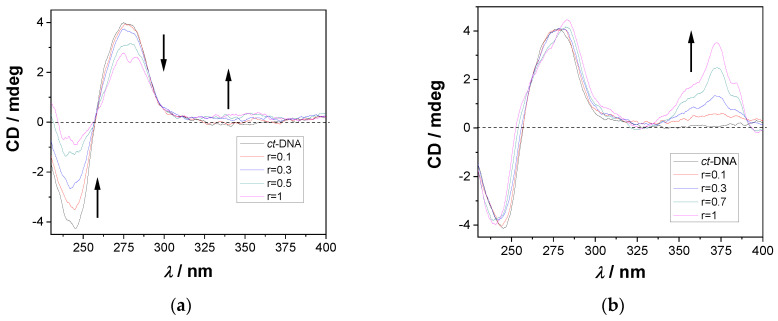
(**a**) CD titration of *ct*-DNA (*c* = 2 × 10^−5^ M) with different molar ratios r = [dipeptide]/[*ct*-DNA] for **1**; (**b**) CD titration of *ct*-DNA (*c* = 2 × 10^−5^ M) with different molar ratios r = [dipeptide]/[*ct*-DNA] for **2**. The measurements were performed in an aqueous cacodylate buffer (pH 7.0, 50 mM at 25 °C) with <0.1% DMSO. The arrows indicate the tendency of spectral change during the titration.

Addition of dipeptides **1** or **2** to the solution of *ct*-DNA affected the CD bands of DNA in the *λ* 230–300 nm range differently (Figure 4); the more rigid **1** induced much more pronounced changes than flexible **2**. The positive ICD band in the 300–350 nm range agrees well with pyrene absorption and indicates the binding of peptides **1** or **2** into the minor groove of the helical ds-DNA structure. However, the intensity of a positive induced CD signal was much stronger for flexible **2** compared to **1**, suggesting that the pyrene in peptide **2** can adapt to ideal positioning within the DNA minor groove in respect to the DNA chiral axis. Obtained results suggest that the binding of smaller and more rigid peptide **1** into the DNA minor groove disturbs the helical structure of *ct*-DNA much more in comparison to its more flexible analogue **2**.

The CD titrations with the synthetic polynucleotides p(dAdT)_2_, p(dGdC)_2_ and pApU (Figures S24–S26 in the Supplementary Materials) demonstrated the sensitivity of the CD response to the base pair composition of the polynucleotide. Similar to the *ct*-DNA, the addition of peptide **2** to the p(dAdT)_2_ induced a CD signal at 325–375 nm, whereas for **1** this effect was negligible (Figure S24). Both peptides affected the CD signal of p(dAdT)_2_; however at variance to the *ct*-DNA, the impact of the flexible **2** was more pronounced than that of the rigid **1**. Adversely to p(dAdT)_2_, the addition of peptides to the p(dGdC)_2_ (Figure S25 in the Supplementary Materials) did not induce CD signals > 300 nm, which can be attributed to the amino groups of guanines protruding inside the GC-DNA minor groove, in that way sterically hindering deep and well-oriented insertion of the pyrene. Again, only the flexible **2** distorted the CD bands of the polynucleotide. Intriguingly, addition of **1** or **2** to the AU-RNA did not yield an induced CD band > 300 nm (Figure S26 in the Supplementary Materials), which can be attributed to the very broad and shallow minor groove, not supporting the well-defined and unified orientation of the pyrene in respect to the chiral axis [75]. The rigid peptide **1** also did not change chirality of the AU-RNA, whereas the more flexible **2** caused a decrease in the CD bands in the 220–300 range, pointing to a partial loss of chirality, likely due to distortion of the ds-RNA double helix.

These CD results indicate that **1** and **2** bind to the minor groove of the ds-DNA, differing in CD response between the AT regions and GC regions, as well as within grooves of ds-RNA. Particularly flexible **2** showed specific ICD bands for ds-DNA containing AT-sequences, as a result of the well-positioned pyrene in respect to the DNA chiral axis. Moreover, for well-defined synthetic polynucleotides, the flexible **2** induced more pronounced changes in the helical structure of DNA/RNA compared to the rigid **1**. However, fluorescence titrations point to a similar affinity of **1** or **2** toward all ds-DNA/RNA (Table 2), thus differences in the rigidity of **1** or **2**, as well as structural differences of DNA/RNA binding sites, do not play a significant role in the overall stability of the complexes formed.

### 2.5. Covalent Binding to Polynucleotides

The flexible peptide **2** showed a stronger impact on the helical structure of DNA/RNA in the CD experiments, and strong ICD bands for the AT-containing sequences, supporting deep insertion of the pyrene into the groove, which would bring the rest of the peptide in very close proximity to DNA. Therefore, we also investigated its photo-induced in situ formation of QM intermediates followed by eventual covalent reaction with DNA. This was assayed by irradiation of **2** (300 nm) in the presence of ds-model oligonucleotides, dA_10_–dT_10_ (still double stranded at 20 °C), whereby the efficiency of covalent conjugation could be analyzed by HPLC. The ds-helices of the oligonucleotides were obtained by annealing complementary single-stranded (ss) oligonucleotides (dA_10_ with dT_10_,), by standard protocols and the formation of ds-DNA was confirmed by CD spectroscopy (Figure S27 in the Supplementary Materials).

**Figure 5:**
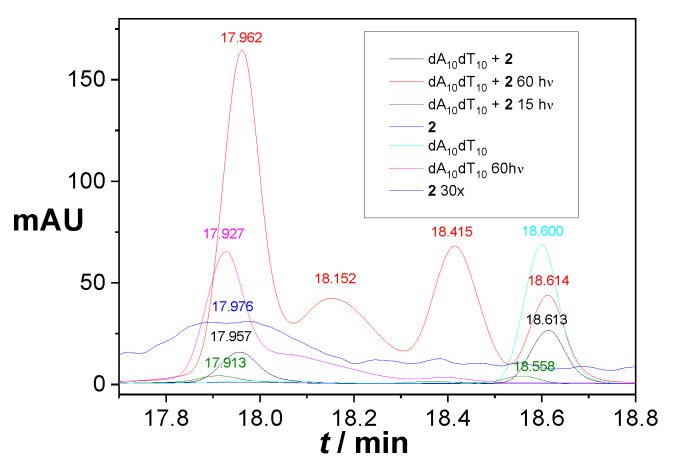
HPLC chromatogram detected at 260 nm (extract), peak analysis: blue line **2** (small peak at 17.976 min); blue line **2** 30×: (multiplied by 30 for better peak visualization); cyan (light blue line) dA_10_dT_10_ (18.600 min); gray line dA_10_dT_10_ + **2** (17.957 min, 18.613 min); purple line dA_10_dT_10_ *hν* (300 nm) = 60 min (17.927 min); green line dA_10_dT_10_ + **2** *hν* (300 nm) = 15 min (17.913 min, 18.558 min); red line +**2** *hν* (300 nm) = 60 min (17.962 min, 18.152 min, 18.415 min, 18.614 min).

The chromatogram of the ds–dA_10_–dT_10_ shows up as one peak with a retention time of 18.60 min and **2** as a signal with a retention time of 17.98 min. The addition of **2** to the solutions of ds-oligonucleotides and the formation of the complex did not affect their retention times on HPLC because noncovalent bonds dissociate under the HPLC chromatography conditions. After the irradiation of the **2·**DNA mixtures (300 nm, 15 and 60 min), the composition was also analyzed by HPLC, in parallel with two control experiments (irradiation of **2** under the same conditions, irradiation of ds-oligonucleotide). HPLC chromatograms after the irradiation of the mixture **2**·ds–dA_10_–dT_10_ show two new peaks (18.15 min and 18.42 min), which did not appear in either **2** or ds–dA_10_–dT_10_, after the irradiation. Consequently, the new peaks in the chromatograms can be attributed to the photoinduced alkylation of the oligonucleotides (Figure 5, Figures S28 and S29 in the Supplementary Materials), likely differing in the number of attached molecules **2** (one or two) to ds-DNA. Note that the current photo-alkylation study of a ds-oligonucleotide by HPLC analysis could not reveal the identity of the alkylation photoproduct. However, based on work by Rokita et al., it is very likely that the exocyclic adenine NH_2_ at position 6 is alkylated [42].

## 3. Materials and Methods

### 3.1. General Procedure for the Preparation of Pyrene Amino Acids **4** and **5**

(*S*)-3-amino-2-((*tert*-butoxycarbonyl)amino)propanoic acid or (*tert-*butoxycarbonyl)-l-lysine (1 eq.) and KHCO_3_ (1.1 eq.) were dissolved in dry DMF (15 mL) under nitrogen at rt. A solution of 2,5-dioxopyrrolidin-1-yl pyrene-1-carboxylate (1 eq.) in dry DMF (7 mL) was added dropwise and the reaction mixture was stirred at rt 72 h. The solvent was evaporated. To the residue, ethyl acetate (30 mL) and 10% aqueous citric acid (30 mL) were added and the product was extracted with ethyl acetate (3 × 30 mL). The organic extracts were dried over anhydrous Na_2_SO_4_, filtered and the solvent was removed on a rotational evaporator. The product was purified on a column of silica gel by use of 15% CH_3_OH in CH_2_Cl_2_ as eluent.

#### 3.1.1. (*S*)-2-((Tert-butoxycarbonyl)amino)-3-(pyrene-1-carboxamido)propanoic Acid (**4**)

The compound was prepared from (*S*)-3-amino-2-((*tert*-butoxycarbonyl)amino)propanoic acid (0.291 mmol, 59 mg) and KHCO_3_ (0.320 mmol, 32 mg) in dry DMF (15 mL), in the presence of 2,5-dioxopyrrolidin-1-yl pyrene-1-carboxylate (**3**, 0.291 mmol, 100 mg) in dry DMF (7 mL). After chromatographic purification, the reaction afforded pure product (80 mg, 0.185 mmol, 64%) in the form of colorless oil.

^1^H NMR (300 MHz, CDCl_3_): *δ* = 8.22–7.54 (m, 9H), 6.20 (s, 1H), 4.50 (s, 1H), 3.93 (s, 1H), 1.37 (s, 9H); ^13^C NMR (151 MHz, CDCl_3_): *δ* = 171.2, 130.5, 128.7, 126.9, 125.8, 124.8, 124.1, 80.9, 28.5; HRMS: C_25_H_24_N_2_O_5_ ([M + H]^+^) found 433.1703, calculated: 433.1765.

#### 3.1.2. (*S*)-2-((Tert-butoxycarbonyl)amino)-6-(pyrene-1-carboxamido)hexanoic Acid (**5**)

The compound was prepared from (*S*)-6-amino-2-((tert-butoxycarbonyl)amino)hexanoic acid (72 mg, 0.291 mmol) and KHCO_3_ (32 mg, 0.320 mmol) in dry DMF (15 mL), in the presence of 2,5-dioxopyrrolidin-1-yl pyrene-1-carboxylate (**3**, 100 mg, 0.291 mmol) in dry DMF (7 mL). After chromatographic purification, the reaction afforded pure product (113 mg, 0.238 mmol, 82%) in the form of colorless oil.

^1^H NMR (600 MHz, CD_3_OD): *δ* = 8.44 (d, *J* = 9.2 Hz, 1H), 8.28–8.17 (m, 5H), 8.13–8.06 (m, 3H), 4.18–4.12 (m, 1H), 3.57 (t, *J* = 7.0 Hz, 2H), 1.97–1.90 (m, 1H), 1.83–1.74 (m, 3H), 1.67–1.57 (m, 2H), 1.46–1.40 (m, 9H); ^13^C NMR (151 MHz, CD_3_OD): *δ* = 176.3, 158.2, 133.8, 132.8, 132.6, 132.1, 129.6, 128.2, 127.5, 126.9, 126.8, 125.9, 125.8, 125.6, 125.5, 125.4, 80.5, 54.9, 40.9, 32.6, 30.1, 28.7, 24.5; HRMS: C_28_H_30_N_2_O_5_ ([M + H]^+^) found 475.2221, calculated 475.2235 and ([M + Na]^+^) found 497.2046, calculated 497.2055.

### 3.2. General Procedure for the Preparation of Dipeptides **7** and **8**

(*S*)-2-((*tert*-butoxycarbonyl)amino)-3-(pyrene-1-carboxamido)propanoic acid (**4**) or (*S*)-2-((*tert*-butoxycarbonyl)amino)-6-(pyrene-1-carboxamido)hexanoic acid (**5**) (1 eq.) was dissolved in dry DMF (15 mL) under nitrogen at rt. HBTU (1.1 eq.), HOBt (1.1 eq.) and TEA (4 eq.) were added and the reaction mixture was stirred for 20 min. The solution of H-Tyr[CH_2_N(CH_3_)_2_]-OBn (**6**) and TEA (2 eq.) in dry DMF (5 mL) was added dropwise and the reaction mixture was stirred for 48 h. The solvent was evaporated. To the residue ethyl acetate (30 mL) and water (30 mL) were added and the product was extracted with ethyl acetate (3 × 30 mL). The organic extracts were dried over anhydrous Na_2_SO_4_, filtered and the solvent was removed on a rotational evaporator. The product was purified on a column of silica gel by use of 10% CH_3_OH in CH_2_Cl_2_ as eluent, followed by a preparative TLC on silica gel by use of 10% CH_3_OH in CH_2_Cl_2_ as eluent.

#### 3.2.1. (*S*)-Benzyl 2-((*S*)-2-((tert-butoxycarbonyl)amino)-3-(pyrene-1-carboxamido)propanamido)-3-(3-((dimethylamino)methyl)-4-hydroxyphenyl)propanoate (**7**)

The compound was prepared from (*S*)-2-((*tert*-butoxycarbonyl)amino)-3-(pyrene-1-carboxamido)propanoic acid (**4**, 80 mg, 0.185 mmol), HBTU (77 mg, 0.204 mmol), HOBt (27 mg, 0.204 mmol) and TEA (103 μL, 0.740 mmol) in dry DMF (15 mL), in the presence of H-Tyr[CH_2_N(CH_3_)_2_]-OBn) (**6**, 61 mg, 0.185 mmol) and TEA (52 μL, 0.371 mmol) in dry DMF (5 mL). After chromatographic purification, the reaction afforded pure product (31 mg, 0.042 mmol, 23%) in the form of pale yellow oil.

^1^H NMR (600 MHz, CDCl_3_): *δ* = 8.62–8.53 (m, 1H), 8.24–8.20 (m, 2H), 8.14–8.08 (m, 3H), 8.06–8.01 (m, 3H), 7.42–7.33 (m, 1H), 7.32–7.29 (m, 2H), 7.24–7.19 (m, 2H), 7.00 (s, 1H), 6.83–6.77 (m, 1H), 6.69–6.62 (m, 2H), 6.23–6.07 (m, 1H), 5.13–5.02 (m, 2H), 4.83–4.78 (m, 1H), 4.48–4.39 (m, 1H), 4.08–3.96 (m, 1H), 3.87–3.75 (m, 1H), 3.47–3.37 (m, 2H), 3.06–2.98 (m, 2H), 2.20–2.17 (m, 6H), 1.47–1.44 (m, 9H); ^13^C NMR (151 MHz, CDCl_3_): *δ* = 171.3, 170.5, 157.3, 135.2, 132.9, 131.3, 130.8, 129.9, 129.6, 129.3, 128.9, 128.7, 128.6, 128.5, 127.3, 126.5, 126.1, 125.9, 124.9, 124.5, 116.3, 80.7, 67.3, 62.6, 53.4, 44.5, 42.8, 37.2, 28.5; HRMS: C_44_H_46_N_4_O_7_ ([M + H]^+^) found 743.3447, calculated 743.3446 and ([M + Na]^+^) found 765.3262, calculated 765.3266.

#### 3.2.2. (*S*)-Benzyl 2-((*S*)-2-((tert-butoxycarbonyl)amino)-6-(pyrene-1-carboxamido)hexanamido)-3-(3-((dimethylamino)methyl)-4-hydroxyphenyl)propanoate (**8**)

The compound was prepared from (*S*)-2-((*tert*-butoxycarbonyl)amino)-6-(pyrene-1-carboxamido)hexanoic acid (**5**, 113 mg, 0.238 mmol), HBTU (99 mg, 0.262 mmol), HOBt (35 mg, 0.262 mmol) and TEA (133 μL, 0.952 mmol) in dry DMF (15 mL), in the presence of H-Tyr[CH_2_N(CH_3_)_2_]-OBn) (**6**, 78 mg, 0.298 mmol) and TEA (67 μL, 0.476 mmol,) in dry DMF (5 mL). After chromatographic purification, the reaction afforded pure product (0.087 mmol, 68 mg, 37%) in the form of pale yellow oil.

^1^H NMR (300 MHz, CDCl_3_): *δ* = 8.54 (d, *J* = 9.3 Hz, 1H), 8.24–8.18 (m, 2H), 8.16–8.12 (m, 1H), 8.11–7.99 (m, 5H), 7.28–7.27 (m, 1H), 7.15–7.07 (m, 2H), 6.77–6.70 (m, 1H), 6.65–6.58 (m, 2H), 6.53–6.40 (m, 2H), 5.23–5.10 (m, 1H), 4.88–4.71 (m, 3H), 3.67–3.51 (m, 2H), 3.47–3.38 (m, 2H), 2.97–2.87 (m, 2H), 2.22 (s, 6H), 1.93–1.81 (m, 1H), 1.77–1.61 (m, 3H), 1.55–1.44 (m, 2H), 1.37 (s, 9H); ^13^C NMR (151 MHz, CDCl_3_): *δ* = 171.9, 171.5, 170.4, 157.2, 135.0, 132.5, 131.3, 130.8, 129.7, 129.5, 128.8, 128.7, 128.6, 128.5, 128.4, 127.2, 126.4, 125.9, 125.8, 124.8, 124.6, 124.5, 124.4, 116.3, 80.1, 67.1, 62.3, 54.3, 53.5, 44.4, 39.7, 36.9, 29.8, 29.2, 28.4, 22.6; HRMS: C_47_H_52_N_4_O_7_ ([M + H]^+^) found 785.391, calculated 785.388.

### 3.3. General Procedure for the Removal of the BOC Protective Group from **7** and **8**

(*S*)-benzyl 2-((*S*)-2-((*tert*-butoxycarbonyl)amino)-3-(pyrene-1-carboxamido)propanamido)-3-(3-((dimethylamino)methyl)-4-hydroxyphenyl)propanoate (**7**) or (*S*)-benzyl 2-((*S*)-2-((*tert*-butoxycarbonyl)amino)-6-(pyrene-1-carboxamido)hexanamido)-3-(3-((dimethylamino)methyl)-4-hydroxyphenyl)propanoate (**8**) was dissolved in CH_2_Cl_2_ (0.5–1.5 mL) and TFA (0.5–1 mL) was added. The reaction mixture was stirred for 1h, solvent was evaporated, with addition of toluene, and the residue was purified by semipreparative HPLC (see the Supplementary Materials). 

### 3.4. Preparative Irradiations in CH_3_OH

(*S*)-benzyl 2-((*S*)-2-((*tert*-butoxycarbonyl)amino)-3-(pyrene-1-carboxamido)propanamido)-3-(3-((dimethylamino)methyl)-4-hydroxyphenyl)propanoate (**7**) or (*S*)-benzyl 2-((*S*)-2-((*tert*-butoxycarbonyl)amino)-6-(pyrene-1-carboxamido)hexanamido)-3-(3-((dimethylamino)methyl)-4-hydroxyphenyl)propanoate (**8**) were dissolved in CH_3_OH (15 mL). The solution was purged with Ar for 15 min, sealed and irradiated in a Luzchem reactor equipped with 8 lamps (cool white, 300 nm, each lamp 8W). The composition of the irradiated mixture was followed by HPLC. The solvent was removed on a rotary evaporator and the photoproduct was purified on preparative liquid chromatography plates by use of 10% CH_3_OH in CH_2_Cl_2_ as eluent.

#### 3.4.1. (*S*)-Benzyl 2-((*S*)-2-((tert-butoxycarbonyl)amino)-3-(pyrene-1-carboxamido)propanamido)-3-(4-hydroxy-3-(methoxymethyl)phenyl)propanoate (**7-OCH_3_**)

The compound was prepared from the two same solution (*S*)-benzyl 2-((*S*)-2-((*tert*-butoxycarbonyl)amino)-3-(pyrene-1-carboxamido)propanamido)-3-(3-((dimethylamino)methyl)-4-hydroxyphenyl)propanoate (50 mg, 0.073 mmol) dissolved in CH_3_OH (15 mL) in two quartz tubes. The product (21 mg, 0.021 mmol, 21%) was isolated in the form of colorless oil while the starting compound 7 was recovered (3 mg, 7%).

^1^H NMR (600 MHz, CDCl_3_): *δ* = 8.59–8.52 (m, 1H), 8.23–8.19 (m, 2H), 8.13–8.09 (m, 2H), 8.08–8.01 (m, 4H), 7.41–7.33 (m, 2H), 7.31–7.30 (m, 2H), 7.25–7.22 (m, 2H), 7.01 (s, 1H), 6.83 (d, *J* = 7.6 Hz, m,1H), 6.72–6.64 (m, 2H), 6.22–6.09 (m, 1H), 5.11 (d, *J* = 12.1Hz, 1H), 5.05 (d, *J* = 12.2 Hz, 1H), 4.83–4.79 (m, 1H), 4.48–4.38 (m, 3H), 4.04–3.94 (m, 1H), 3.87–3.81 (m, 1H), 3.35–3.24 (m, 3H), 3.06–2.97 (m, 2H), 1.45 (s, 9H); ^13^C NMR (151 MHz, CDCl_3_): *δ* = 171.2, 170.6, 155.3, 135.2, 132.9, 131.3, 130.8, 129.9, 128.9, 128.7, 128.6, 127.2, 126.5, 126.1, 125.9, 124.8, 124.4, 116.7, 73.8, 67.4, 58.3, 53.9, 37.1, 29.8, 28.4; HRMS: C_43_H_43_N_3_O_8_ ([M + Na]^+^) found 752.2957, calculated 752.2950.

#### 3.4.2. (*S*)-Benzyl 2-((*S*)-2-((tert-butoxycarbonyl)amino)-6-(pyrene-1-carboxamido)hexanamido)-3-(4-hydroxy-3-(methoxymethyl)phenyl)propanoate (**8-OCH_3_**)

The compound was prepared from the four same solutions of (*S*)-benzyl 2-((*S*)-2-((*tert*-butoxycarbonyl)amino)-6-(pyrene-1-carboxamido)hexanamido)-3-(3-((dimethylamino)methyl)-4-hydroxyphenyl)propanoate (7 mg, 0.009 mmol) dissolved in CH_3_OH (15 mL) in four quartz tubes. The product (9 mg, 0.012 mmol, 33%) was isolated in the form of colorless oil while the starting compound **8** was recovered (4 mg, 14%).

^1^H NMR (600 MHz, CD_3_OD): *δ* = 8,44 (d, *J* = 9.2 Hz, 1H), 8.25 (d, *J* = 7.8 Hz, 2H), 8.23–8.19 (m, 2H), 8.15 (d, *J* = 8.9 Hz, 1H), 8.10 (d, *J* = 8.0 Hz, 2H), 8.06 (t, *J* = 7.6 Hz, 1H), 7.30–7.25 (m, 3H), 7.18–7.09 (m, 2H), 7.02 (s, 1H), 6.89–6.81 (m, 1H), 6.65 (d, *J* = 8.2 Hz, 1H), 4.96–4.87 (m, 2H), 4.65 (t, *J* = 6.8 Hz, 1H), 4.46–4.36 (m, 2H), 4.10–3.97 (m, 1H), 3.56–3.51 (m, 2H), 3.34–3.32 (m, 3H), 3.04–2.92 (m, 2H), 1.79–1.69 (m, 3H), 1.66–1.60 (m, 1H), 1.56–1.47 (m, 1H), 1.39 (s, 9H); ^13^C NMR (151 MHz, CD_3_OD): *δ* = 174.9, 172.9, 172.8, 157.8, 155.7, 136.8, 133.8, 132.8, 132.6, 132.1, 131.6, 130.8, 129.7, 129.6, 129.5, 129.3, 128.2, 127.6, 126.9, 126.8, 125.9, 125.8, 125.6, 125.5, 125.4, 116.2, 80.6, 70.9, 67.9, 58.4, 55.9, 55.4, 40.9, 37.7, 33.2, 30.1, 28.7, 24.3; HRMS: C_46_H_49_N_3_O_8_ ([M + Na]^+^) found 794.344, calculated 794.340.

## 4. Conclusions

Two dipeptides **1** and **2** were synthesized that were composed of unnatural amino acids containing a pyrene as a fluorescent label and polynucleotide binding unit, and the modified tyrosine as a photochemically reactive unit. Peptides **1** and **2** are fluorescent (*Φ*_f_ = 0.3–0.4, the emission attributed to the pyrene) and undergo photochemical deamination reaction on the modified tyrosine (*Φ*_R_ = 0.01–0.02), that can lead to the alkylation of polynucleotides. The peptides bind by strong noncovalent interactions (log *K*_a_ > 6) to minor grooves of polynucleotides, preferably to the AT reach regions. The differences between **1** and **2** in the length and flexibility of the linker between the pyrene and the modified tyrosine did not significantly affect interactions with ds-DNA or ds-RNA. The peptide derivative **2,** with a longer spacer between the pyrene and the modified tyrosine, undergoes a more efficient photodeamination reaction, based on the comparison with the *N*-Boc protected derivatives. Upon irradiation of the noncovalent complex **2**·oligoAT_10_, the photo-generation of QMs initiates alkylation of the oligonucleotide, thus covalently linking **2** to the oligoAT_10_. This study demonstrated, as proof of principle, that small molecules can combine the dual form of fluorescent labeling of polynucleotides. Namely, the initial addition of a dye rapidly forms a reversible high-affinity noncovalent complex with a ds-DNA/RNA, which can upon irradiation by light be converted to the irreversible (covalent) form. Such reversible/irreversible dyes can have numerous applications in biomedicinal sciences, based on the staining of all DNA/RNA in a sample but fixing the dye to the polynucleotide only in the irradiated spots, whereas the dye can be washed off the rest of the sample.

## Figures and Tables

**Table 1:** Quantum yields of fluorescence (*Φ*_f_) and fluorescence decay times (*τ*_f_) for compounds **5**, **7** and **8** in CH_3_CN.

Compound	*Φ*_f_^1^	*τ*_f_ (341 nm) ^2^/ns	*τ*_f_ (281 nm) ^2^/ns
**7**	0.4 ± 0.1	0.5 ± 0.1 (0.02) 7.02 ± 0.06 (0.71) 27.1 ± 0.5 (0.27)	0.37 ± 0.05 (0.03) 7.05 ± 0.06 (0.70) 26.4 ± 0.4 (0.27)
**8**	0.3 ± 0.1	0.10 ± 0.05 (0.01) 7.7 ± 0.1 (0.85) 15.8 ± 0.9 (0.14)	0.06 ± 0.04 (0.02) 7.77 ± 0.09 (0.84) 16.6 ± 0.9 (0.14)
**5**	0.5 ± 0.1	5 ± 1 (0.01) 25.87 ± 0.07 (0.99)	

^1^ Quantum yield of fluorescence was measured using quinine sulfate as actinometer in H_2_O/H_2_SO_4_ (*Φ*_f_ = 0.546) [63]. The fluorescence spectra were measured by exciting samples at 310, 320 and 330 nm, and the average values were calculated (eq. in the Supplementary Materials). The associated errors correspond to the maximum absolute deviation. ^2^ Decay times were measured by TC-SPC. Fluorescence decays were obtained by exciting samples at 281 and 341 nm and detecting fluorescence at 400 nm. The relative contribution of decay components is given in parenthesis. The associated errors correspond to maximal standard deviations obtained from the fitting.

**Table 2:** Quantum yield for the photomethanolysis reaction (*Φ*_R_) of model dipeptides **7** and **8** upon excitation at 254 nm ^1^.

Compound	*Φ*_R_/(10^−2^)
**7**	1.33 ± 0.03
**8**	2.56 ± 0.05

^1^ Determined by use of a KI/KIO_3_ actinometer (*Φ*_254_ = 0.74) [63,66]. The irradiation was conducted in a Luzchem reactor at 254 nm, using one lamp (8 W). The composition of the solution was determined by HPLC (see Table S5 in the Supplementary Materials).

**Table 3:** Binding constants (log*K*_a_) of **1** and **2** with ds-DNA/ds-RNA determined by fluorescence titrations ^1^.

	1	2
*ct*-DNA	5.6	6.0
pApU	6.8	6.9
p(dAdT)_2_	>7	6.8
p(dGdC)_2_	6.7	6.6

^1^ The measurement was performed in a sodium cacodylate buffer (pH = 7.0, 50 mM, at 25 °C). The processing of the titration data by means of the Scatchard equation gave values of ratio *n*[bound dye]/[DNA/RNA] = 0.2 and 0.5, for easier comparison all log*K* values were re-calculated for fixed *n* = 0.3.

## Data Availability

Data from this article is available on request from authors and is openly available in Public Documents_HrZZ-IP-2019-04-8008 at: https://mojoblak.irb.hr/s/PXPWDXZ2QCp3daC, accessed on 9 November 2023.

## Supplementary Materials

The following supporting materials can be downloaded at: https://www.mdpi.com/article/10.3390/molecules28227533/s1, including general experimental procedures, synthetic procedures for the preparation of known compounds, photophysical measurements, photochemical reactivity, noncovalent binding to polynucleotides, covalent binding to polynucleotides and copies of ^1^H and ^13^C NMR spectra. Refs. [77,78,79,80] are cited in the Supplementary Materials.
