# Supplementary material for: Dipeptides Containing Pyrene and Modified Photochemically Reactive Tyrosine: Noncovalent and Covalent Binding to Polynucleotides
Source: Molecules. 2023 Nov 10;28(22):7533. doi: 10.3390/molecules28227533 (PMC10672942; doi:10.3390/molecules28227533)
Supplement: Supplementary file 1 [file molecules-28-07533-s001.zip › molecules-2674186-supplementary.pdf]

Supplementary Materials

for

Dipeptides containing pyrene and modified photochemically reactive tyrosine: non-covalent and covalent binding to polynucleotides

Igor Sviben,[a] Mladena Glavaš,[a] Antonija Erben,[a] Thomas Bachelart,[a,b] Dijana Pavlović Saftić,[a] Ivo Piantanida,[a]* Nikola Basarić[a]*

[a] Department of Organic Chemistry and Biochemistry, Ruđer Bošković Institute, Bijenička cesta 54, 10 000 Zagreb, Croatia

[b] Current address: Team of Biosystems Chemistry; UMR 7242 Biotechnology and Cellular Signalling; Ecole supérieure de biotechnologie de Strasbourg (ESBS); University of Strasbourg; 300 Bd Sébastien Brant, 67400 Illkirch-Graffenstaden, France

Correspondence to: IP ivo.piantanida@irb.hr; NB nikola.basaric@irb.hr

Content:

1. Synthetic procedures for the preparation of known compounds and unsuccessful attempts to prepare pyrene-alanine dipeptide (Scheme S1 and S2 and Tables S1-S4) S2

2. Photophysical measurements (Figs S1-S13, Eqs S1 and S2) S8

3. Photochemical reactivity (Eqs S3-S7 and Table S5) S14

4. Steric properties of studied DNA and RNA (Table S6) S16

4. Non-covalent binding to polynucleotides (Figs S12-S25 and Table S7) S17

5. Covalent binding to polynucleotides (Figs S27-S29 and Table S8) S24

6. NMR spectra and HRMS S27

7. References S49

<u>1. General experimental procedures, synthetic procedures for the preparation of known compounds and unsuccessful attempts to prepare pyrene-alanine dipeptide</u>

General experimental procedures

All chemicals and solvents used were analytical or HPLC grade and commercially available. Reactions were monitored using thin layer chromatography (TLC) on silica gel plates (Silica gel 60 F_{254}, Merck, Germany) and by reverse-phase high performance liquid chromatography (RP-HPLC) on HPLC Agilent 1260Infinity II with DAD detector. The HPLC was performed on an analytical column Phenomenex LC 150 × 4.6 mm, LUNA 3µm C18(2) 100Å. For detection was used method: linear gradient from 100% phase B to 100% phase A for 30 min, flow rate 0.8 mL/min, monitored at 254 nm and 280 nm. The solvent system was methanol (A) and 0.1% TFA in methanol/water (1:1; B). The compounds were detected on TLC plates under UV light at 254 nm and 366 nm and on HPLC. The products were purified on silica gel column chromatography on silica gel 60 (0.040 – 0.063 mm) (Milipore, Germany) and on preparative TLC (PLC; Silica gel 60 F254, 1mm and 2 mm, Merck, Germany). Chemicals were purchased from the usual commercial sources and were used as received. Solvents for chromatographic separations were used as they are delivered from supplier (p.a. or HPLC grade) or purified by distillation (CH_2Cl_2, hexane). Determination and characterization of product structure was carried out by nuclear magnetic resonance (NMR) and high resolution mass spectrometry (HRMS). The NMR spectra were recorded on Bruker Avance III HD 600 MHz/54mm Ascend equipped with 5mm three-channel inversion TCI cryo-probe Prodigy with Z-gradient spiral (model CPP1.1 TCI600S3 H&F-C/N-D-05 Z XT) under the frequency of 300 MHz and 600 MHz (^{1}H) and 75 MHz and 151 MHz (^{13}C) in CD_3OD or $CDCl_3$ at rt. The spectra were processed in the program MestReNova version 6.0.2-5475, Mestrelab Research S,L; 2009 and analyzed according to one dimensional (^{1}H and ^{13}C) spectra. Chemical shifts (δ) are expressed according to deuterium solvent. High resolution mass spectrometry was recorded on two different instruments. The first instrument was a MALDI TOF/TOF 4800 Plus Analyzer operating in reflection mode. Calibrant and analyte spectra were obtained in positive and negative ion mode. Calibration type was internal with the calibrant produced by matrix ionization (monomeric, dimeric and trimeric CHCA), with azithromycin and angiotensisn II dissolved in α-cyano-4-hydroxycinnamic acid matrix. Accurately measured spectra were internally calibrated and elemental analysis was performed on the Data Explore v. 4.9 software with a mass accuracy better than 5 ppm. The second instrument was Agilent 6550 Series Accurate-Mass-Quadrupole Time-of-Flight (Q-TOF), and the used column was Zorbax Eclipse Plus C18, 3.0 × 50 mm, 1.8 µm. Compound names were generated by ChemDraw Professional (version 20.0.0.41) which follows the IUPAC conventions.

Semipreparative HPLC separations were performed on a Varian Pro Star instrument equipped with a Phenomenex Jupiter C18 5µ 300A column, using CH_3OH/H_2O + TFA as an eluent. Analysis of samples was performed on an Agilent 1260 Infinity II HPLC equipped with Phenomenex Luna 3u C18(2) column and diode-array detector (eluent: CH_3OH/H_2O + TFA). Analysis of the solutions containing annealed oligonucleotides and **2** were performed on Agilent 1260 Infinity II HPLC equipped with a PLRP-S 5 µm column and the mobile phase

was CH_3CN/ammonium acetate aqueous buffer (100 mM, pH 7.0). Details on methods for the sample analysis are given in the SI.

Irradiation experiments were performed in a Luzchem reactor equipped with 1-8 lamps (8 W/lamp) with the output at 254 and 300 nm. Solvents for the irradiations were of HPLC purity.

Polynucleotides were purchased as noted: poly A-poly U, poly $(dGdC)_2$, poly $(dAdT)_2$, *ct*-DNA (Sigma). Polynucleotides were dissolved in Na-cacodylate buffer, $I = 0.05$ M, pH 7.0. *ct*-DNA was additionally sonicated and filtered through a 0.45 mm filter. Polynucleotide concentration was determined spectroscopically as the concentration of phosphates (nucleobases), as described by producer.

2,5-dioxopyrrolidin-1-yl pyrene-1-carboxylate (3) [1,2]

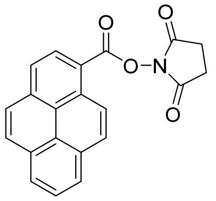

In a flask (100 mL), pyrene carboxylic acid (200 mg, 0.812 mmol), hydroxy-succinimide (94 mg, 0.812 mmol) and EDC×HCl (*N*-(3-Dimethylaminopropyl)-*N'*-ethylcarbodiimide hydrochloride) (172 mg, 0.994 mmol) were dissolved in dry CH_2Cl_2 (40 mL). The reaction mixture was stirred under N_2 inert atmosphere for 3h at -5 °C, and then over night at rt. The reaction mixture was washed with a solution of Na_2CO_3 (0.5 M, 20 mL), H_2O (20 mL), solution of HCl (0.5 M, 20 mL), and H_2O (20 mL). The organic layer was dried over anhydrous $MgSO_4$, filtered and the solvent was removed on a rotary evaporator. The residue in the form of yellow solid was obtained (220 mg, 79%) that was used in the next step without purification.
^{1}H NMR (300 MHz, DMSO-d$_6$): δ = 8.92 (d, 1H, J = 9.4 Hz), 8.70 (d, 1H, J = 8.2 Hz), 8.48-8.37 (m, 5H), 8.26 (d, 1H, J = 9.0 Hz), 8.19 (t, 1H, J = 7.6 Hz), 3.00 (s, 4H).

(*S*)-benzyl 2-((*tert*-butoxycarbonyl)amino)-3-(3-((dimethylamino)methyl)-4-hydroxyphenyl)propanoate [3]

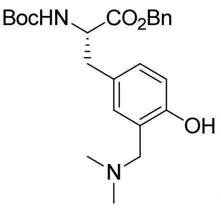

(*S*)-benzyl 2-((*tert*-butoxycarbonyl)amino)-3-(4-hydroxyphenyl)propanoate (Boc-Tyr-OBn; 1.03 g, 2.77 mmol), K_2CO_3 (421 mg, 3.05 mmol) and Eschenmoser's salt (285 mg, 3.05 mmol) were suspended in dry DCM (130 mL) under nitrogen inert atmosphere at rt. The reaction mixture was stirred 72h and the solvent was removed on a rotational evaporator. The product was purified on a column of aluminum oxide (activity III) using 3% MeOH in CH_2Cl_2 as eluent to obtained the pure product as yellowish oil (1.13 g, 2.64 mmol, 95%).

^{1}H NMR (300 MHz, CD_3OD): δ = 7.37 – 7.24 (m, 5H), 6.92 (dd, J = 6.9 Hz, J = 1.3 Hz, 1H), 6.84 (d, J = 1.3 Hz, 1H), 6.64 (d, J = 6.9 Hz, 1H), 5.12 (d, J = 12.4 Hz, 1H), 5.06 (d, J = 12.4 Hz, 1H), 4.32 (dd, J = 6.4 Hz, J = 8.4 Hz, 1H), 3.54 (s, 2H), 2.96 (dd, J = 6.4 Hz, J = 13.6 Hz, 1H), 2.82 (dd, J = 8.4 Hz, J = 13.6 Hz, 1H), 2.26 (s, 6H), 1.38 (br s, 9H); ^{13}C NMR (75 MHz, CD_3OD): δ = 173.7, 131.1, 130.5, 129.5, 129.3, 128.6, 123.6, 116.6, 80.6, 67.8, 62.4, 56.9, 44.7, 37.9, 26.7.

Prior to the peptide coupling reactions compound **6** was prepared by a removal of the BOC group by use of TFA/DCM. (*S*)-benzyl 2-((*tert*-butoxycarbonyl)amino)-3-(3-((dimethylamino)methyl)-4-hydroxyphenyl)propanoate was dissolved in CH_2Cl_2 (2 mL) and TFA (2 mL) was added. The reaction mixture was stirred 1h, the solvent was evaporated, with addition of toluene, and the residue was used in the next step.

Attempted preparation of benzyl (*S*)-2-((*S*)-2-((*tert*-butoxycarbonyl)amino)propanamido)-3-(3-((dimethylamino)methyl)-4-hydroxyphenyl)propanoate (B)

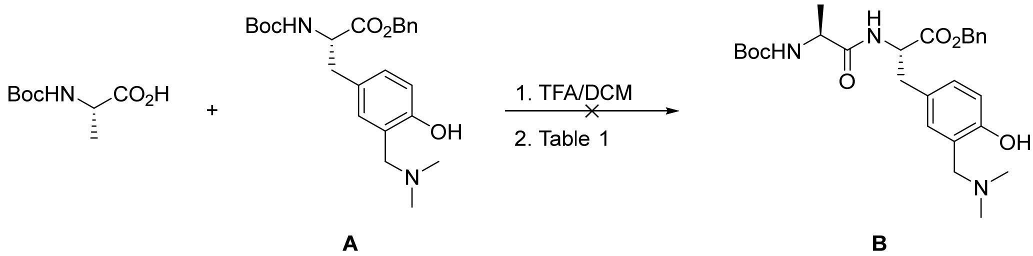

Scheme S1. Attempted synthesis of (*S*)-2-((*S*)-2-((tert-butoxycarbonyl)amino)propanamido)-3-(3-((dimethylamino)methyl)-4-hydroxyphenyl)propanoate (**B**)

Table S1. Optimization of reaction conditions for the synthesis of product **B**.

Boc-Ala/eq.[a]	**A**/eq.	Condensation reagent/eq.	Base/eq.	Solvent	Yield/%[i]
1	1	HATU[b]/1.1	NMM/2.2	DMF	-
1	1	HATU/1.1	NMM/2.2	DCM	-
1	1	TBTU[c]/1.1	DIPEA/2.2	DCM	-
1	1	IBCF[d]/1.1	NMM/2.2	DCM	-
1	1	NHS/EDC[e] 1/1.2	$NaHCO_3$ or $KHCO_3$/3	DCM	-
1	1	EDC/HOBt 1.1/1.1	$NaHCO_3$/1.5	DCM/20% DMF	-
1	1	BOP[f]/HOBt[g] 1.2/1.2	NMM/1.7	DCM	-
1	1	PyBOP[h]/1	NMM/3.7	DCM	-

[a]All reactions were performed under the room temperature; [b]1-[Bis(dimethylamino)methylene]-1*H*-1,2,3-triazolo[4,5-*b*]pyridinium 3-oxid hexafluorophosphate; [c]2-(1H-Benzotriazole-1-yl)-1,1,3,3-tetramethylaminium tetrafluoroborate; [d]Isobutyl chloroformate. Reaction was performed at 0 °C; [e]1-Ethyl-3-(3-

dimethylaminopropyl)carbodiimide; [f]Benzotriazol-1-yloxytris(dimethylamino)phosphonium hexafluorophosphat; [g]1-Hydroxybenzotriazole hydrate, [h]Benzotriazole-1-yl-oxy-tris-pyrrolidino-phosphonium hexafluorophosphate; [i]The product was not obtained.

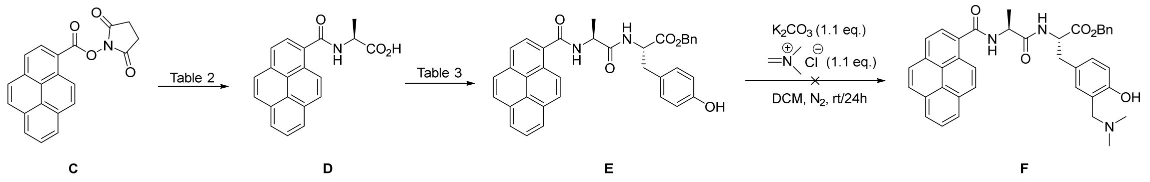

Scheme S2. Synthesis of benzyl (*S*)-3-(3-((dimethylamino)methyl)-4-hydroxyphenyl)-2-((*S*)-2-(pyrene-1-carboxamido)propanamido)propanoate (**F**)

(Pyrene-1-carbonyl)-L-alanine (D)

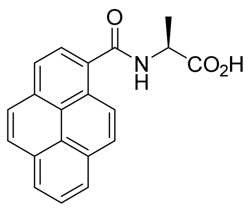

L-alanine (168 mg, 1.89 mmol) and $KHCO_3$ (189 mg, 1.89 mmol) were dissolved in dry DMF (16 mL) under nitrogen inert atmosphere at rt. In another flask, 2,5-dioxopyrrolidin-1-yl pyrene-1-carboxylate (**3**, 590 mg, 1.72 mmol) was dissolved in dry DMF (5 mL) under nitrogen inert atmosphere and was added dropwise over 1h. The reaction mixture was stirred for 5 days and the solvent was evaporated on a rotational evaporator. To the residue, 1M HCl (~10 mL) and ethyl acetate (~20 mL) were added and the product was extracted with ethyl acetate (3×30 mL). The organic extracts were dried over anhydrous Na_2SO_4, filtered and the solvent was removed on a rotational evaporator. The product was purified on a column of silica gel by use of hexan:ethyl acetate:acetic acid = 2:1:0.5 as eluent to obtained the pure product as white oil (245 mg, 0.772 mmol, 45%).

^{1}H NMR (600 MHz, CH_3OH): δ = 8.58 (d, J = 9.2 Hz, 1H), 8.28 – 8.24 (m, 3H), 8.21 – 8.15 (m, 3H), 8.14 – 8.11 (m, 1H), 8.06 (t, J = 7.6 Hz, 1H), 4.81 – 4.77 (m, 1H), 1.61 – 1.59 (m, 3H).

Table S2. Optimization of reaction conditions for the synthesis of product **D**.

**C**/eq.	Ala/eq.	Condensation reagent/eq.	Base/eq.	Solvent	Yield/%
1	1.1	-	$KHCO_3$/1.1	dry DMF	45
1	1.2	PyBOP/1.5	DIPEA/3	dry DCM	-
1	1.1	HATU/1.2	NMM/1.2	dry DCM	-

All reactions were performed at rt.

Benzyl (pyrene-1-carbonyl)-L-alanyl-L-tyrosinate (E)

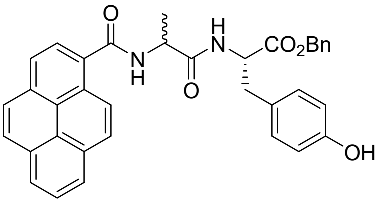

(Pyrene-1-carbonyl)-L-alanine (30 mg, 0.095 mmol) was dissolved in DMF (2mL) with addition of TBTU (34 mg, 0.104 mol) and DIPEA (18 µL, 0.104 mmol). Boc-deprotected Tyr-OBn (50 mg, 0.095 mmol) was dissolved in DMF (1 mL) with addition of DIPEA (18 µL, 0.104 mmol) and was added dropwise. Reaction mixture was stirred 24h at rt. The solvent was evaporated. To the residue, ethyl acetate (10 mL) and ammonium chloride (10 mL) were added and the product was extracted with ethyl acetate (3×10 mL). The organic extracts were dried over anhydrous Na_2SO_4, filtered and the solvent was removed on a rotational evaporator. The product was purified on a column of silica gel by use of EtOAc:CH_2Cl_2 = 1:2 and was isolated as colorless oil (d.r. 1:1; 21 mg, 0.037 mmol, 39%).

^{1}H NMR (300 MHz, DMSO): δ = 9.24 – 9.21 (m, 1H), 8.81 – 8.74 (m, 1H), 8.59 – 8.52 (m, 2H), 8.47 – 8.41 (m, 1H), 8.40 – 8.29 (m, 6H), 8.27 – 8.20 (m, 5H), 8.18 – 8.06 (m, 4H), 7.47 – 7.23 (m, 10H), 7.12 – 6.99 (m, 4H), 6.72 – 6.59 (m, 4H), 5.20 – 5.05 (m, 4H), 4.75 – 4.50 (m, 4H), 3.06 – 2.83 (m, 4H), 1.41 – 1.32 (m, 3H), 1.31 – 1.19 (m, 3H).

Table S3. Optimization of reaction conditions for the synthesis of product **E**.

**D**/eq.[a]	Boc-Tyr-OBn/eq.[b]	Condensation reagent/eq.	Base/eq.	Solvent	Yield/%
1	1	HATU/1.1	NMM/2.2	DMF	-
1	1	HATU/1.1	NMM/2.2	ACN	-
1	1	TBTU/1.1	DIPEA/2.2	DMF	39[c]
1	1	TBTU/1.1	DIPEA/2.2	DCM	-

[a]All reactions were performed at rt; [b]Boc-Tyr-OBn was deprotected with TFA/DCM before using in the reaction; [c]Two diastereoisomers were detected in ^{1}H NMR.

Benzyl (*S*)-3-(3-((dimethylamino)methyl)-4-hydroxyphenyl)-2-((*S*)-2-(pyrene-1-carboxamido)propanamido)propanoate (F)

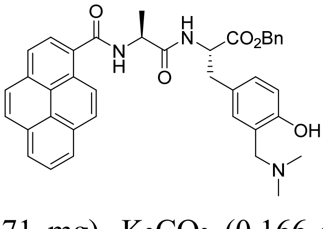

Benzyl (pyrene-1-carbonyl)-L-alanyl-L-tyrosinate (**E**; 0.124 mmol, 71 mg), K_2CO_3 (0.166 mmol, 34 mg) and Eschenmoser's salt (0.248mmol, 24 mg) were dissolved in dry DCM (6 mL) under nitrogen at rt. The reaction mixture was stirred 72h and solvent was removed on a rotational evaporator. The product was not obtained, based on HPLC

and TLC analysis after 96h. The reaction mixture was heated at the temperature of reflux and after 24h the product was not obtained.

HPLC method for the semipreparative purification of 1 and 2.

1 was isolated from by semipreparative HPLC separation on a Phenomenex Jupiter C18 5 μm 300 Å column according to the method given in Table S4.

Table S4. Method used for the semipreparative HPLC purification of **1** on a Phenomenex Jupiter C18 5 μm 300 Å column:

t / min	% A (MeOH)	% B (MeOH : H_2O = 1:1) 0.1% TFA
0	0	100
20	0	100
40	100	0
60	100	0
A: CH_3OH, 100 %<br>B: CH_3OH–H_2O (1:1 v/v) + TFA, 0.1%<br>Flow rate: 3.5 ml/min		

2. Photophysical measurements

Absorption spectra were recorded on a PG T80/T80+ or Agilent Cary 100 UV-Vis spectrophotometer at rt. Fluorescence measurements were performed on an Edinburgh FS5 fluorimeter. The samples were dissolved in CH_3CN and the concentrations were adjusted to absorbances of less than 0.1 at the excitation wavelengths (270–330 nm). The fluorescence spectra were obtained by using monochromator slits corresponding to the bandpass of 1.0 nm for both the excitation and the emission. The spectra were corrected with respect to the output of the Xe-lamp and to the transmission through the detection optics. Fluorescence quantum yields were determined by comparison of the integral of the emission bands with the one of quinine sulfate (Φ_{254} = 0.546) [4]. Prior to the measurements, the solutions were purged with Ar for 20 min. Fluorescence measurements were performed by exciting sample at four different wavelengths and the average value was calculated (Eq. S1). The measurement was performed at 25°C.

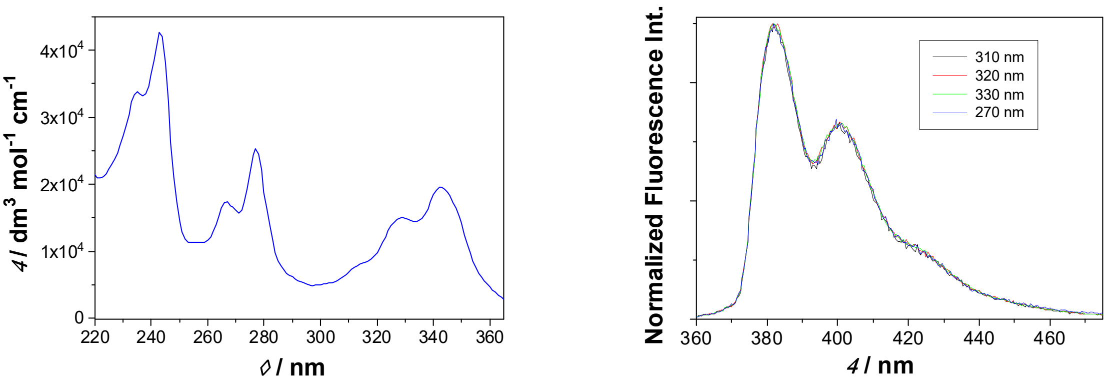

Fig S1. Absorption (left) and normalized emission (right) spectra of **7** in CH_3CN, obtained by exciting at different wavelengths (λ_{exc} =270, 310, 320, and 330 nm).

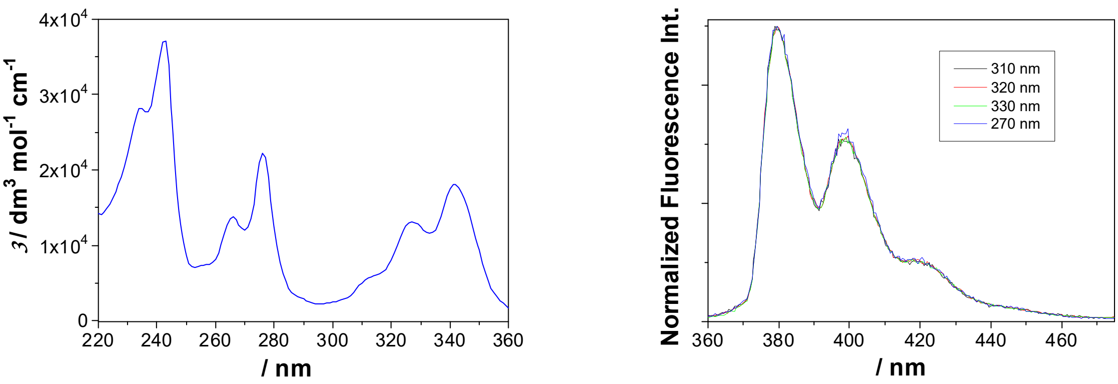

Fig S2. Absorption (left) and normalized emission (right) spectra of **8** in CH_3CN, obtained by exciting at different wavelengths (λ_{exc} =270, 310, 320, and 330 nm).

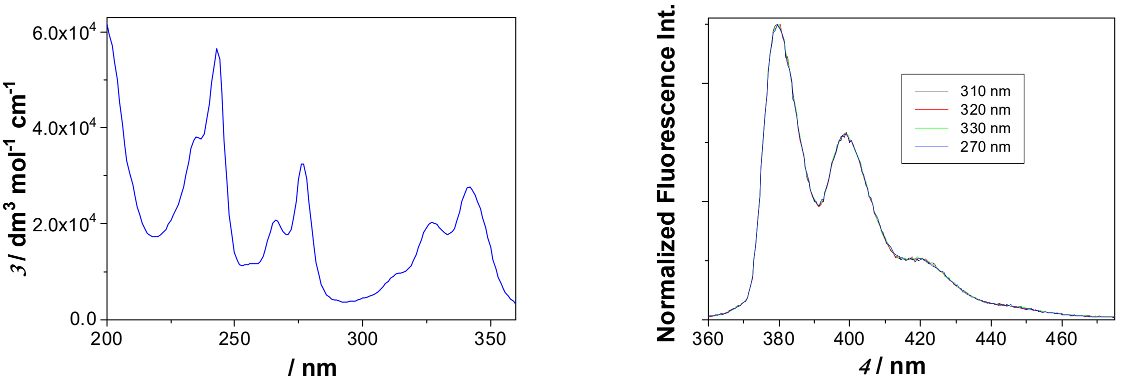

Fig S3. Absorption (left) and normalized emission (right) spectra of **5** in CH_3CN, obtained by exciting at different wavelengths (λ_{exc} =270, 310, 320, and 330 nm).

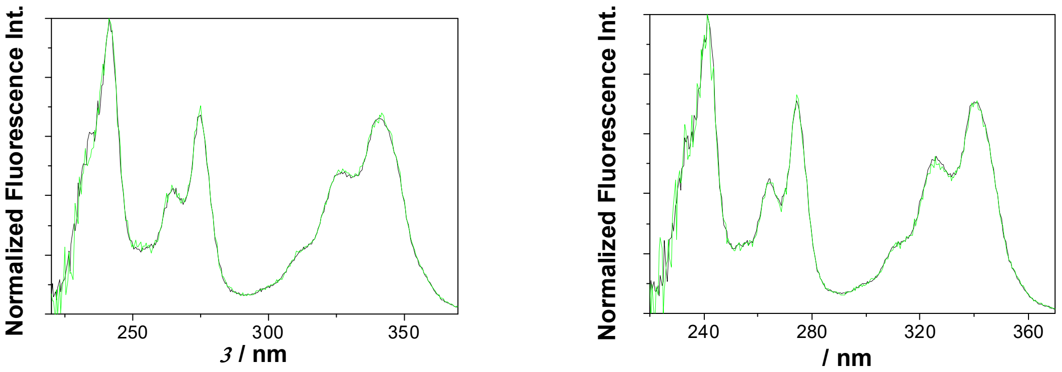

Fig S4. Normalized excitation spectra for **7** (left) and **8** (right) in CH_3CN, detected at different wavelengths (λ_{em} = 400 and 420 nm).

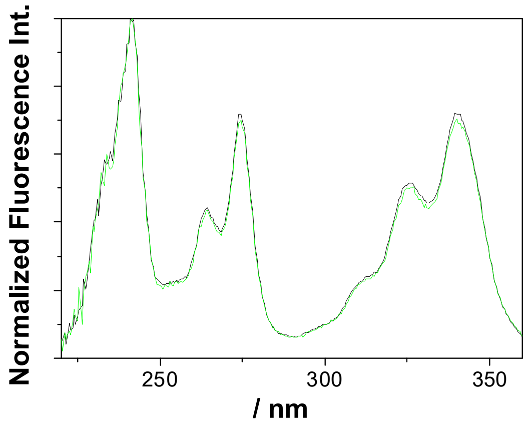

Fig S5. Normalized excitation spectra for **5** in CH_3CN, detected at different wavelengths (λ_{em} = 400 and 420 nm).

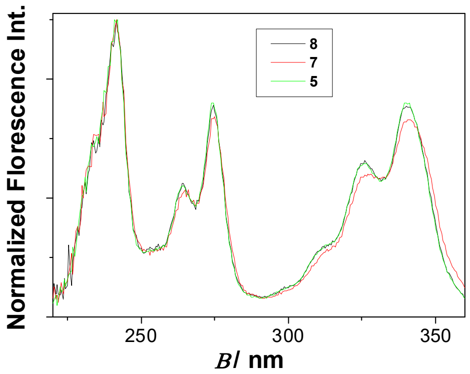

Fig S6. Normalized excitation spectra for **5**, **7**, and **8** in CH_3CN, detected at 400 nm.

The following equation was used for the determination of fluorescence quantum yields:

$$\Phi = \Phi_R \frac{I}{I_R}\frac{A_R}{A}\left(\frac{n_D}{n_D^R}\right)^2 \qquad \text{(S1)}$$

wherein

Φ - quantum yield of fluorescence

Φ_R - quantum yield of fluorescence of reference compound, Fluorescence quantum yields for **7**, **8** and **5** were measured by use of quinine sulfate in aqueous 0.05 M H_2SO_4 ($\Phi_f = 0.546$) [4]

I - intensity of fluorescence (integral of the corrected emission spectrum)

I_R - intensity of fluorescence (integral of the corrected emission spectrum) for the reference compound

A - absorbance of the solution at the excitation wavelength

A_R - absorbance of the solution of the reference compound at the excitation wavelength

n_D - refractive index of the solvent (acetonitrile)

n_D^R - refractive index of the solvent use to dissolve the reference compound (H_2O)

The same solutions as in fluorescence measurements were used for time correlated single photon counting (TC-SPC) measurements which were performed on an Edinburgh FS5 spectrometer equipped with a pulsed LED at 280 and 340 nm, the duration of the pulse was ≈800 ps and Fluorescence signals were monitored at 400 nm over 1023 channels with the time increment of ≈100 ps/channel. The decays were collected until they reached 3000 counts in the peak channel. A suspension of silica gel in H_2O was used as a scattering solution to obtain instrument response function (IRF). The measurement was performed at 25°C. The obtained histograms were fit as sums of exponentials using Gaussian-weighted non-linear least-squares fitting based on Marquardt-Levenberg minimization implemented in the software package from

the instrument. The fitting parameters (decay times and pre-exponential factors, Eq S2) were determined by minimizing the reduced chi-square χ^2 and graphical methods were used to judge the quality of the fit that included plots of the weighted residuals vs. channel number. Decays of fluorescence were fit to a sum of exponentials according to the following expression:

$$F(t) = A + \alpha_1 \exp\left(-\frac{t}{\tau_1}\right) + \alpha_2 \exp\left(-\frac{t}{\tau_2}\right) + \alpha_3 \exp\left(-\frac{t}{\tau_3}\right) + \dots \qquad \text{(S2)}$$

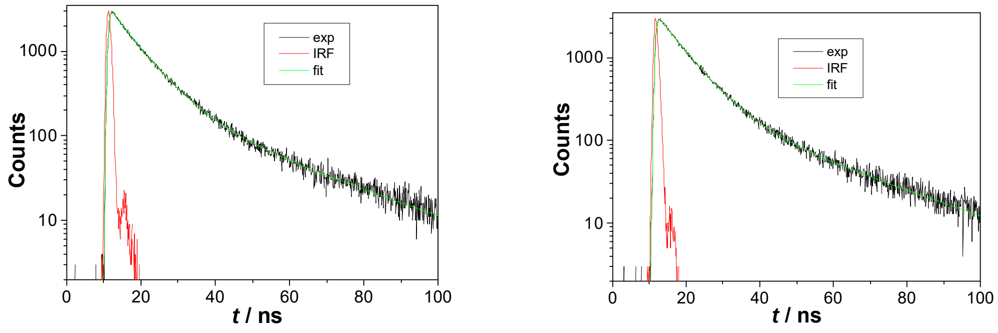

Fig S7. Decay of fluorescence at 400 nm for **7** in CH_3CN; left; λ_{exc} = 280 nm; right λ_{exc} = 340 nm. Black: experimental values, red: IRF, and green: fit to a sum of three exponents.

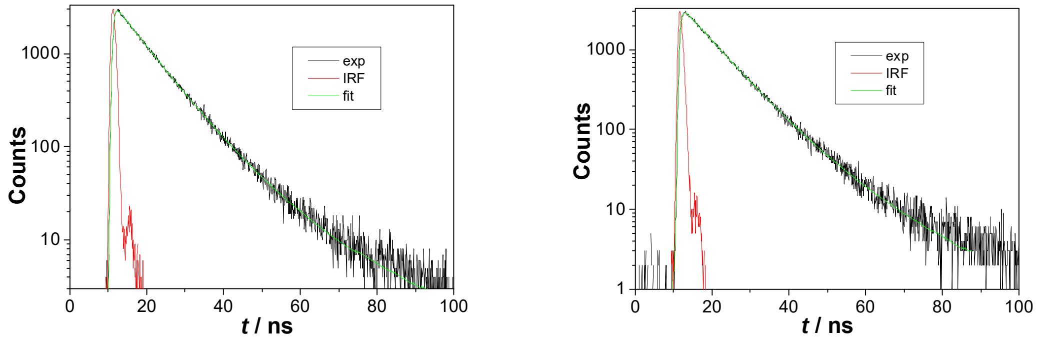

Fig S8. Decay of fluorescence at 400 nm for **8** in CH_3CN; left; λ_{exc} = 280 nm; right λ_{exc} = 340 nm. Black: experimental values, red: IRF, and green: fit to a sum of three exponents.

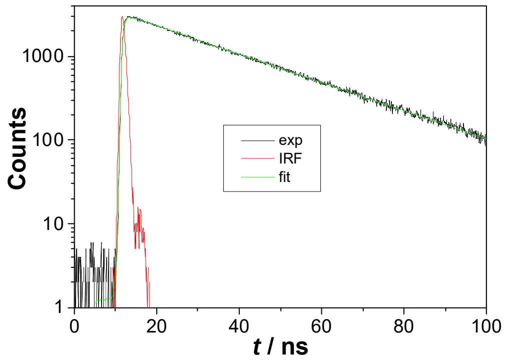

Fig S9. Decay of fluorescence at 400 nm for **5** in CH_3CN; λ_{exc} = 340 nm. Black: experimental values, red: IRF, and green: fit to a sum of three exponents.

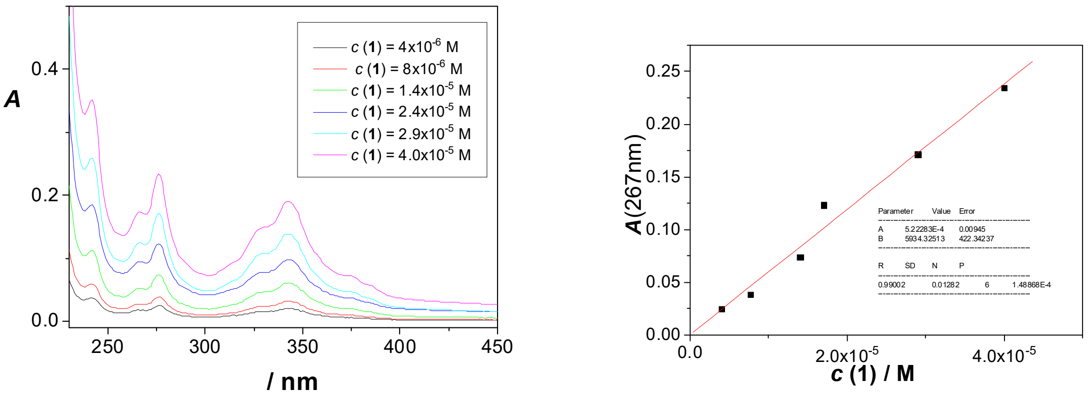

Fig S10. Absorption spectra (left) and the absorbance at 267 nm (right) depending on the concentration of **1** ($4 \times 10^{-6} - 4 \times 10^{-5}$ M) in sodium cacodylate buffer (pH 7.0, 50 mM), aqueous (<1.0% DMSO) solution. Intensity maxima at 386 nm is linearly dependent on the concentration up to 1.5×10^{-5} M.

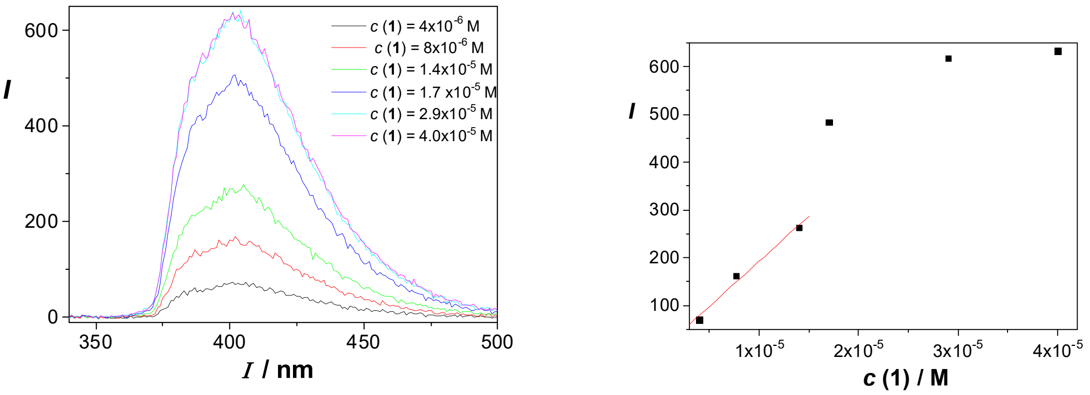

Fig S11. Fluorescence spectra (λ_{exc} = 330 nm) (left) and the fluorescence intensity at 386 nm (right) depending on the concentration of **1** ($4 \times 10^{-6} - 4 \times 10^{-5}$ M) in sodium cacodylate buffer (pH 7.0, 50 mM), aqueous (<1.0% DMSO) solution. Intensity maxima at 386 nm is linearly dependent on the concentration up to 1.5×10^{-5} M.

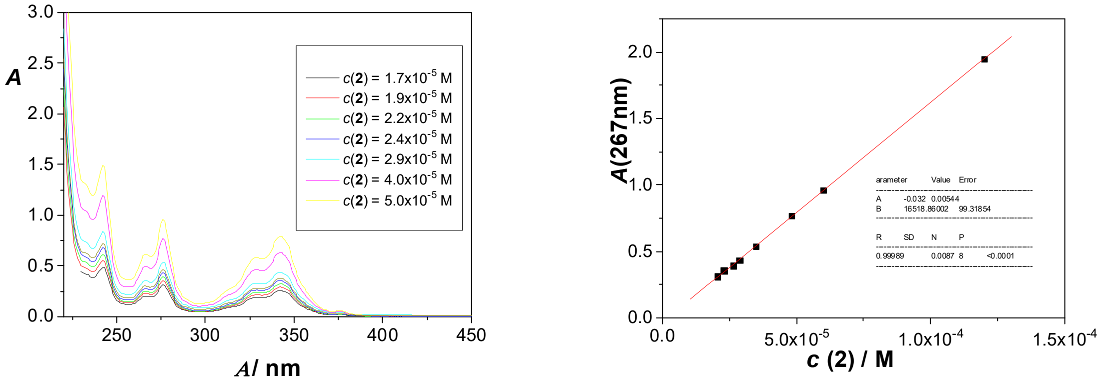

Fig S12. Absorption spectra (left) and the absorbance at 267 nm (right) depending on the concentration of **2** ($4 \times 10^{-6} – 4 \times 10^{-5}$ M) in sodium cacodylate buffer (pH 7.0, 50 mM), aqueous (<1.0% DMSO) solution. Intensity maxima at 386 nm is linearly dependent on the concentration up to 1.5×10^{-5} M.

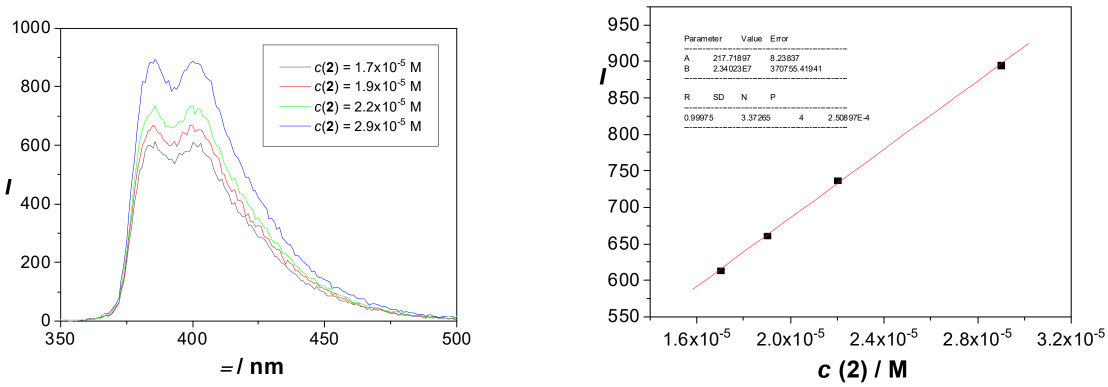

Fig S13. Fluorescence spectra (λ_{exc} = 330 nm) (left) and the fluorescence intensity at 386 nm (right) depending on the concentration of **2** (1.7×10^{-5} - 2.9×10^{-5} M) in sodium cacodylate buffer (pH 7.0, 50 mM), aqueous (<1.0% DMSO) solution. The intensity is linearly dependent on to the concentration up to 2.9×10^{-5} M.

3. Photochemical reactivity

Quantum yield for photohydrolysis reactions were performed in quartz fluorescence cells that were irradiated only from the front side. The KI/KIO_3 was used as actinometer (Φ_{254} = 0.74)[5]. Freshly prepared solutions of compounds **7** and **8**, and actinometer were adjusted to have absorbance of 0.4 - 0.8 at 254 nm, and were purged with a stream of N_2 for 20 min. After adjustment of the concentrations and measurement of the corresponding UV-vis spectra, the solutions were purged with a stream of Ar (20 min), and then, sealed with a cap. The cells were placed in a holder which ensured equal distance of all samples from the lamp and were irradiated at the same time in the reactor with 1 lamp (8 W) at 254 nm for 60 min. Before and after the irradiation, the samples were taken from the cells using a syringe and analyzed by HPLC to determine the photochemical conversions. The conversion did not exceed 30% to avoid a change of the absorbance, or filtering of the light by the product. The measurements were performed in duplicates. Quantum yields were calculated according to Eqs S3-S7.

The number of absorbed photons for the KIO_3/KI was calculated from:

$$n\text{ (absorbed photons)} = \frac{\Delta A_{352} \times V_{irr}}{\varepsilon_{352} \times l \times \Phi_{lit.}} \qquad \text{(S3)}$$

where:

ΔA_{352} - absorbance difference at 352 nm for the irradiated and non-irradiated sample

V_{irr} - volume of the solution that was irradiated

ε_{352} - molar absorption coefficient for I_3^- in solution that contains iodides and iodates, 27600 M^{-1} cm^{-1}

l - length of the optical path (1 cm in all experiments)

$\Phi_{lit.}$ - literature value for quantum yield of actinometer (Φ_{254} = 0.74)

Quantum yield of photochemical reaction was calculated according to:

$$c(I^-) = A_{300} / 1.061 \text{ [M]} \qquad \text{(S4)}$$

$$\Phi = 0.75 \times [1 + 0.02(T/°C - 20.7)] \times [1+0.23(c(I^-) - 0.577)] \qquad \text{(S5)}$$

For the solutions with absorbances in the range 0.4-0.8 the number of absorbed photons was calculated according to:

$$n\text{ (absorbed photons)} = n\text{ (total photons)} \times (1-T) \qquad \text{(S6)}$$

The quantum yield of photohydrolysis was calculated according to:

$$\Phi_R = \frac{A_{254} \times V_{irr} \times x\text{ (photoproduct)}_{\text{HPLC}}}{\varepsilon_{254} \times l \times x\text{(total photons)} \times (1-T_{254})} \qquad \text{(S7)}$$

where:

x (photoproduct)$_{\text{HPLC}}$ – photochemical conversion of reactant determined by HPLC.

Table S5. Parameters in the experiments for the determination of the photomethanolysis reaction quantum yield (Φ_R)

Compound	c/(10^{-5}) M[a]	t_{irr}/min[b]	x(photoprod.)[c]	rt_{pp} (HPLC)/ min[d]	Φ_R / (10^{-2})
**7** exp 1	7.76	61	0.228	23.782	1.30
**7** exp 2	4.19	60	0.241	23.787	1.36
**8** exp 1	9.52	60	0.310	23.717	2.51
**8** exp 2	5.73	61	0.290	23.716	2.61

[a] Concentration of the irradiated peptide **7** or **8**. [b] Irradiation time. [c] Conversion to the methanolysis photoproduct determined by HPLC. [c] The photoproduct retention time. [d] Quantum yield of the methanolysis determined according to Eqs. S3-S7.

4. Steric properties of studied DNA and RNA

Table S6. Groove widths and depths for the studied nucleic acid conformation [6,7].

Structure type	Groove width [Å]		Groove depth [Å]	
	major	minor	major	minor
[a] poly rA – poly rU	3.8	10.9	13.5	2.8
[b] *ct*-DNA (48% of GC-pairs)	11.4	3.3	7.5	7.9
[b] poly dAdT – poly dAdT	11.2	6.3	8.5	7.5
[c] poly dGdC – poly dGdC	13.5	9.5	10.0	7.2

[a] A - helical structure

[b] B - helical structure

[c] B - helical structure with sterically blocked minor groove by amino groups of guanines

5. Non-covalent binding to polynucleotides

Preparation of the Annealed Double Stranded Oligonucleotides

Stock solutions of dA_{10} and dT_{10} ($c = 1.0 \times 10^{-3}$ M), were prepared in ammonium acetate buffer. The solution of dA_{10} (500 μL) was mixed with the solution of dT_{10} (500 μL) in a UV cuvette. In order to anneal the polynucleotides their mixture was heated to 90 °C for 5 min, then slowly cooled to rt and stored for 24 h at 4 °C before the use in the experiments. The solutions of annealed oligonucleotides were used in the photochemical alkylation experiments.

Thermal denaturation experiments

A stock solution of **1** and **2** were prepared in DMSO ($c = 2.0 \times 10^{-2}$ M), whereas the stock solutions of *ct*-DNA and pApU were prepared in aqueous cacodylate buffer (pH = 7.0, 50 mM) c(*ct*-DNA) = 2.6×10^{-3} M, c(pApU) = 6.5×10^{-3} M.

In the thermal denaturation experiments, the nucleotide solution (*ct*-DNA, and pApU) was diluted in a quartz UV-vis cell (with the optical path of 1.0 cm) by cacodylate buffer to the concentration of $c = 2.0 \times 10^{-5}$ M, and the appropriate amount of the solution of the peptide was added to reach the desired ratio r ([peptide]/[DNA]) = 0.3. Finally, the covalently bound peptide with *ct*-DNA was tested by irradiating *ct*-DNA the above mentioned solution ([peptide]/[DNA]) = 0.3) before measuring thermal denaturation.

The dependence of the absorbance at 260 nm as a function of temperature was measured on a Jasco V-730 Spectrophotometer. The temperature was measured from 25 °C to 95 °C in intervals of 0.5 °C. The denaturation temperature T_m values are the midpoints of the transition curves, determined from the sigmoidal fit of thermal melting. ΔT_m values were calculated by subtracting T_m of the free nucleic acid from that of the respective complex with ΔT_m values are the average of at least two independent measurements and the error in ΔT_m is ± 0.5 °C.

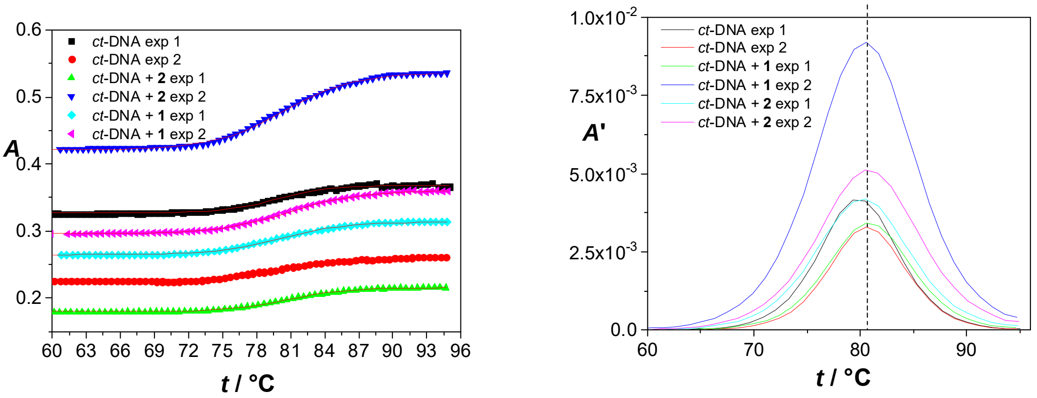

Fig S14. Left: Melting curve of *ct*-DNA upon addition r = 0.3 ([compound/ [polynucleotide]) of **1** and **2** at pH 7.0 (buffer sodium cacodylate, 50 mM); Right: First derivative of the sigmoidal fit of the melting curve.

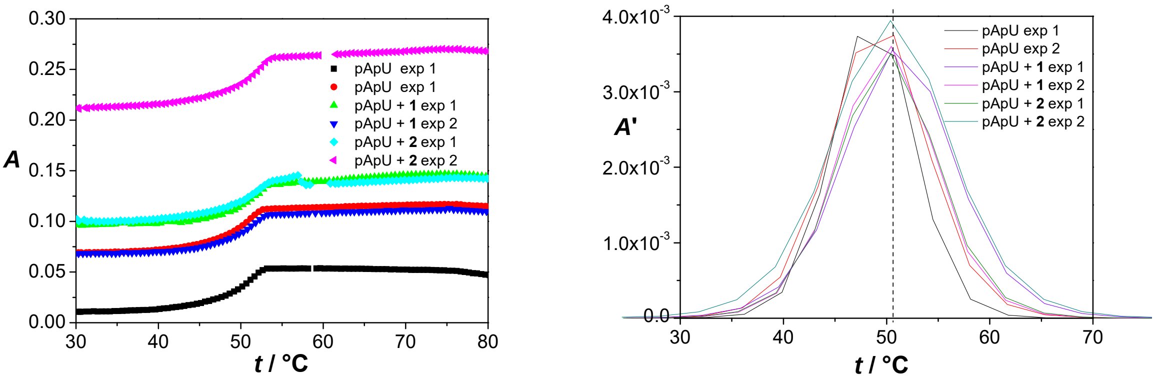

Fig S15. Left: Melting curve of pApU upon addition r = 0.3 ([compound/ [polynucleotide]) of **1** and **2** at pH 7.0 (buffer sodium cacodylate, 50 mM); Right: First derivative of the sigmoidal fit of the melting curve.

Table S7. Values of ΔT_m / °C for **1** and **2** with *ct*-DNA and pA-pU.[a]

	ΔT_m / °C for *ct*-DNA (T_m = 80.5 ± 0.3 °C)	ΔT_m / °C for pApU (T_m = 48.9 ± 0.2 °C)
**1**	0.5 ± 0.8	1.9 ± 0.6
**2**	0.6 ± 0.8	1.4 ± 0.2

[a] The ΔT_m values were calculated from at least two independent measurements. [b] The measurement was conducted for r = 0.3 = [peptide]/[phosphate in polynucleotide]

Fluorescence titrations

Stock solutions of **1** and **2** ($c = 2 \times 10^{-2}$ M) were prepared in DMSO and the solutions of DNA were prepared in aqueous cacodylate buffer (pH = 7.0, 50 mM). For the titrations, the DMSO solutions were diluted in a fluorescence cell (3 mL) with cacodylate buffer (pH 7.0, 50 mM) to reach the concentration $c = 1.0 \times 10^{-6}$ M, the final solution containing >0.5% DMSO. Polynucleotide solutions used for titrations were $c = (3 - 30) \times 10^{-4}$ M. The fluorescence spectra were measured on a Cary Eclipse (Agilent Technologies) at 25 °C. The samples were excited at 330 nm, and the emission was recorded in the range 340–500 nm. The excitation slit was set to the bandpass of 2.5–10 nm, and the emission slit to 5–10 nm. Small aliquots of the polynucleotide solutions were added to the solution of the peptide and after an incubation time of 2 min, fluorescence spectra were taken. Data obtained by fluorescence titrations were processed by nonlinear regression analysis according to the Scatchard model, McGhee, von Hippel formalism [8,9]. Each titration with *ct*-DNA was performed three times, and with other polynucleotides at least twice.

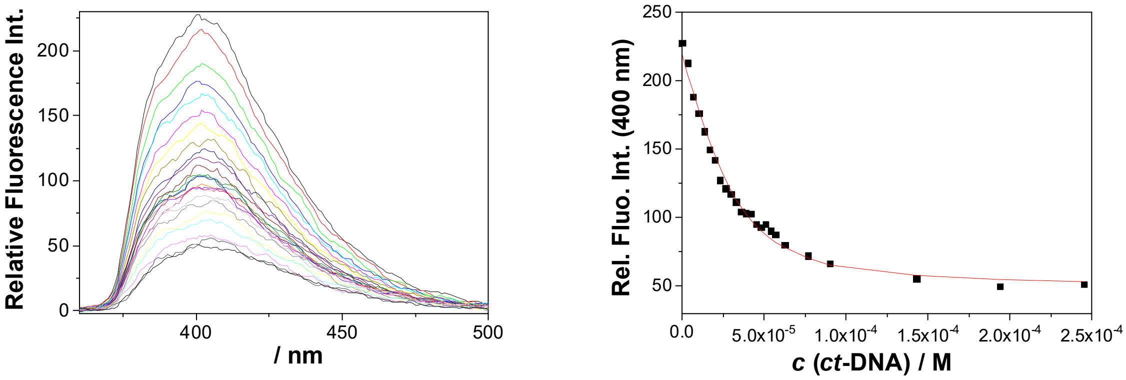

Fig S16. Left: Fluorescence titration for **1** ($c = 1 \times 10^{-6}$ M, $\lambda_{exc} = 330$ nm) with *ct*-DNA in aqueous cacodylate buffer (pH 7.0, 50 mM at 25°C) < 0.5% DMSO; Right: Fluorescence intensity at 400 nm; ■ the experimental values, — fitted to the Scatchard model, fixed $n = 0.3$.

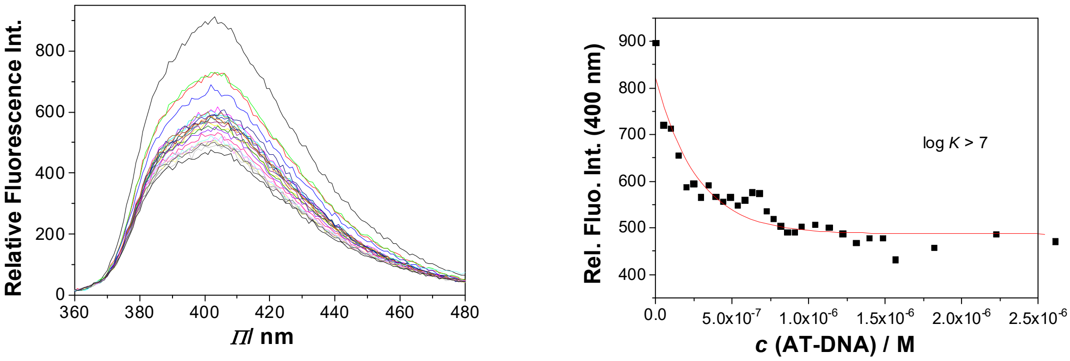

Fig S17. Left: Fluorescence titration for **1** ($c = 1 \times 10^{-6}$ M, $\lambda_{exc} = 330$ nm) with p(dAdT)$_2$ in aqueous cacodylate buffer (pH 7.0, 50 mM at 25°C) < 0.5% DMSO; Right: Fluorescence intensity at 400 nm; ■ the experimental values, — fitted to the Scatchard model, fixed $n = 0.3$.

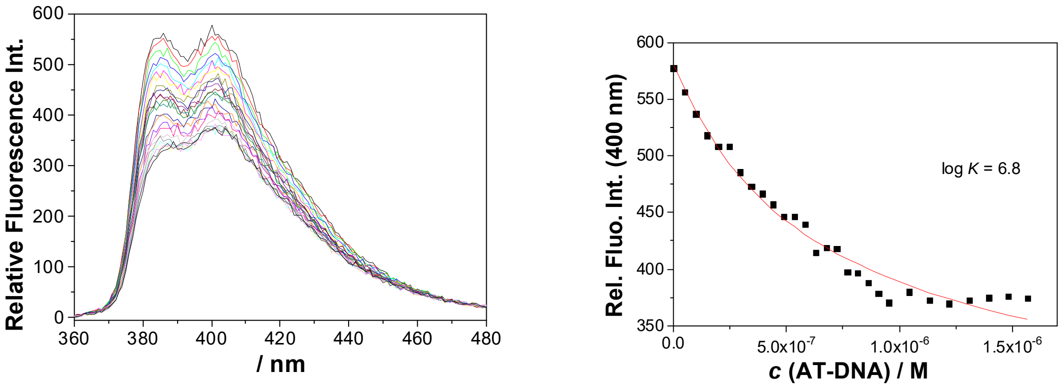

Fig S18. Fluorescence titration for **2** (c = 1 × 10^{-6} M, λ_{exc} = 330 nm) with p(dAdT)$_2$ in aqueous cacodylate buffer (pH 7.0, 50 mM at 25°C) > 0.5% DMSO; Right: Fluorescence intensity at 400 nm; ■ the experimental values, — fitted to the Scatchard model, fixed n = 0.3.

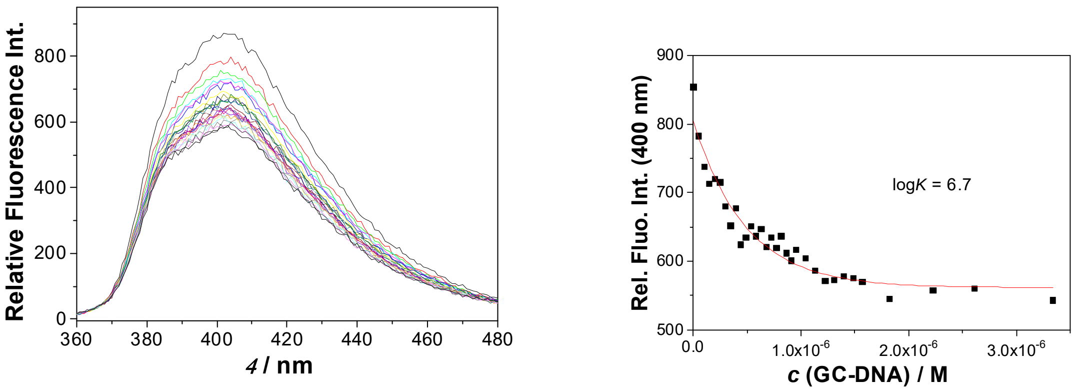

Fig S19. Left: Fluorescence titration for **1** (c = 1 × 10^{-6} M, λ_{exc} = 330 nm) with p(dGdC)$_2$ in aqueous cacodylate buffer (pH 7.0, 50 mM at 25°C) > 0.05% DMSO; Right: Fluorescence intensity at 400 nm; ■ the experimental values, — fitted to the Scatchard model, fixed n = 0.3.

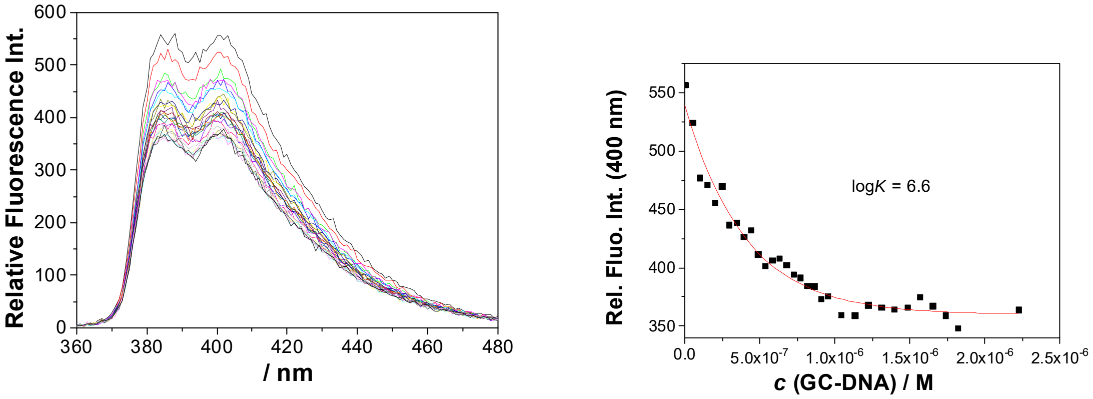

Fig S20. Left: Fluorescence titration for **2** (c = 1 × 10^{-6} M, λ_{exc} = 330 nm) with p(dGdC)$_2$ in aqueous cacodylate buffer (pH 7.0, 50 mM at 25°C) > 0.5% DMSO; Right: Fluorescence intensity at 400 nm; ■ the experimental values, — fitted to the Scatchard model, fixed n = 0.3.

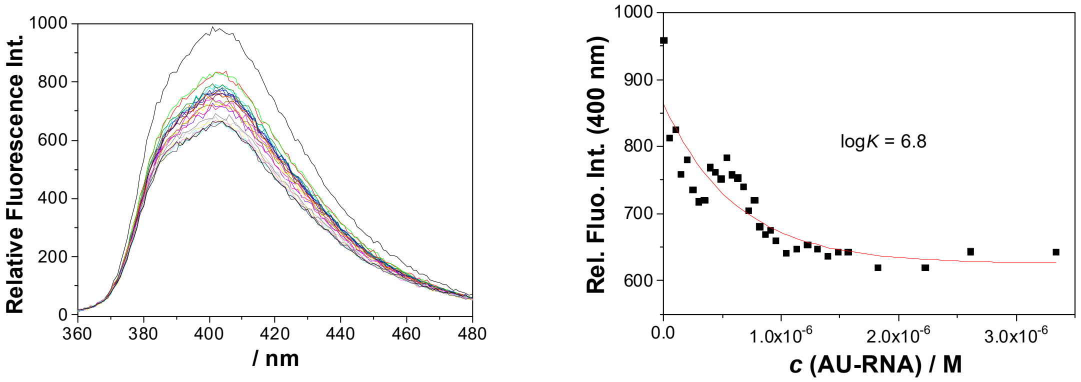

Fig S21. Left: Fluorescence titration for **1** ($c = 1 \times 10^{-6}$ M, $\lambda_{exc} = 330$ nm) with pApU ($c = 0 – 3.5 \times 10^{-6}$ M) in aqueous cacodylate buffer (pH 7.0, 50 mM at 25°C) > 0.5% DMSO; Right: Fluorescence intensity at 400 nm; ■ the experimental values, — fitted to the Scatchard model, fixed $n = 0.3$.

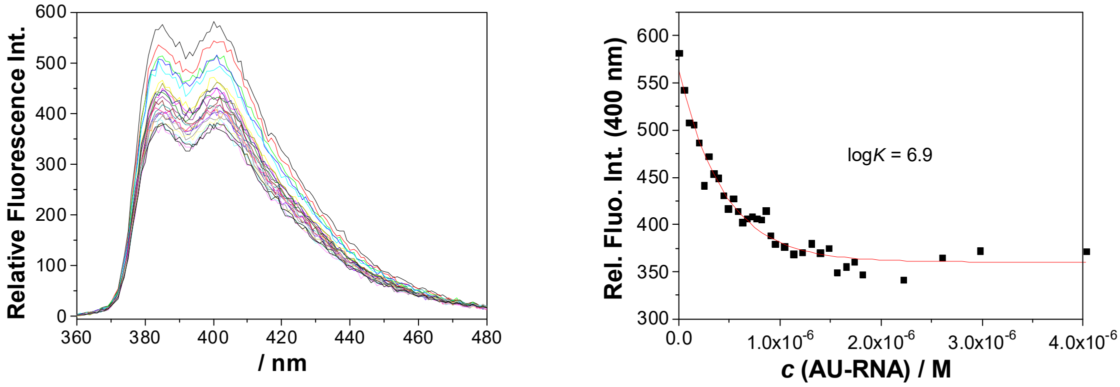

Fig S22. Left: Fluorescence titration for **2** ($c = 1 \times 10^{-6}$ M, $\lambda_{exc} = 330$ nm) with pApU in aqueous cacodylate buffer (pH 7.0, 50 mM at 25°C) > 0.5% DMSO; Right: Fluorescence intensity at 400 nm; ■ the experimental values, — fitted to the Scatchard model, fixed $n = 0.3$.

CD titrations

Circular dichroism spectra were measured on a Jasco J-815 spectrometer in quartz cells with the optical path of 1 cm at 25 °C. Aliquots of the solution of **1** and **2** ($c = 1.0 \times 10^{-3}$ M) in cacodylate buffer (pH = 7.0, 50 mM) were added to the polynucleotide solutions in cells ($c = 2 \times 10^{-5}$ M) to reach the concentration ratio ***r***[peptide]/[polynucleotide] = 0.1 – 1.0 (<0.1% DMSO in measured solution). The CD spectra were recorded in the wavelength range 220–400 nm with the scanning rate of 200 nm/min and three accumulations.

For the measurement of CD spectra of annealed oligonucleotides ds–dA_{10}–dT_{10} the stock solution was diluted with an ammonium acetate buffer (pH = 7.0, 100 mM) to the concentrations of $c = (1–5) \times 10^{-5}$ M.

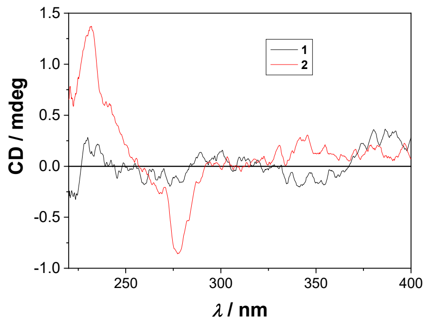

Fig S23. CD spectra of **1** and **2** ($c = 1 \times 10^{-5}$ M) in aqueous cacodylate buffer (pH 7.0, 50 mM at 25°C) with <0.05% DMSO.

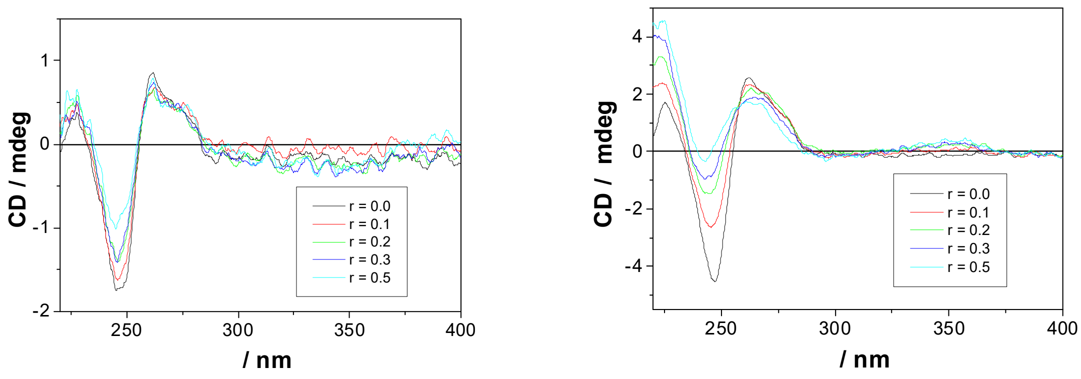

Fig S24. CD titration of $p(dAdT)_2$ ($c = 2 \times 10^{-5}$ M) with **1** (left) and **2** (right) at molar ratios r = [peptide] / [$p(dAdT)_2$] in aqueous cacodylate buffer (pH 7.0, 50 mM at 25°C) with <0.05% DMSO.

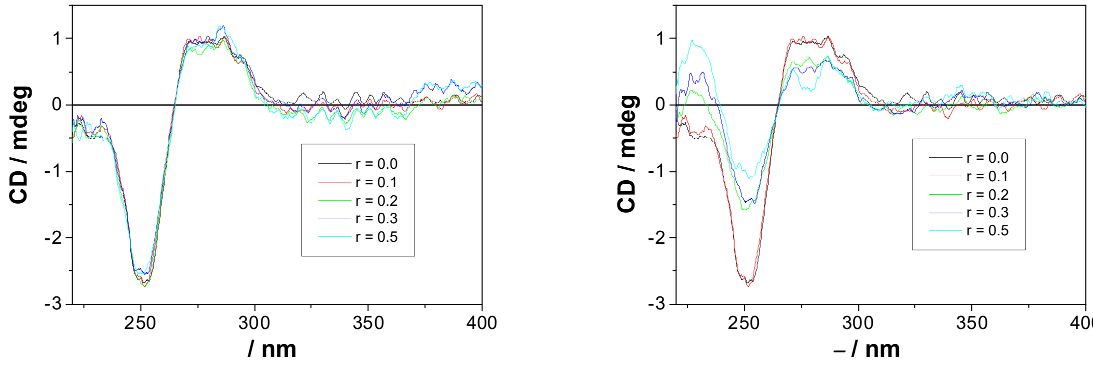

Fig S25. CD titration of p(dGdC)2 ($c = 2 \times 10^{-5}$ M) with **1** (left) and **2** (right) at molar ratios r = [peptide] / [p(dGdC)2] in aqueous cacodylate buffer ((pH 7.0, 50 mM at 25°C) with <0.05% DMSO.

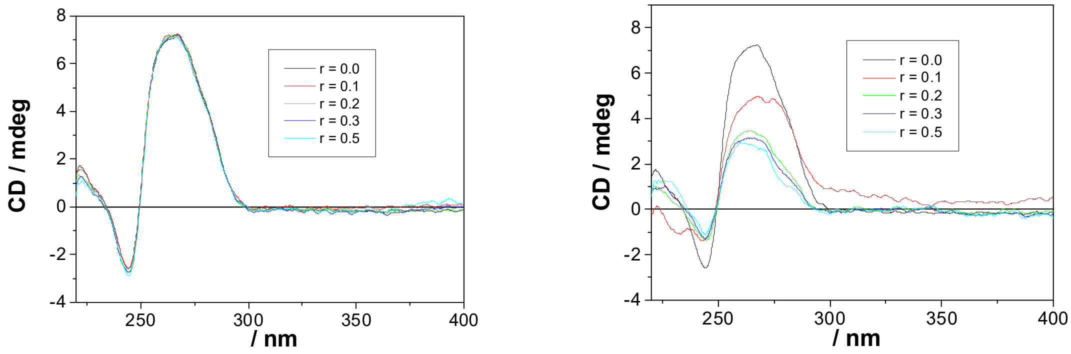

Fig S26. CD titration of pApU (c = 2×10^{-5} M) with **1** (left) and **2** (right) at different molar ratios r = [peptide] / [pApU] in aqueous cacodylate buffer (pH 7.0, 50 mM at 25°C) with <0.05% DMSO.

5. Covalent binding to polynucleotides

The covalent binding of **1** and **2** to polynucleotides was essayed by irradiation (300 nm) in the presence of the model for double stranded DNA and analysis by HPLC. The complementary ds- oligonucleotides were prepared by annealing (dA_{10} with dT_{10}), which was verified by the measurement of the corresponding CD spectra.

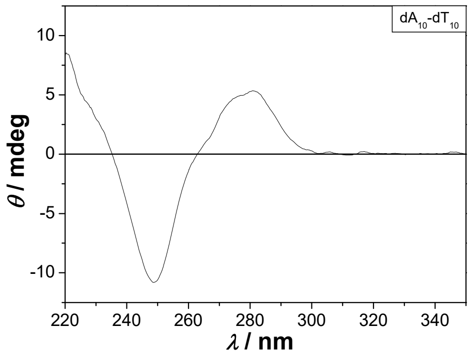

Fig S27. CD spectrum of the annealed dA_{10}– dT_{10} ($c = 1 \times 10^{-6}$ M) in ammonium acetate buffer (100 mM, pH = 7.0).

The stock solution of **2** ($c = 2.0 \times 10^{-2}$ M in DMSO) was diluted with ammonium acetate buffer (100 mM, pH = 7.0) to reach the concentration $c = 5 \times 10^{-4}$ M (2.5% DMSO). The solution of **2** (100 µL, $c = 5.0 \times 10^{-3}$ M) was mixed with the annealed solution of ds–dA_{10}–dT_{10} (350 µL, $c = 5.0 \times 10^{-4}$ M). The cells were irradiated at 300 nm in a Luzchem reactor equipped with 8 lamps with the output of 8 W/lamp for 15 min and 60 min. The composition of the irradiated solutions was analyzed by HPLC.

The analysis of the covalent attachment of peptides to the annealed oligonucleotides was performed on an AGILENT 1260 Infinity II HPLC equipped with a diode array detector and a PLRP–S 5 µm column. Chromatographic method parameters are shown in Table S8.

Table S8. Chromatographic method for the analysis of oligonucleotides, **1**, and **2.**

t / min	% A (NH_4Ac)	% B (MeOH : H_2O = 1:1) 0.1% TFA
0	95	5
5	95	5
15	60	40
20	0	100
25	0	100
32	95	5
40	95	5

A: NH_4Ac (100 mM, pH 7.0)
B: MeCN
Flow rate: 0.5 mL/ min.

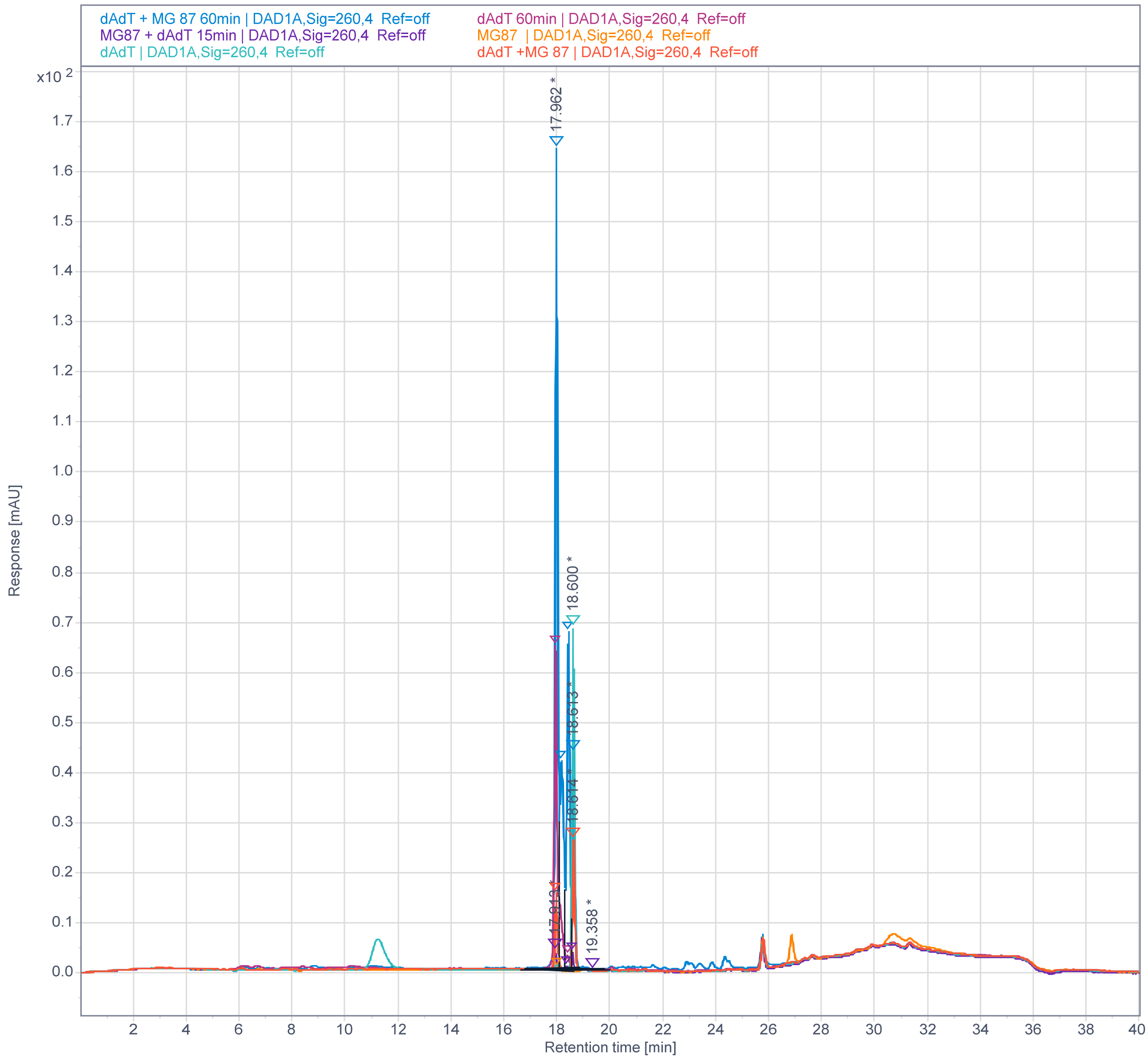

Fig S28. HPLC chromatogram at 260 nm, analysis: — **2** (small peak at 17.976 min); — dA$_{10}$dT$_{10}$ (18.600 min) — dA$_{10}$dT$_{10}$ + **2** (17.957 min, 18.614 min); — dA$_{10}$dT$_{10}$ *hv*(300nm) = 60 min (17.927 min, 18.614 min); — dA$_{10}$dT$_{10}$ + **2** (300nm) = 15 min (17.913 min, 18.588 min); — dA$_{10}$dT$_{10}$ + **2** *hv*(300nm) = 60 min (17.962 min, 18.152 min, 18.415 min, 18.614 min).

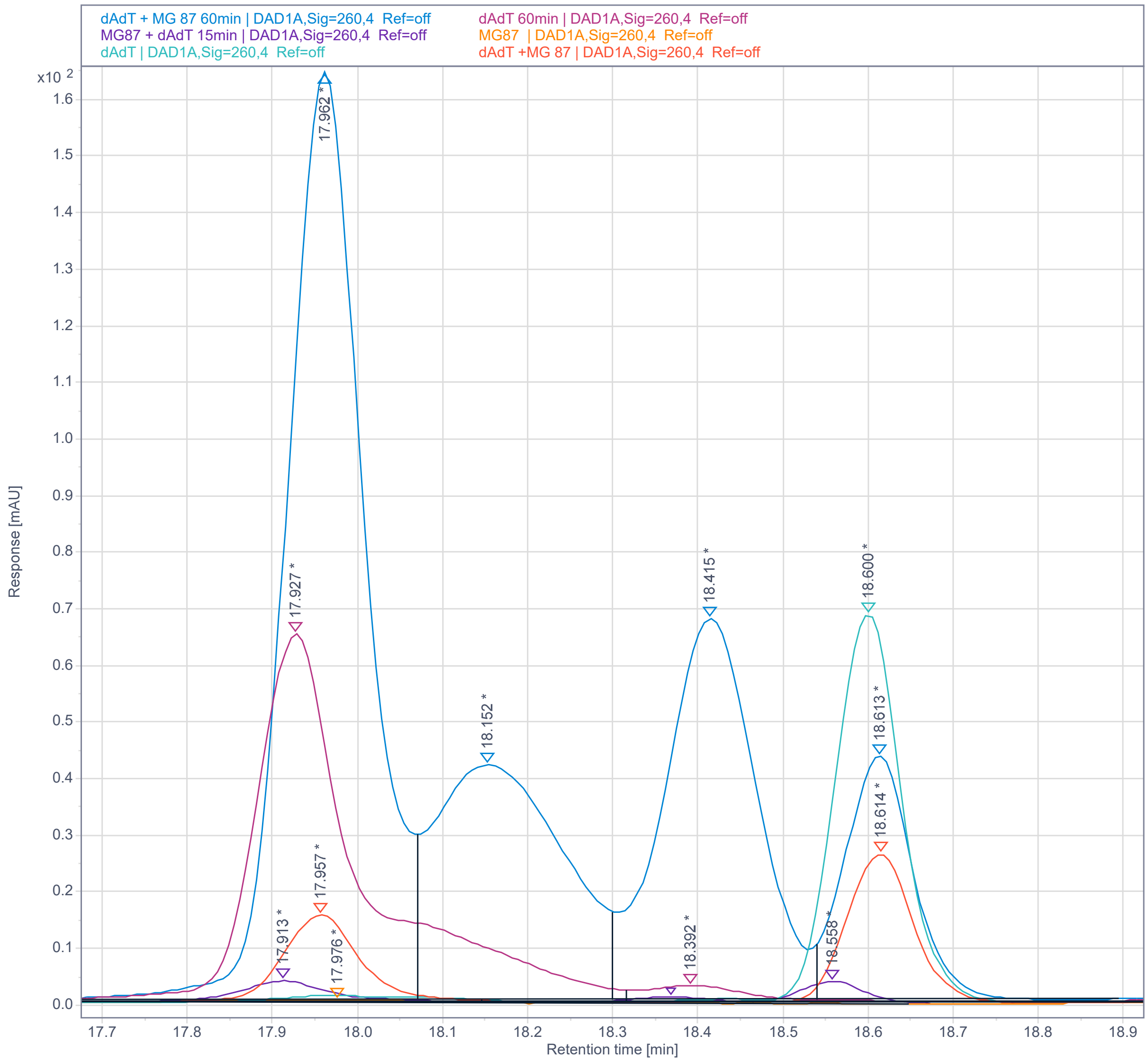

Fig S29. HPLC chromatogram detected at 260 nm (extract), analysis: — **2** (small peak at 17.976 min); — $dA_{10}dT_{10}$ (18.600 min) — $dA_{10}dT_{10}$ + **2** (17.957 min, 18.613 min); — $dA_{10}dT_{10}$ *hν*(300nm) = 60 min (17.927 min, 18.392 min); — $dA_{10}dT_{10}$ + **2** hν(300nm) = 15 min (17.913 min, 18.558 min); — $dA_{10}dT_{10}$ + **2** (300nm) = 60 min (17.962 min, 18.152 min, 18.415 min, 18.614 min).

5. NMR and HRMS spectra

^{1}H NMR (CD_3OD, 300 MHz) of Boc-Tyr[*m*-$CH_2N(CH_3)_2$]-OBn

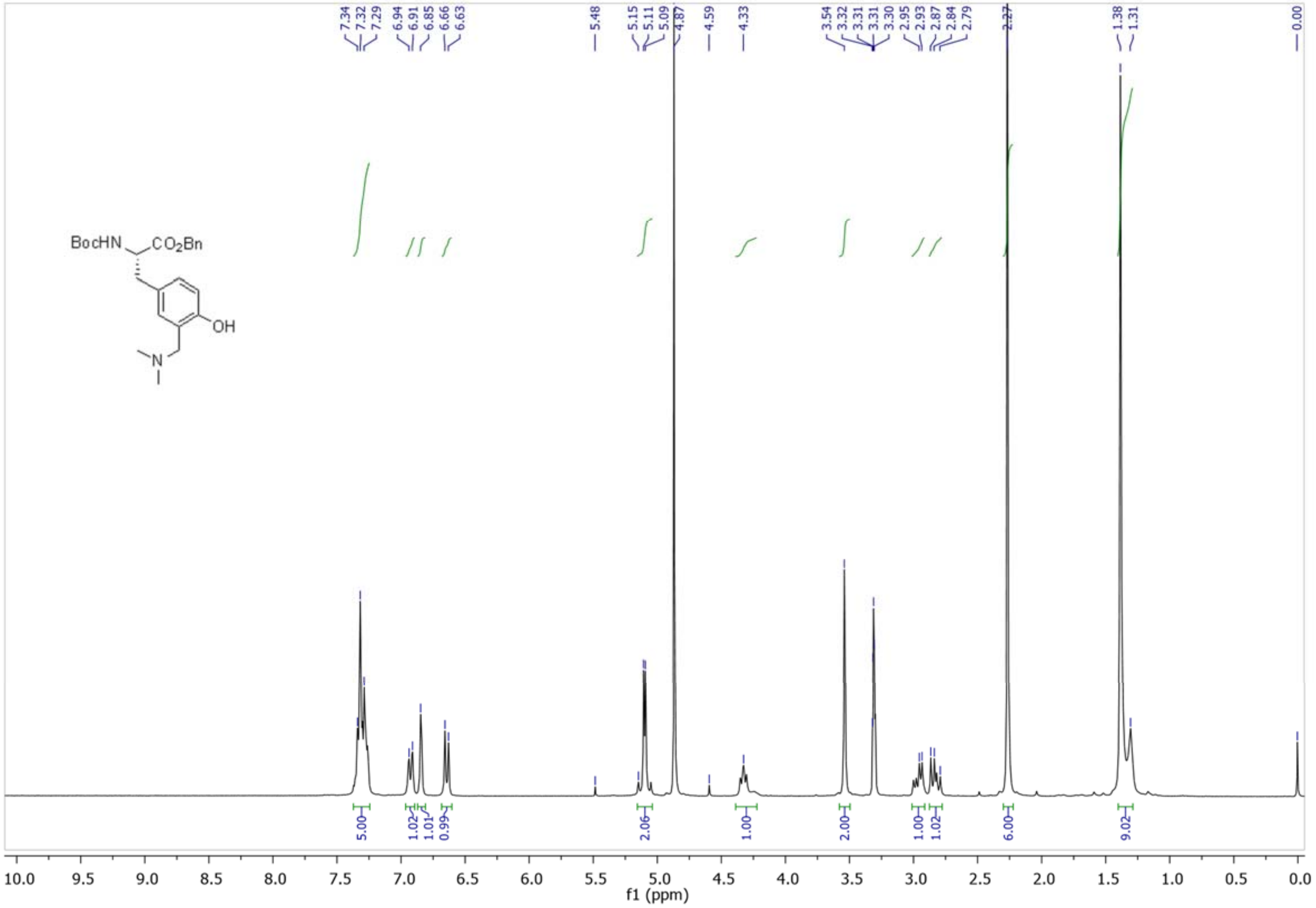

^{13}C NMR (CD_3OD, 75 MHz) of Boc-Tyr[*m*-$CH_2N(CH_3)_2$]-OBn

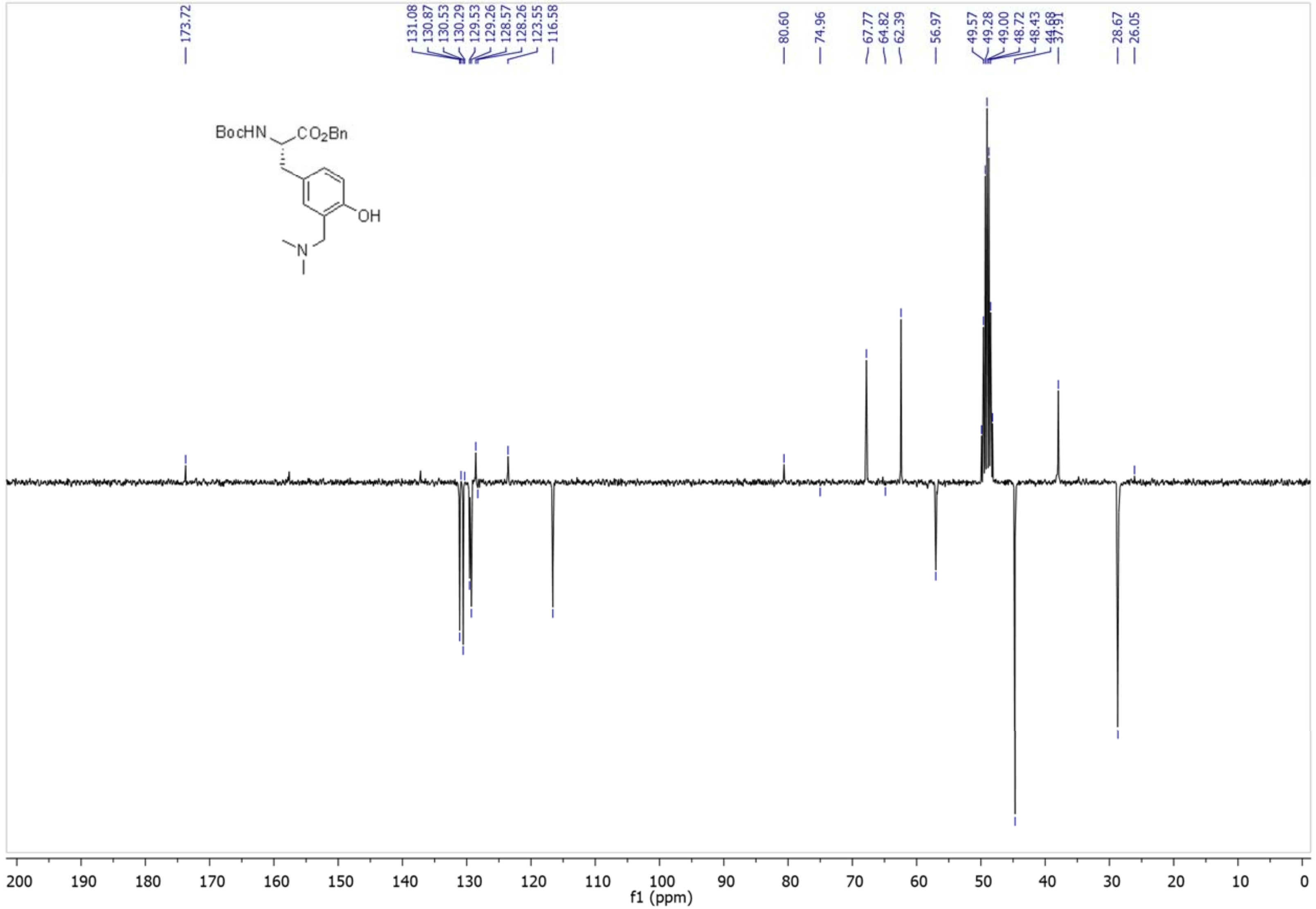

^1H NMR (CD_3OD, 600 MHz) of **D**

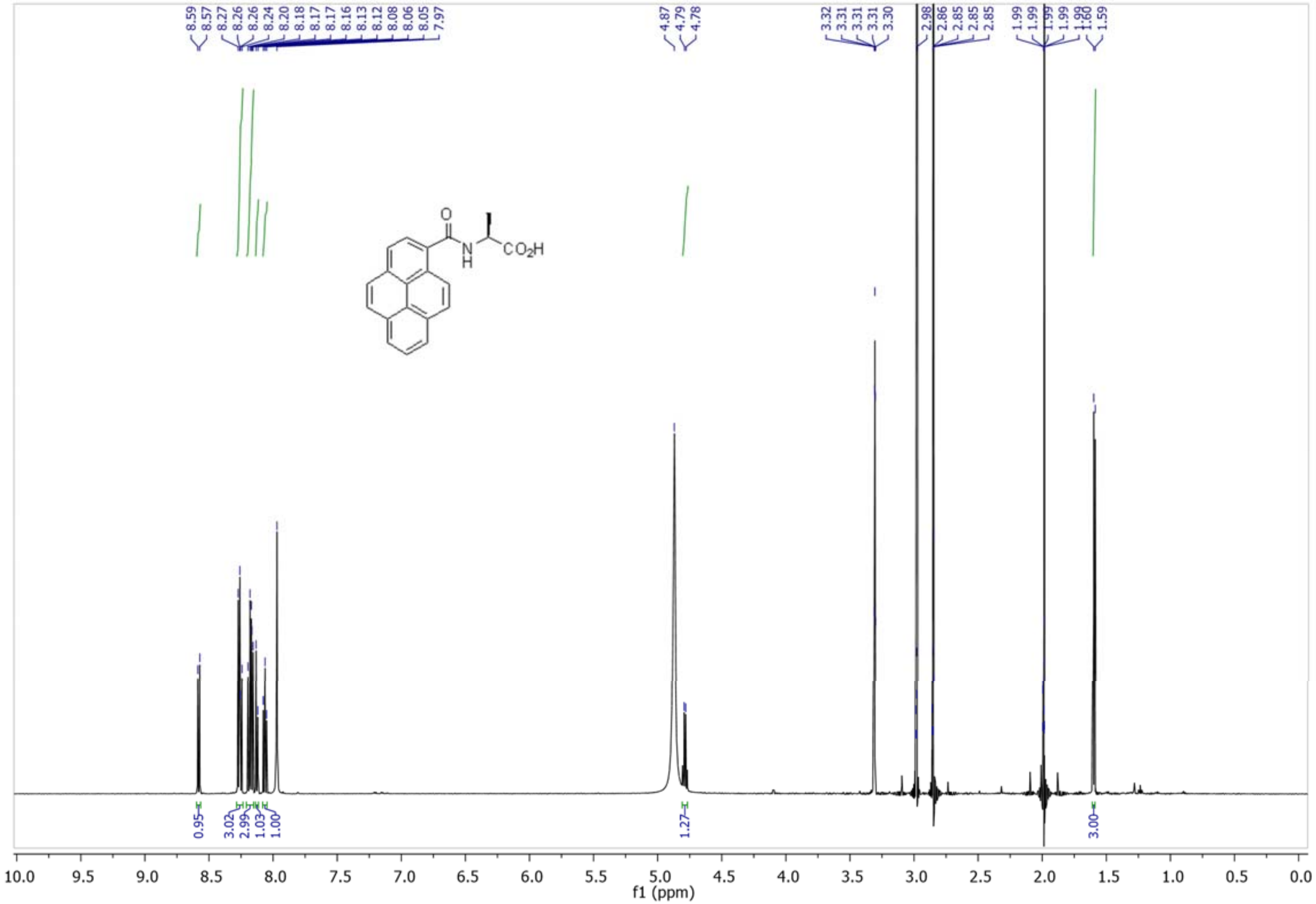

^{1}H NMR (CD3OD, 300 MHz) of **E**

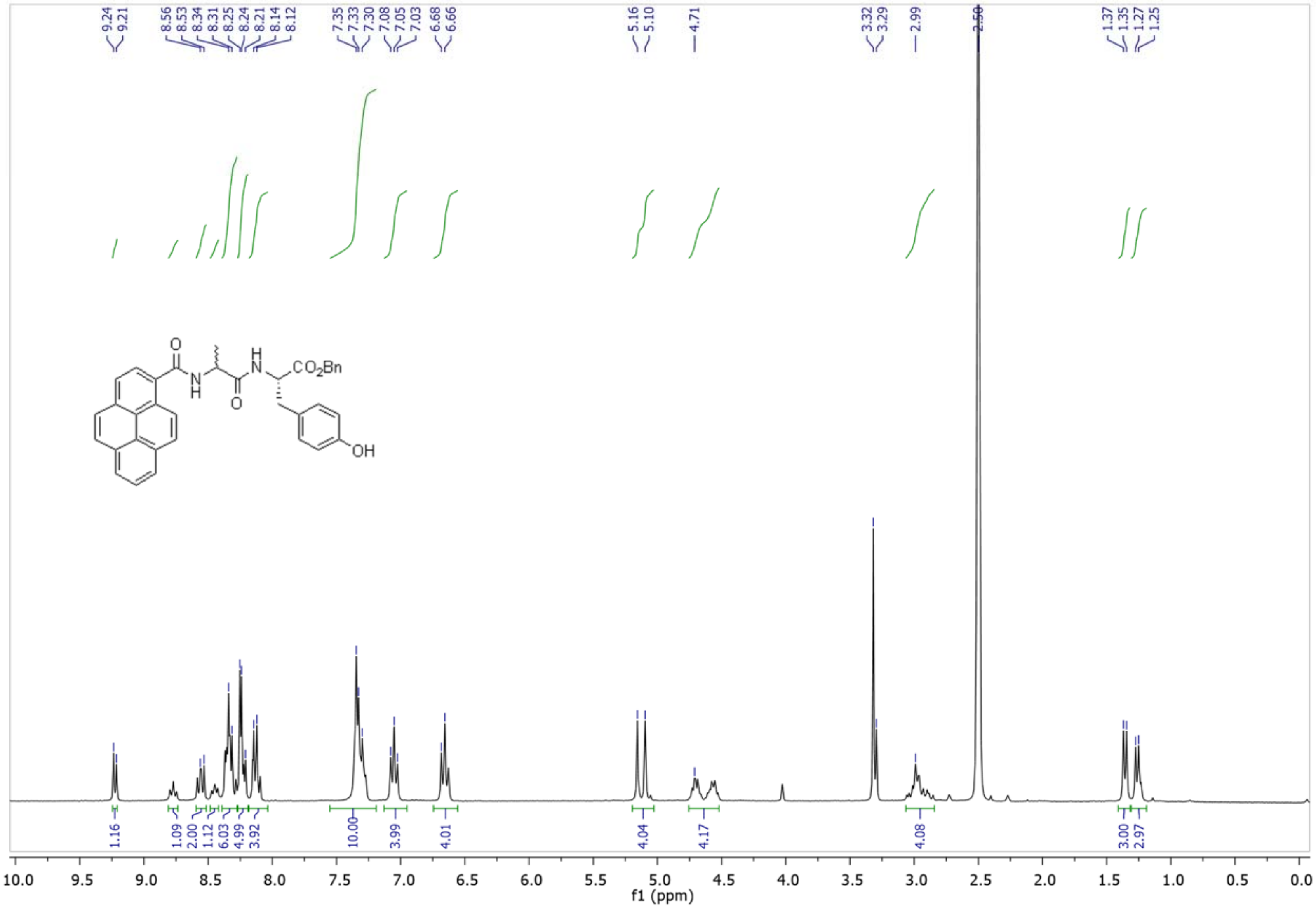

^{1}H NMR ($CDCl_3$, 300 MHz) of **4**

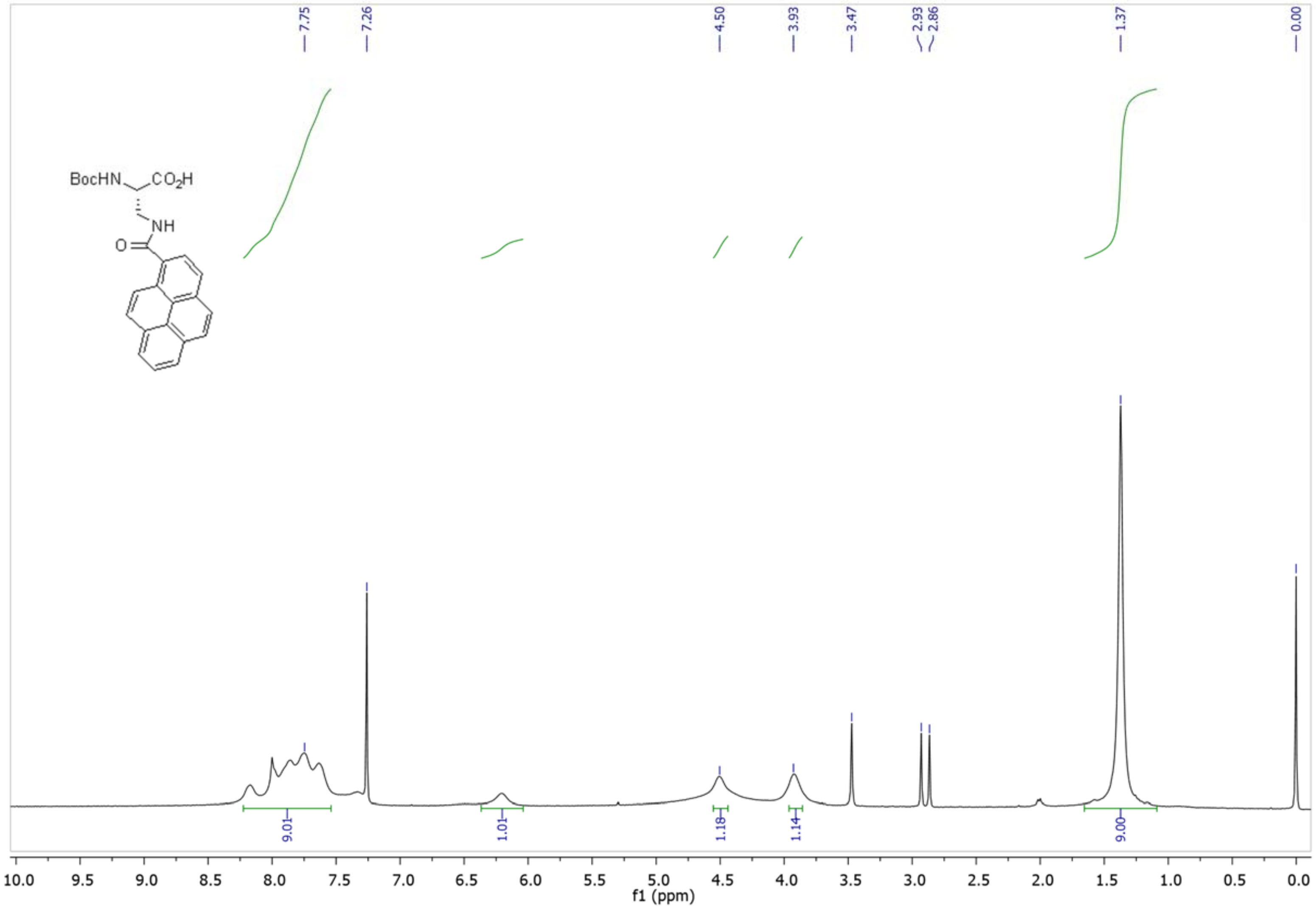

^{13}C NMR ($CDCl_3$, 151 MHz) of **4**

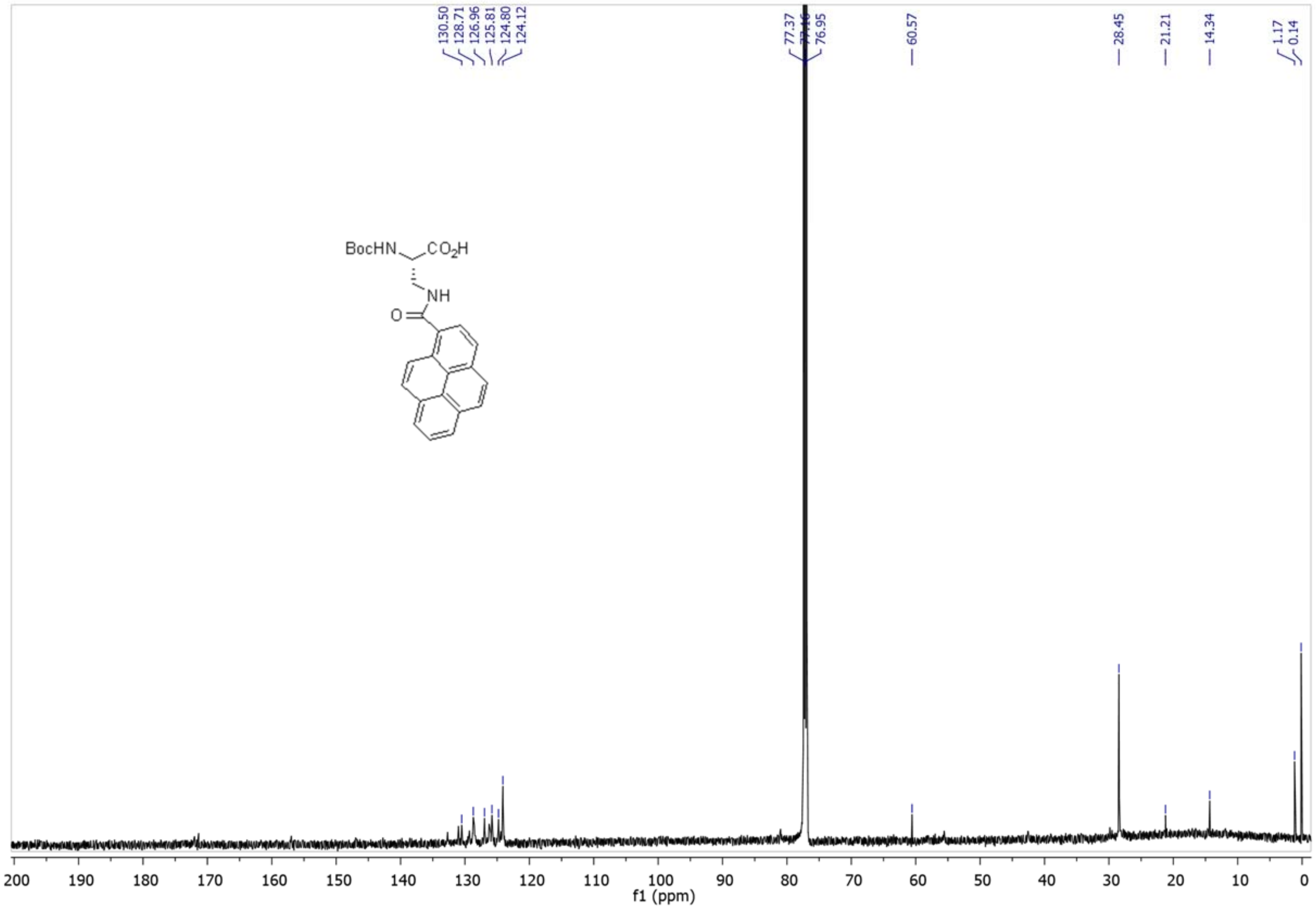

HRMS of **4**

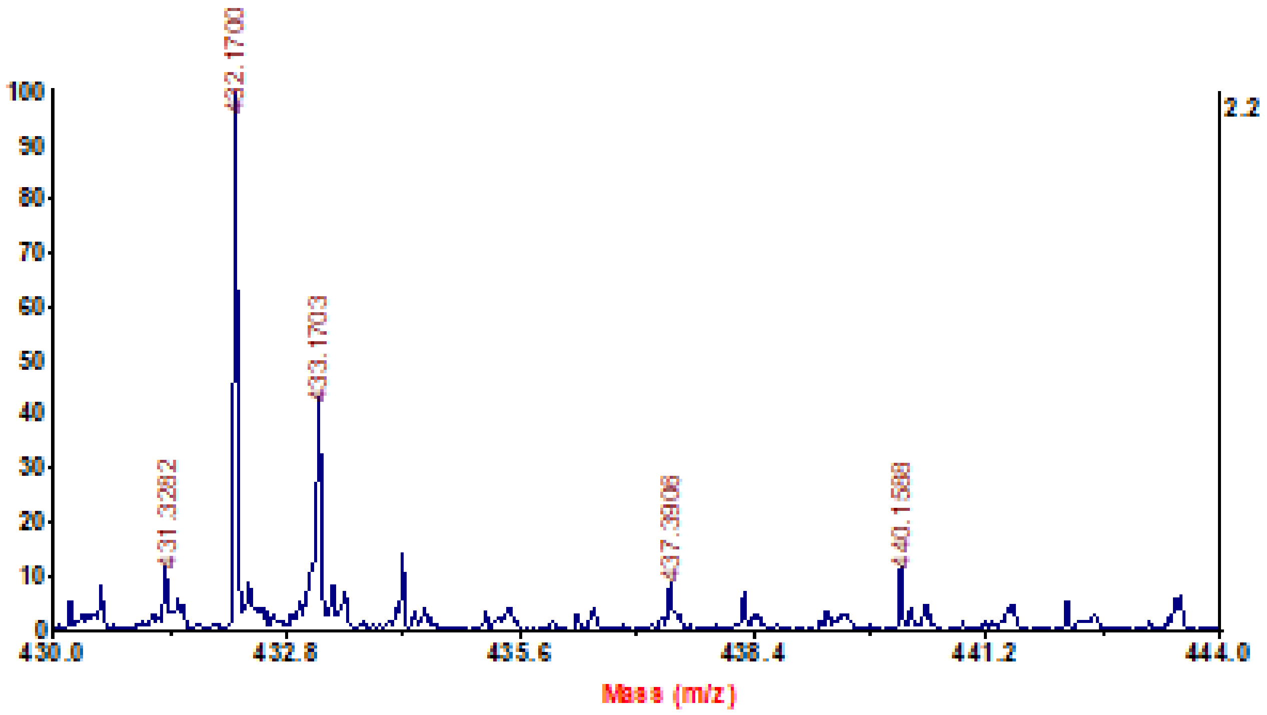

432.1700
433.1703
431.3282
437.3906
440.1588
100
90
80
70
60
50
40
30
20
10
0
2.2
430.0
432.8
435.6
438.4
441.2
444.0
Mass (m/z)

^{1}H NMR (CD_3OD, 600 MHz) of **5**

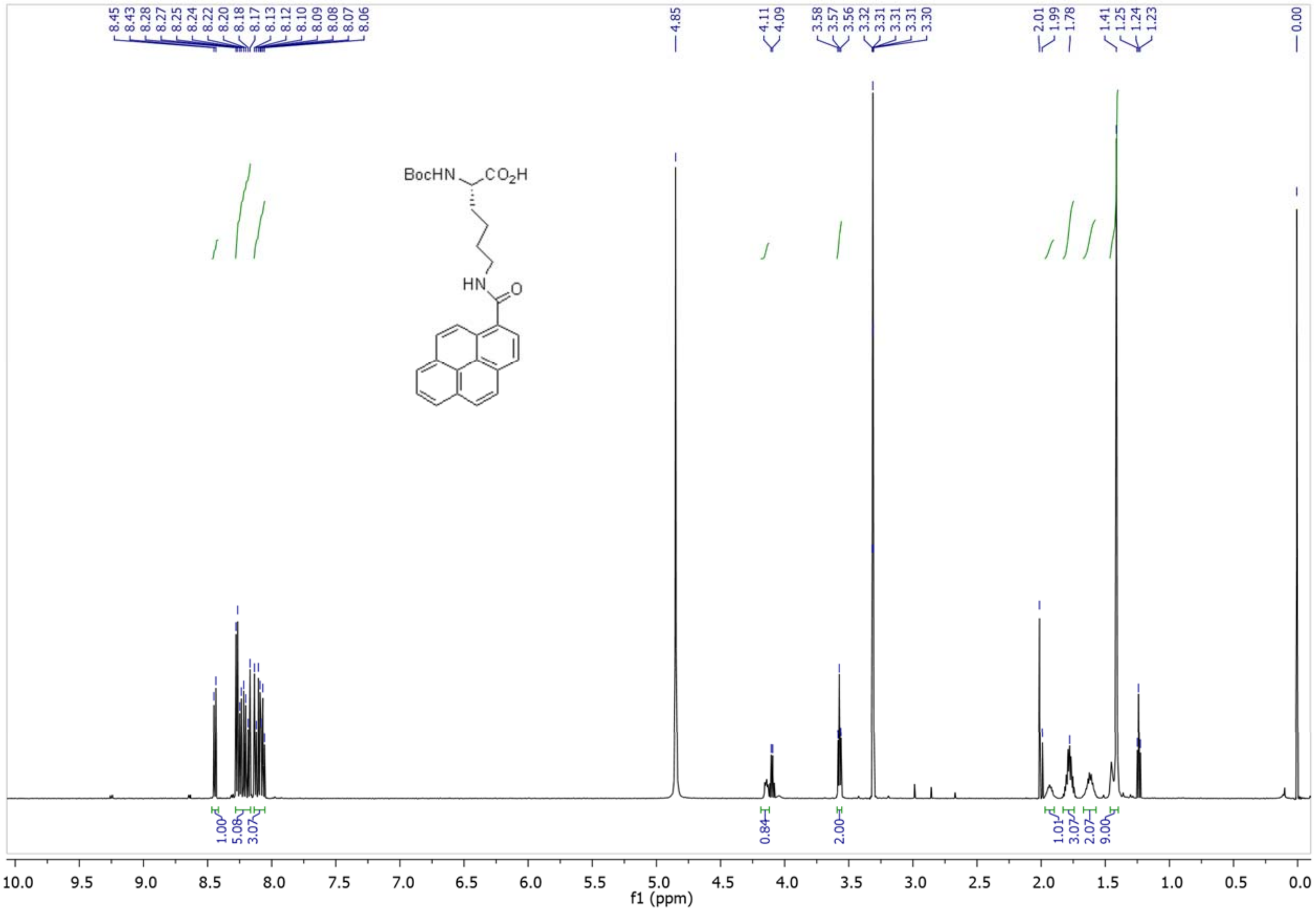

^{13}C NMR (CD_3OD, 151 MHz) of **5**

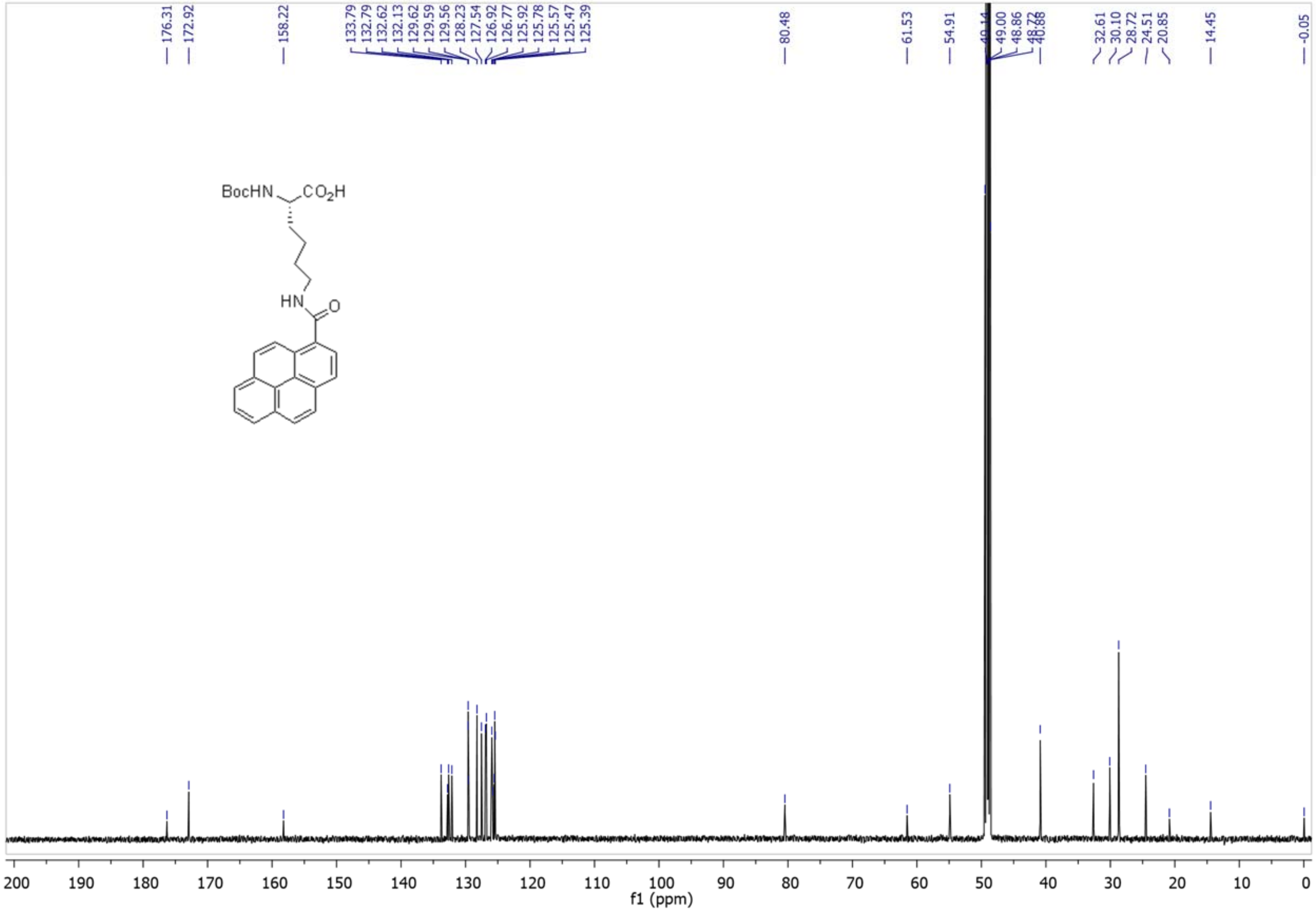

HRMS of **5**

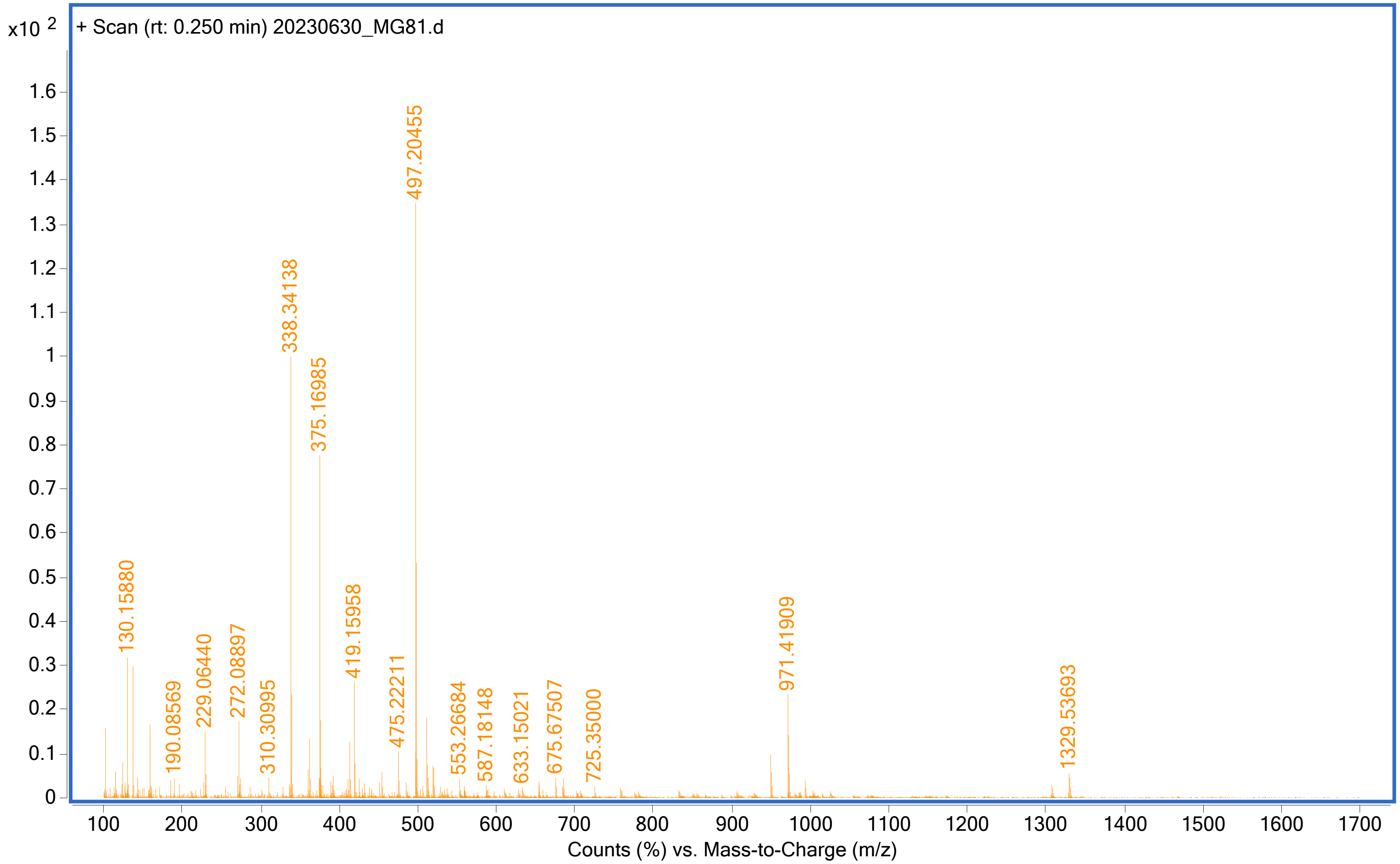

x10 2
+ Scan (rt: 0.250 min) 20230630_MG81.d
1.6
1.5
1.4
1.3
1.2
1.1
1
0.9
0.8
0.7
0.6
0.5
0.4
0.3
0.2
0.1
0
130.15880
190.08569
229.06440
272.08897
310.30995
338.34138
375.16985
419.15958
475.22211
497.20455
553.26684
587.18148
633.15021
675.67507
725.35000
971.41909
1329.53693
100
200
300
400
500
600
700
800
900
1000
1100
1200
1300
1400
1500
1600
1700
Counts (%) vs. Mass-to-Charge (m/z)

^{1}H NMR ($CDCl_3$, 600 MHz) of **7**

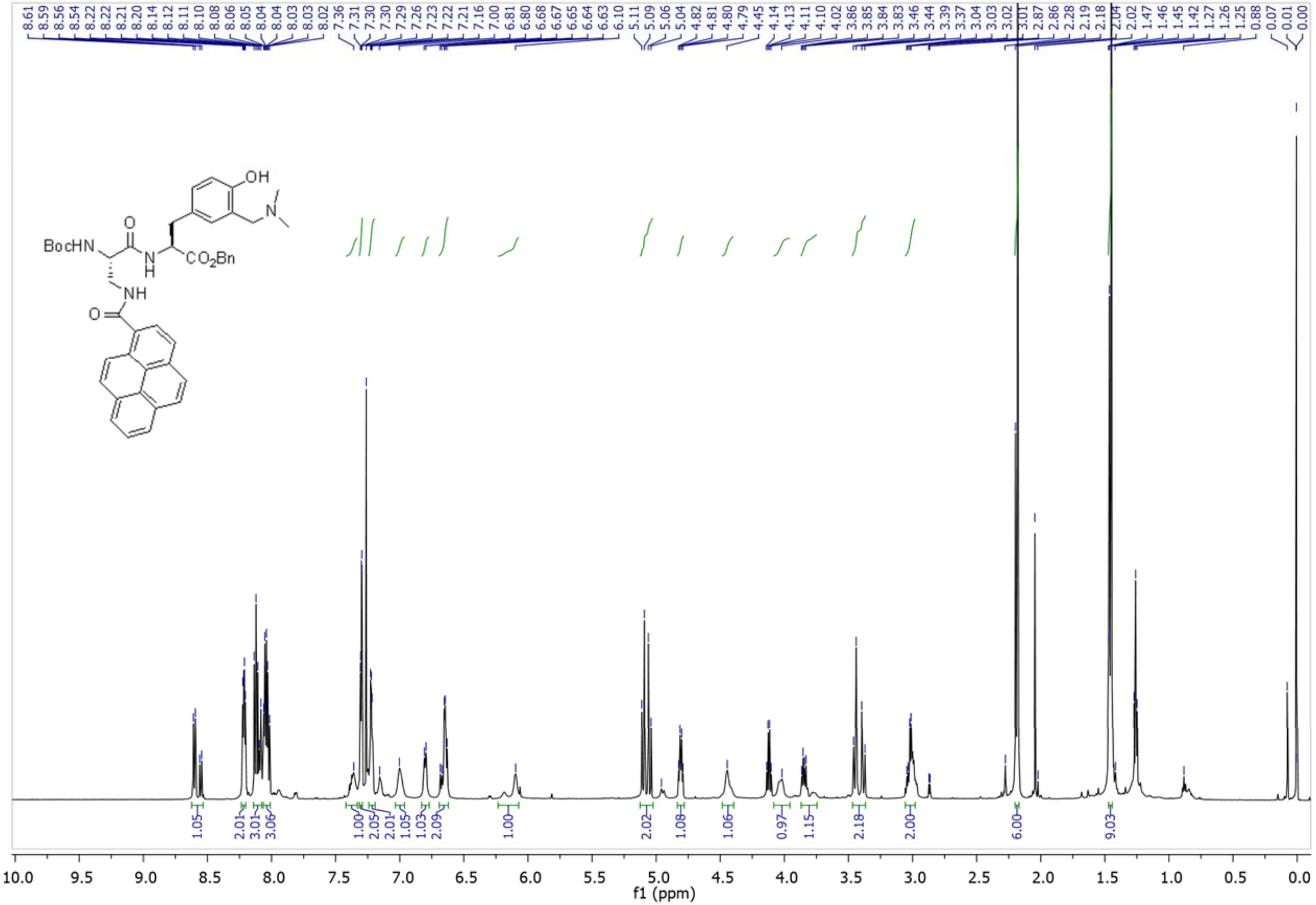

^{13}C NMR ($CDCl_3$, 151 MHz) of **7**

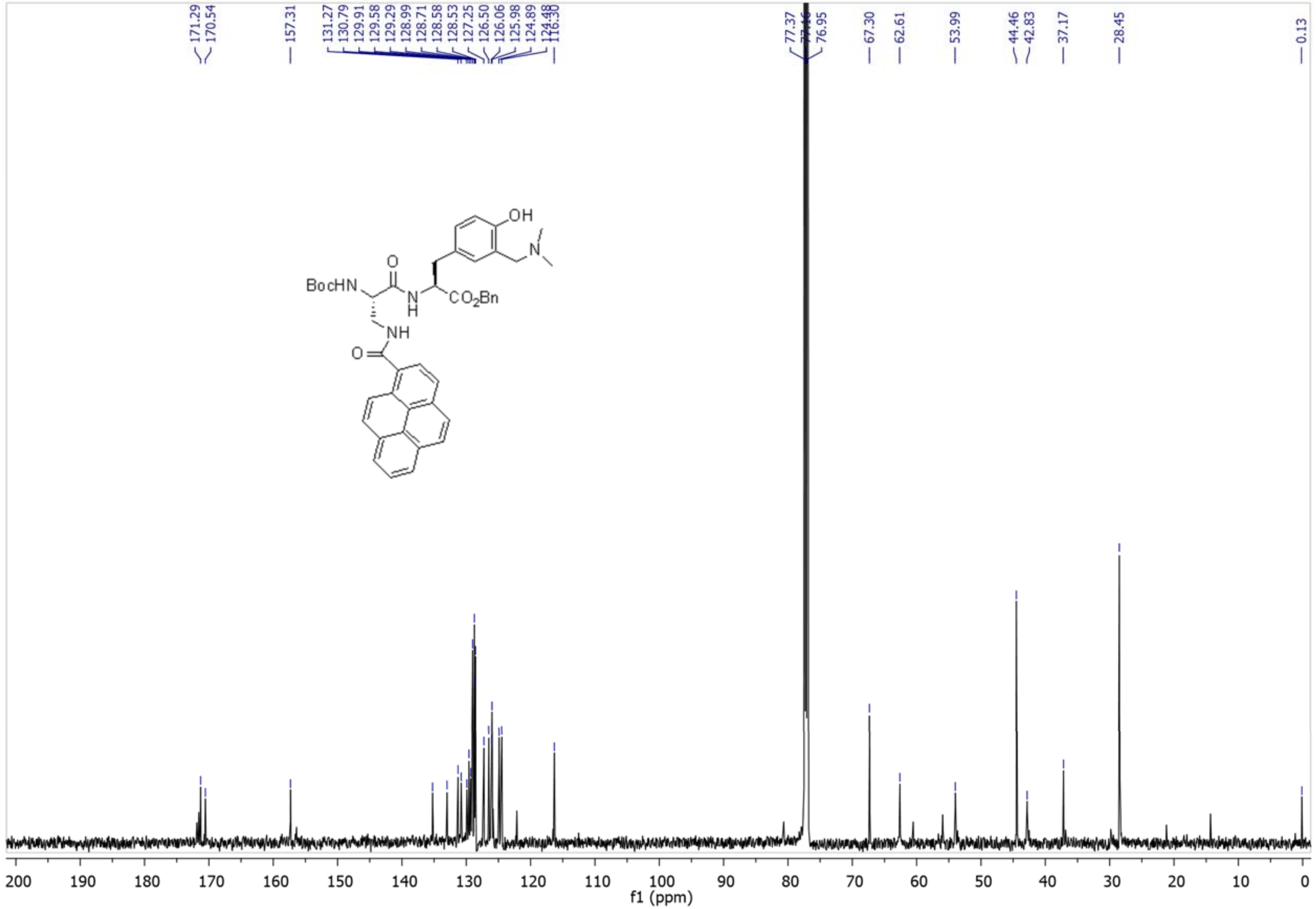

HRMS of **7**

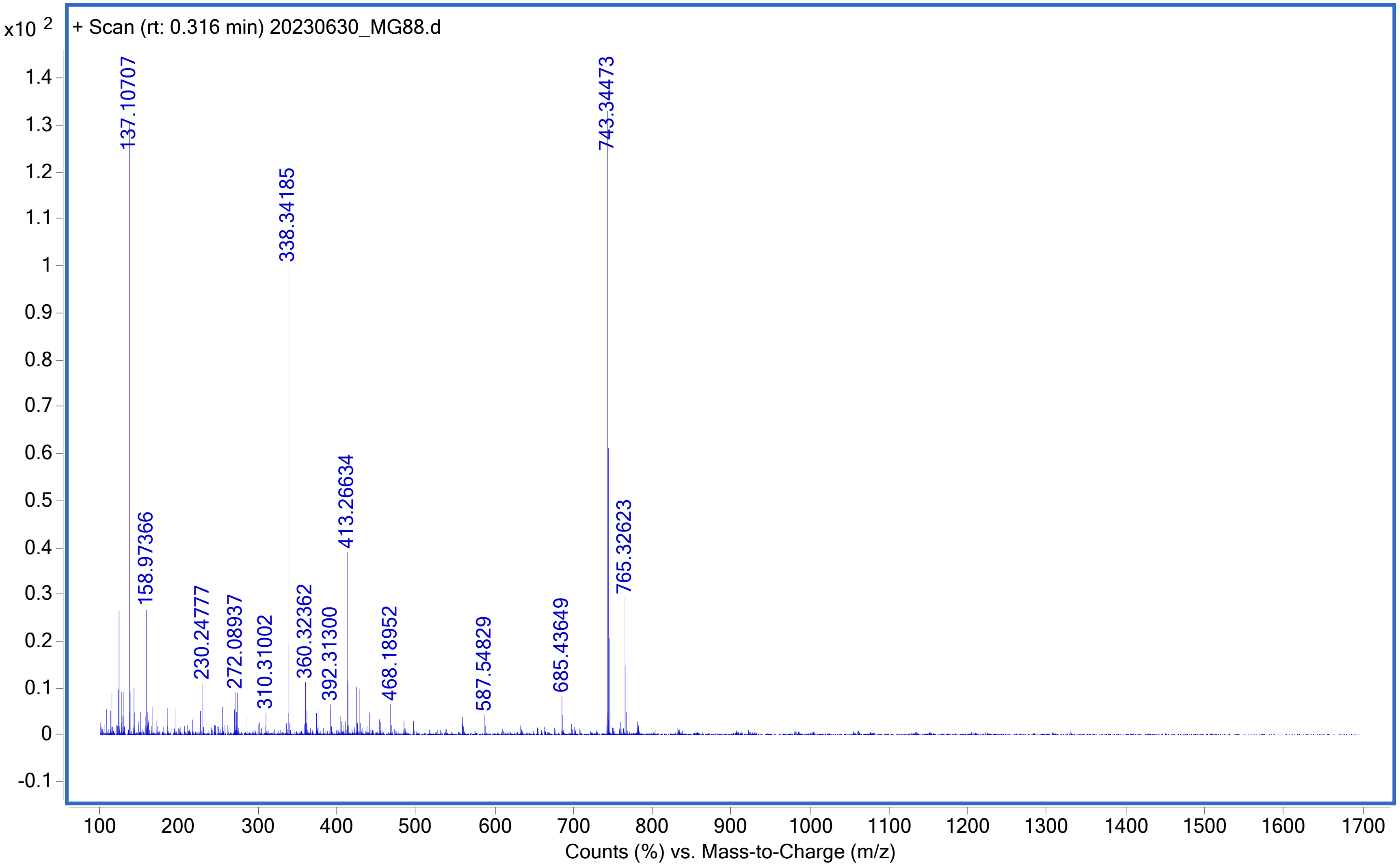

x10 2
+ Scan (rt: 0.316 min) 20230630_MG88.d
137.10707
743.34473
338.34185
413.26634
765.32623
158.97366
230.24777
272.08937
310.31002
360.32362
392.31300
468.18952
587.54829
685.43649
Counts (%) vs. Mass-to-Charge (m/z)

^{1}H NMR (CD_3OD, 300 MHz) of **8**

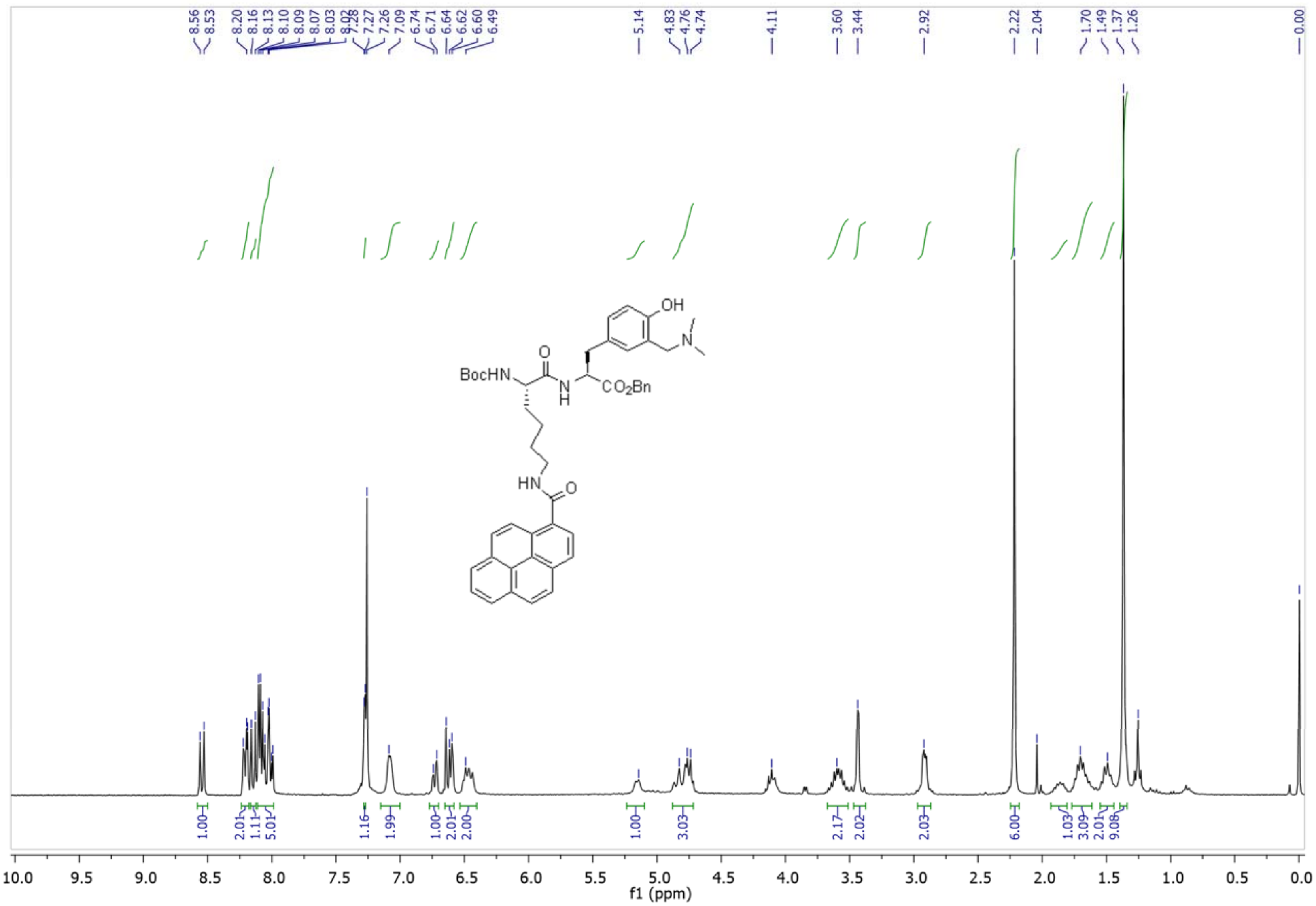

^{13}C NMR (CD_3OD, 151 MHz) of **8**

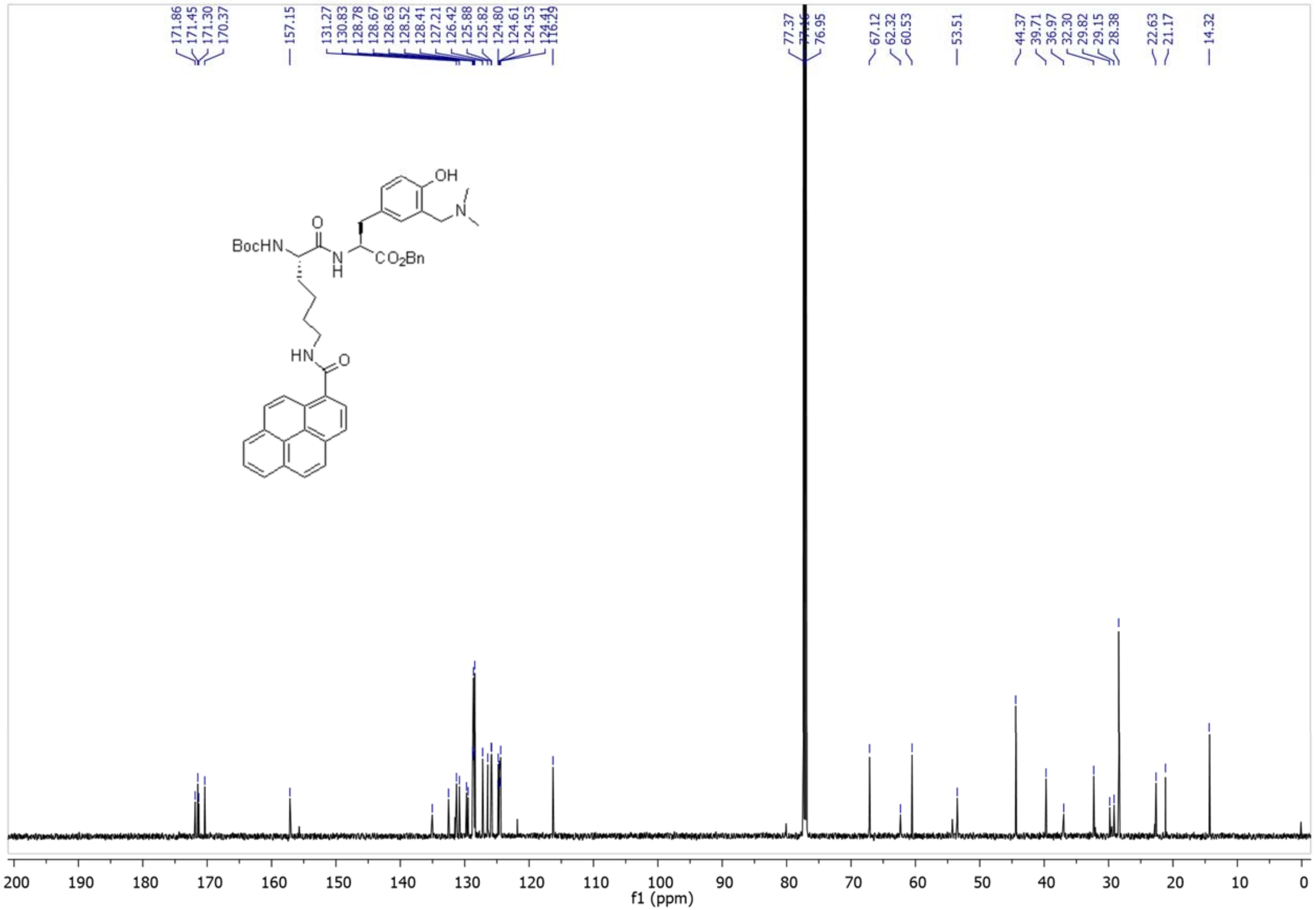

HRMS of **8**

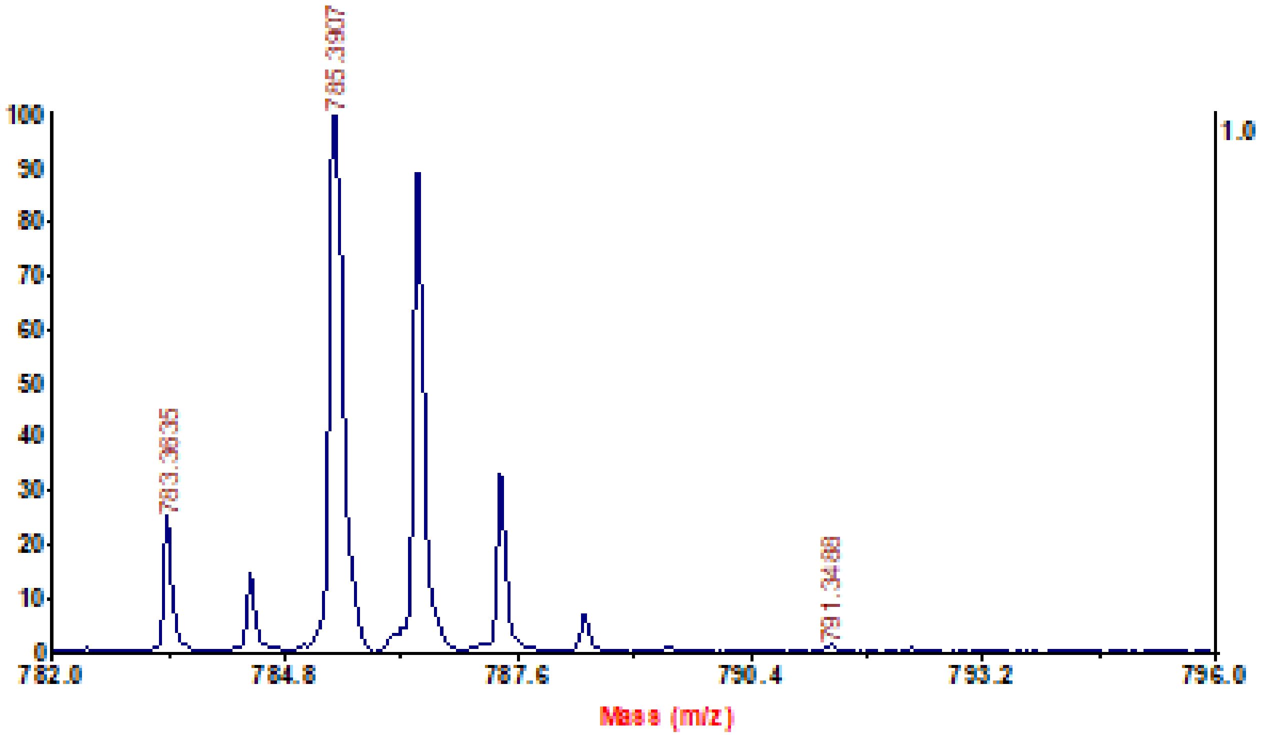

785.3907
783.3835
791.3488
100
90
80
70
60
50
40
30
20
10
0
1.0
782.0
784.8
787.6
790.4
793.2
796.0
Mass (m/z)

^{1}H NMR ($CDCl_3$, 600 MHz) of **7**-OCH_3

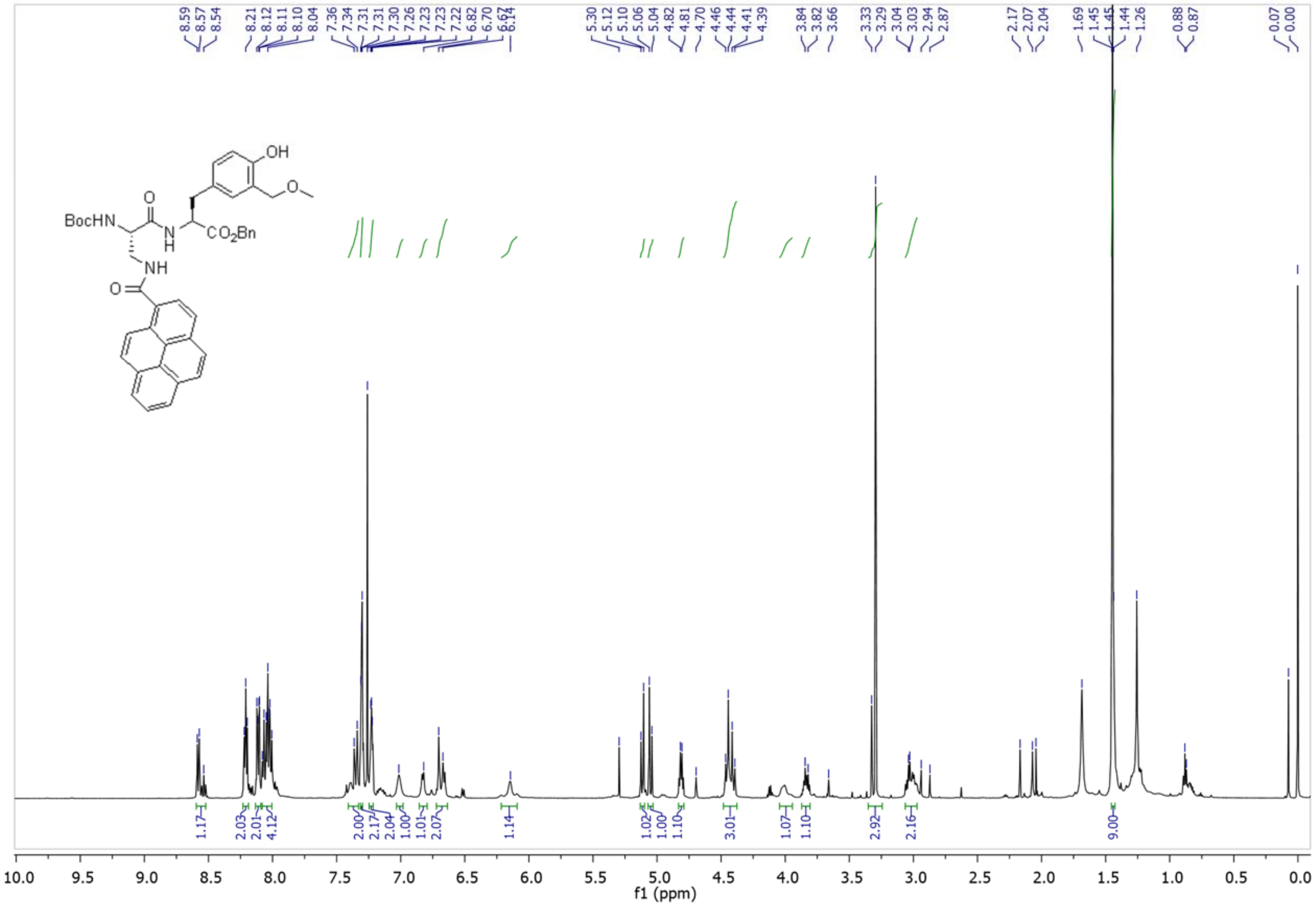

^{13}C NMR ($CDCl_3$, 151 MHz) of **7**-OCH_3

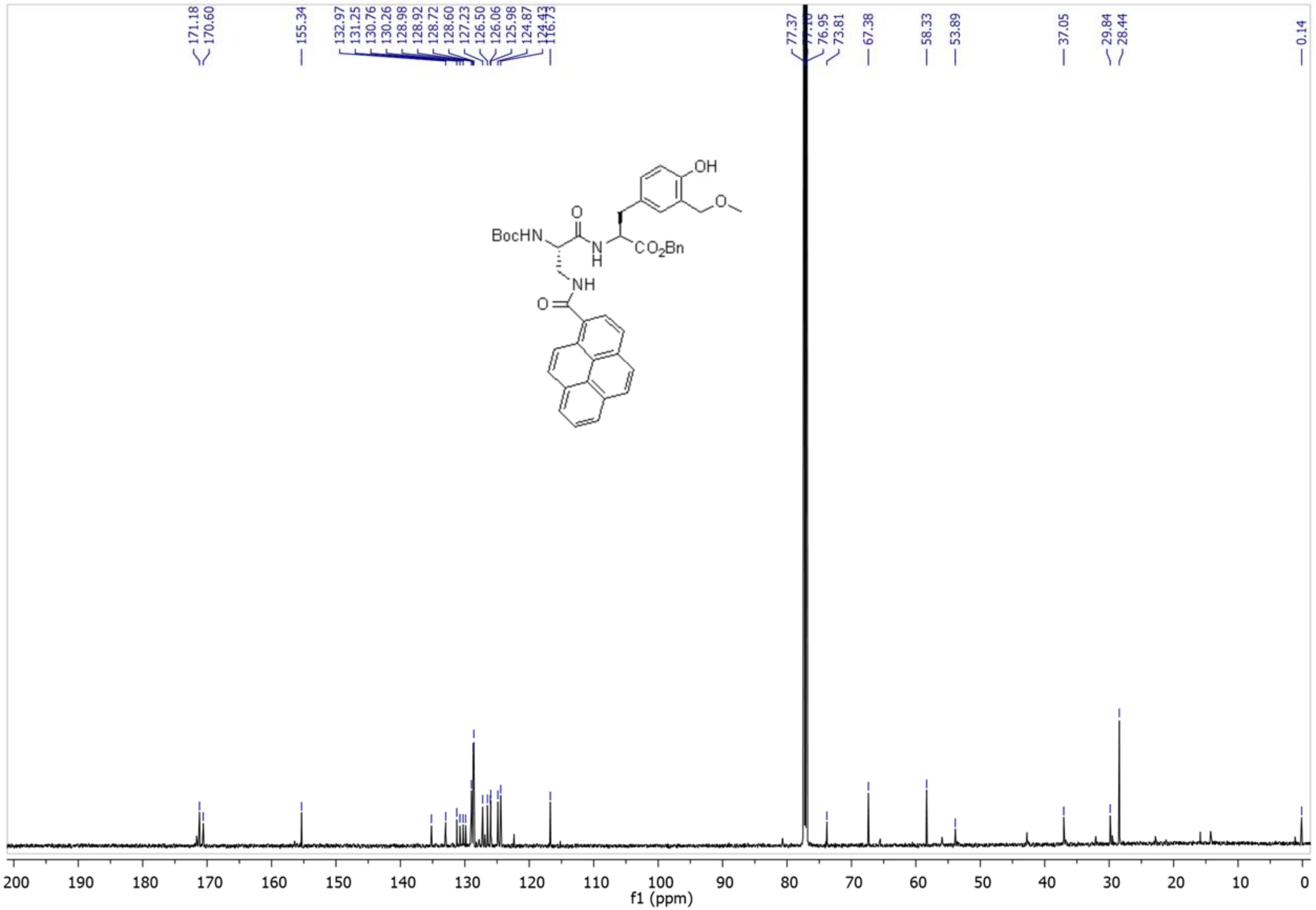

HRMS of **7**-OCH_3

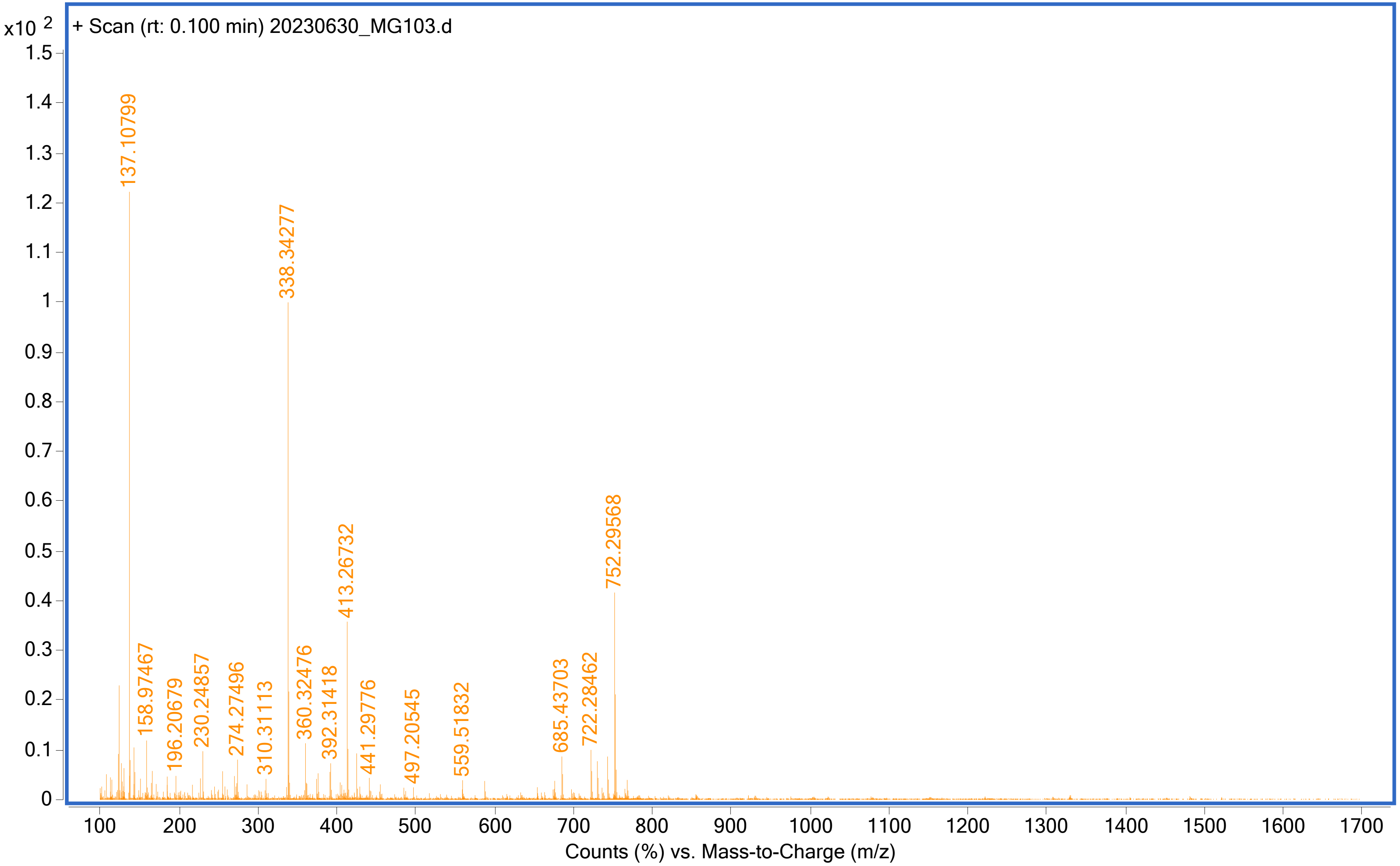

^{1}H NMR (CD_3OD, 600 MHz) of **8**-OCH_3

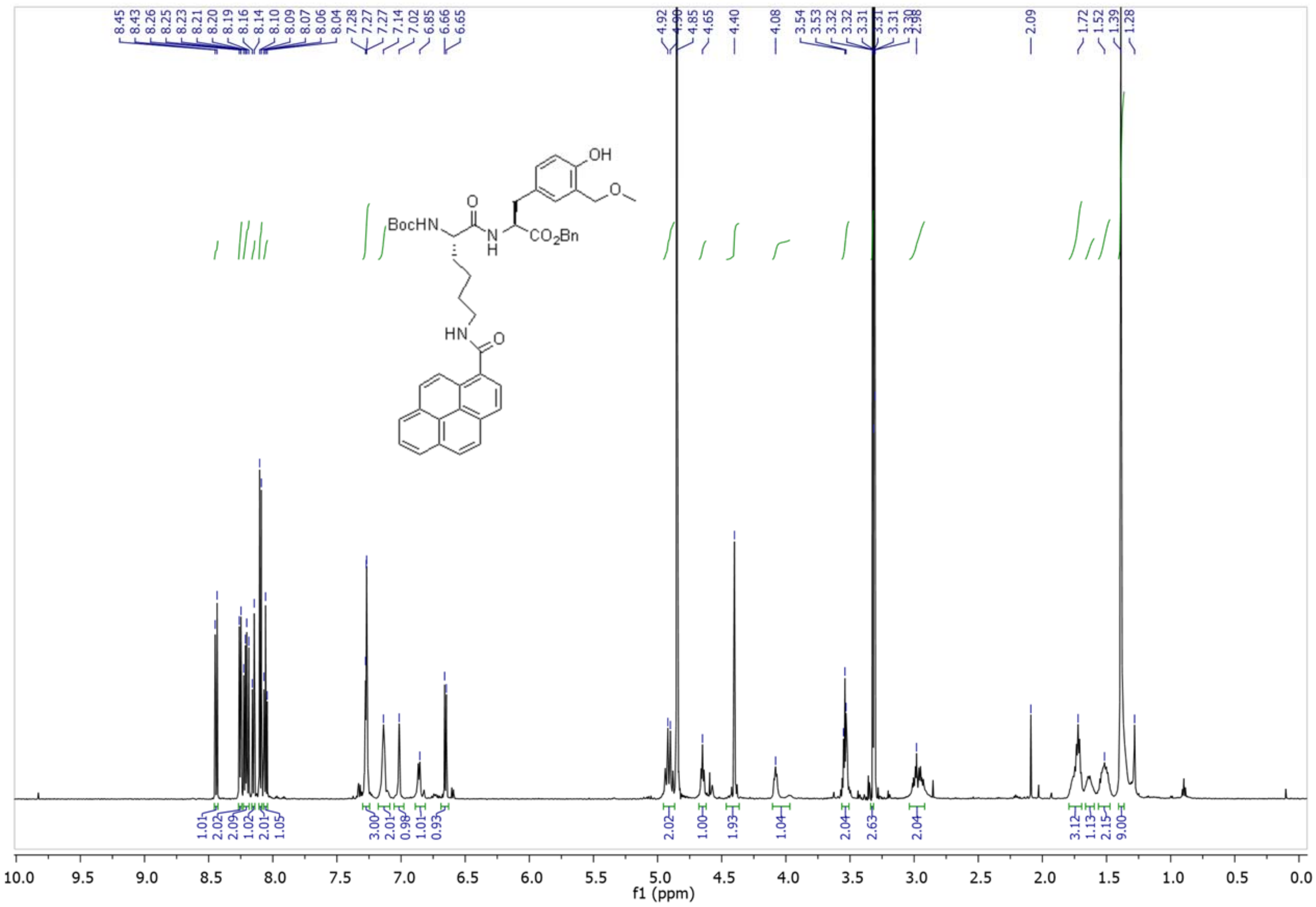

^{13}C NMR (CD_3OD, 151 MHz) of **8**-OCH_3

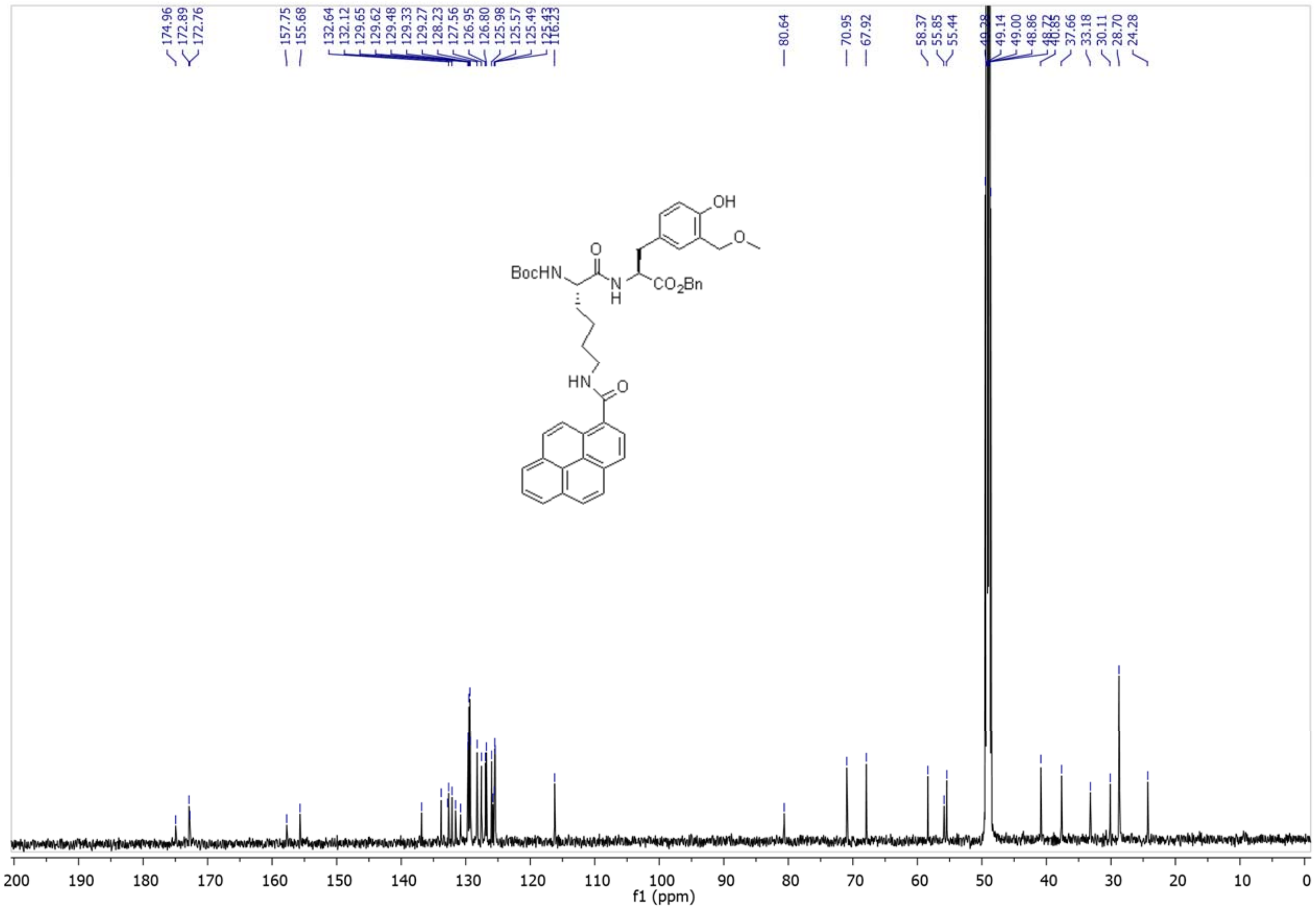

HRMS of **8**-OCH_3

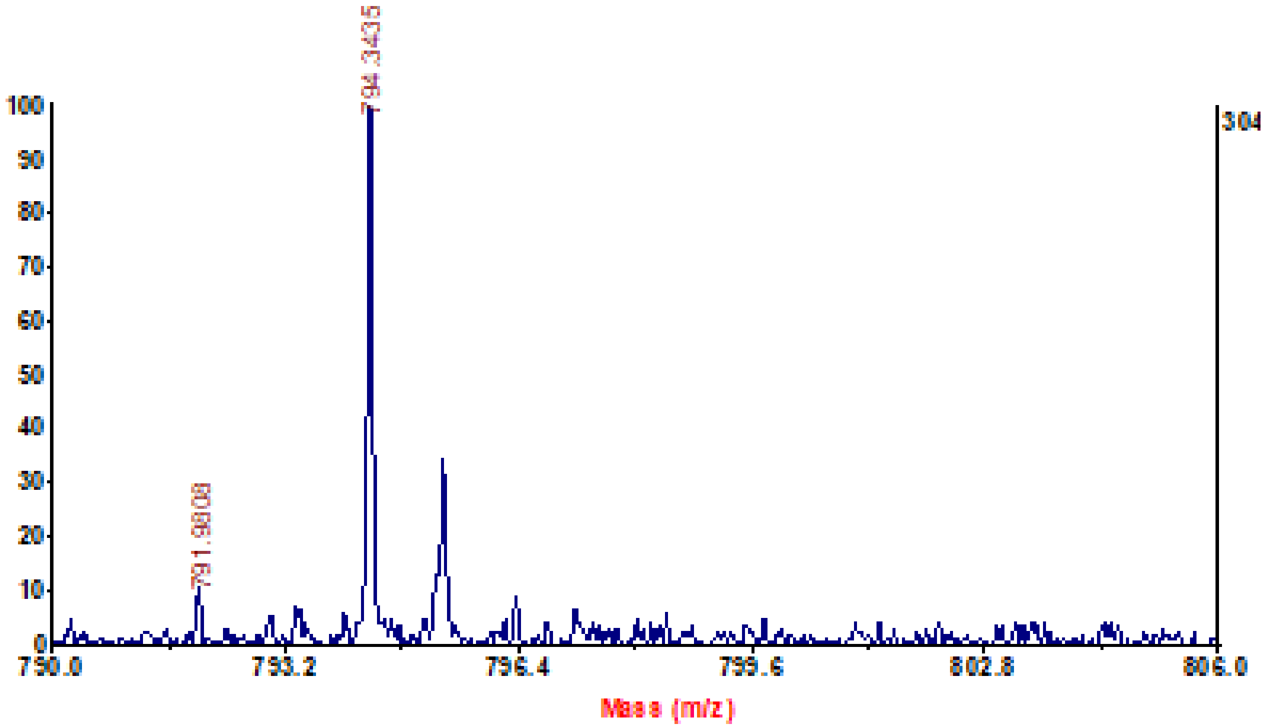

6. References

[1] a) Dendrinos, K. G.; Kalivretenos, A. G., Synthesis of *N*-hydroxysuccinimide esters using polymer bound HOBt, *Tetrahedron Lett.* **1998**, *39*, 1321-1324.

[2] Li, X.; Zhao, Y., Oligocholate foldamers with 'prefolded' macrocycles for enhanced folding in solution and surfactant micelles, *Tetrahedron* **2013**, *69*, 6051-6059.

[3] Husak, A.; Noichl, B. P.; Šumanovac Ramljak, T.; Sohora, M.; Škalamera, Đ.; Budiša, N.; Basarić, N., Photochemical formation of quinone methides from peptides containing modified tyrosine, *Org. Biomol. Chem.* **2016**, *14*, 10894-10905.

[4] Montalti, M.; Credi, A.; Prodi, L.; Gandolfi, M. T. In *Handbook of Photochemistry*; CRC Taylor and Francis: Boca Raton, 2006.

[5] Goldstein, S.; Rabani, J. The Ferrioxalate and Iodide-Iodate Actinometers in the UV Region. *J. Photochem. Photobiol.* **2008**, *193*, 50-55.

[6] Saenger, W. Principles of Nucleic Acid Structure; Springer-Verlag: New York, USA, 1983.

[7] Cantor, C.R.; Schimmel; P.R. Biophysical Chemistry, WH Freeman and Co.: San Francisco, USA, 1980; pp. 1109-1181.

[8] McGhee, J. D.; von Hippel, P. H. Theoretical Aspects of DNA-Protein Interactions: Co-operative and Non-co-operative Binding of Large Ligands to a one-Dimensional Homogeneous Lattice. *J. Mol. Biol.* **1976**, *103*, 679-681.

[9] McGhee, J. D.; von Hippel, P. H. Theoretical Aspects of DNA-Protein Interactions: Co-operative and Non-co-operative Binding of Large Ligands to a one-Dimensional Homogeneous Lattice. *J. Mol. Biol.* **1976**, *103*, 679-681.
